# A Novel Metabolism-Related Gene Signature for Predicting the Prognosis of HBV-Infected Hepatocellular Carcinoma

**DOI:** 10.1155/2022/2391265

**Published:** 2022-08-28

**Authors:** Zhenfu Gao, Jingyun Chen, Yebin Zhou, Pan Deng, Lu Sun, Jun Qi, Ping Zhang

**Affiliations:** ^1^Department of Hepatobiliary and Pancreatic Surgery, The First Hospital of Jilin University, No. 1 Xinmin Street, Changchun 130021, Jilin Province, China; ^2^Department of Thyroid Surgery, The First Hospital of Jilin University, No. 1 Xinmin Street, Changchun 130021, Jilin Province, China

## Abstract

Metabolic reprogramming is one of the crucial hallmarks of cancer. Hepatocellular carcinoma (HCC) resulting from hepatitis B has various altered metabolic features. However, the impact of such alterations on the tumor microenvironment (TME) and immunotherapy efficacy is still unclear. Here, a prognostic signature of metabolism-related gene (MRG) composition was constructed, and the immune profile of different subgroups and potential response to immunotherapy were described. Based on the HCC gene dataset, we used weighted gene coexpression network analysis for identifying MRGs linked to hepatitis B. An MRG prognostic index (MRGPI) with two genes, ATIC and KIF2C, was constructed using Cox regression analysis, an independent prognostic factor. In addition, the model was validated using the GEO dataset. The immune profile and prediction of HCC response to immunotherapy in different subgroups were analyzed using CIBERSORT and TIDE. Based on the outcomes, the distributions of memory B cells, monocytes, resting mast cells, and *M*0 macrophages in TME were different with a greater benefit of immunotherapy in the low MRGPI risk group. In addition, the MRGPI risk groups showed substantial differences in sensitivity to conventional drug therapy. This study concludes that MRGPI is an effective biomarker for predicting the prognoses of patients with HCC resulting from hepatitis B virus infections and determining the efficacy of immunotherapy and conventional medical therapy.

## 1. Introduction

Hepatocellular carcinoma (HCC) is the fifth most widely known malignancy, with 8.2% of deaths globally. With over 800,000 new cases and fatalities each year, it is only second to lung cancer [1]. The 5-year survival rate for HCC is 18%, next to pancreatic cancer [2]. Infection by the hepatitis B virus (HBV) is the primary cause of HCC, among others. It is responsible for >80% of all HCC incidences in China and other developing countries [3]. Despite the increasing diversity of treatment for early-stage HCC, most patients relapse [4]. In addition, hepatitis B is a risk factor for metastasis or HCC recurrence [5].

Recently, metabolic reprogramming in tumor development has increasingly gained attention. The liver is vital for the metabolism of sugars, lipids, and amino acids. Hence, HCC is a classic metabolism-related tumor, unlike others [6]. Tumor cell metabolism affects the progression of the tumor as well as the fate of other immune cells surrounding the area [7]. Several experiments have reported that tumor metabolites reduce the activation of dendritic cells and T cells and the transformation of monocyte migration and macrophage status [8–11], suggesting the enormous impact of metabolism on the tumor microenvironment (TME), immune response, and tumor development [12].

In recent years, immunotherapy played a pivotal role in the treatment of advanced HCC [13]. In the treatment of various malignancies, immune checkpoint inhibitor (ICI) therapy, either alone or in combination with other agents, has shown remarkable outcomes [14]. To minimize immunological tolerance and tumor cell growth, ICIs interfere with the programmed cell death-1 (PD-1)/programmed cell death-ligand 1 (PD-L1) signaling pathway and inhibit the cytotoxic *T* lymphocyte-associated antigen-4 [15]. However, the objective response rate of approximately 15%–20% for monotherapy of HCC is a significant limitation of these drugs [16]. Therefore, potential prognostic markers associated with therapeutic benefits and substantial implications for improving the therapeutic efficacy of patients with HBV-infected HCC require urgent identification.

Recently, a discovery investigated the role of metabolism-related genes (MRGs) in head and neck squamous cell carcinoma [17]. In this study, a prognostic signature consisting of MRGs was constructed for predicting the prognosis of HBV-infected HCC patients on immunotherapy and conventional drug therapy. The study focused on all MRGs in HCC transcriptional data, and the weighted gene coexpression network analysis (WGCNA) was employed for screening the central MRGs linked to HBV hepatitis along with patient prognosis. Based on this, an MRG prognostic index (MRGPI) was developed, and we studied its relationship with a tumor immune cell profile. Its prognostic ability on immunotherapy and other drug treatments in patients with HCC was examined and compared with microsatellite instability (MSI) and tumor immune dysfunction and exclusion (TIDE). The results suggest that the MRGPI is a potential prognostic biomarker that can help in predicting the prognosis of HBV-infected HCC patients as well as those receiving pharmacological and immunotherapeutic treatments.

## 2. Materials and Methods

### 2.1. Data Acquisition and Processing

RNA-sequencing (RNA-seq) data (Level 3) of HCC patients were obtained from The Cancer Genome Atlas (TCGA) database (https://portal.gdc.cancer.gov/), and 345 HCC samples with complete clinical information were retained, with overall survival (OS) >30 days. Clinical information, including OS, age, gender, grade, TNM, stage, and hepatitis, of HCC patients was provided by UCSC Xena (https://xena.ucsc.edu/) (Table 1). MRGs were downloaded from the KEGG (https://www.kegg.jp/kegg/pathway.html), Reactome (https://reactome.ncpsb.org/download-data), Human-GEM (https://github.com/SysBioChalmers/Human-GEM/tree/master/model), and BRENDA (https://www.brenda-enzymes.org/download_brenda_without_registration.php) databases, totaling 3937.

The HCC microarray dataset (GSE14520) was provided by Gene Expression Omnibus (GEO) (https://www.ncbi.nlm.nih.gov/geo/), and 218 patients with HBV-infected HCC with OS >30 days were chosen as the validation set for the model.

### 2.2. WGCNA

WGCNA is a biological method that describes the patterns of gene linkage between various samples systematically. WGCNA analysis allows the search for coexpressed gene modules and exploration of associations between gene networks and phenotypes of interest. MRGs from the KEGG, Reactome, Human-GEM, and BRENDA databases were analyzed using WGCNA to identify the hub genes. We developed a similarity matrix by measuring Pearson's correlation coefficients between two genes, followed by its transformation into a signed adjacency matrix of a network type using the scale-free topology criterion *R*^2^ = 0.9 and soft threshold *β* = 12. Afterward, the adjacency matrix was changed to a topological matrix, and the topological overlap matrix (TOM) was employed for describing the level of the link between genes. The genes were clustered at a 1-TOM distance, and a dynamic pruning tree was developed for identifying the modules. In the end, module identification was performed with the “cutreeDynamic” function using the “tree” method (minModuleSize = 50 and cut height = 0.25) respectively. In addition, five modules (blue, brown, green, yellow, and grey) were identified, with the grey module grouped with non-coexpressed genes. Furthermore, we assessed the link between module eigengenes and clinical traits and identified the modules linked to HBV infection.

### 2.3. Construction and Validation of MRGPI

With *p* values <0.01 classified as survival-related genes, univariate Cox regression analysis identified metabolically relevant key genes in the coexpression module associated with hepatitis characteristics and survival status in patients with HBV infection. Genes linked to survival were incorporated into the multivariate Cox regression analysis, and MRGPI scores were calculated using a summation of the expression value products of each prognostic gene and the corresponding coefficient *b* of the associated weights. Patients were categorized into two groups as per their median MRGPI score: high MRGPI risk and low MRGPI risk. The risk score formula was as follows: MRGPI Score = EXP of Gene1 × *b*1 + EXP of Gene2 × *b*2 +···+EXP of Gene*n* × *bn*. Based on the multifactorial Cox regression analysis, the identified prognostic genes were constructed individually into corresponding OS prognostic models. KM survival curves determined whether the prognostic models differentiated the prognosis of patients. We calculated the area under the ROC curve for evaluating the prognostic model at each survival stage. The prognostic power of the MRGPI and clinical features of HBV patients were assessed using univariate and multivariate Cox regression analyses to check if the MRGPI could act as an independent prognostic factor.

### 2.4. Identification of Molecular Features between MRGPI Risk Groups

We employed the *R* package “edgeR” for comparing the high (*n* = 26) and low (*n* = 26) MRGPI risk groups to find differentially expressed genes using the criteria of |log2 (fold change)| >1 and adj.*p* values <0.05 (corrected by the FDR method). Moreover, we carried out gene set enrichment analysis (GSEA) using the *R* package “ClusterProfiler” for identifying the signaling pathways involved in the differentially expressed genes in the two groups (*p* values <0.05).

### 2.5. Assessment of the Immune Cell Infiltration Level between MRGPI Risk Groups

The tool CIBERSORT is used for the deconvolution of expression matrices in the subtypes of human immune cells according to the principle of the linear support vector regression [18]. This approach is based on its default provision of gene expression signature sets for 22 immune cell subtypes. The mRNA expression matrix was imported into CIBERSORT (https://cibersort.stanford.edu/) and iterated 1000 times to assess the proportion of immune cell subtypes in each cancer sample of patients with HBV infection. Variations in the level of immune cell infiltration in the two MRGPI risk groups were measured with the help of the Wilcoxon rank-sum test (*p* values <0.05), and the correlations between the immune cells were calculated using the Spearman correlation (*p* values <0.05).

### 2.6. Prediction of the Effect of Immunotherapy between MRGPI Risk Groups

A computational method called TIDE (https://tide.dfci.harvard.edu/) model tumor immune evasion by combining expression signals of *T* cell malfunction and T cell exclusion [19, 20]. TIDE was used to determine the degree of an individual's response to immunotherapy, and the difference in TIDE scores in the two MRGPI risk groups was measured using the Wilcoxon rank-sum test (*p* values <0.05).

### 2.7. Prediction of the Effect of Drug Treatment between MRGPI Risk Groups

The Genomics of Drug Sensitivity in Cancer (https://www.cancerrxgene.org/) (GDSC) database was utilized for predicting how samples would respond to drug therapy [21]. The procedure was performed with the help of the *R* package “pRRophetic,” which used ridge regression to estimate the samples' IC50 values and 10-fold cross-validation of the GDSC training set for the purpose of assessing the prediction accuracy. All parameters were set by the default values, and the tissue type is “allSoldTumours.” The IC50 values of samples from the two MRGPI risk groups were predicted with the help of four medications: sorafenib, AZD6244, ABT.263, and A.443654. Furthermore, the Wilcoxon rank-sum test was employed for calculating the difference in response to the aforementioned drugs between the two MRGPI risk groups (*p* values <0.05).

### 2.8. Analysis of Functional Enrichment

The “ClusterProfiler” *R* package was utilized for conducting functional enrichment analysis of differentially expressed genes (DEGs). Through enrichment analysis (adj. *p* values <0.05, corrected for Benjamini & Hochberg technique), significantly enriched gene ontology (GO) items were found. KEGG pathway enrichment analysis (adjusted *p* value <s 0.05, corrected for Benjamini & Hochberg technique) was utilized for finding significantly enriched pathways.

## 3. Results

### 3.1. Hepatitis B-Related Coexpressed Metabolic Modules

**Figure 1:**
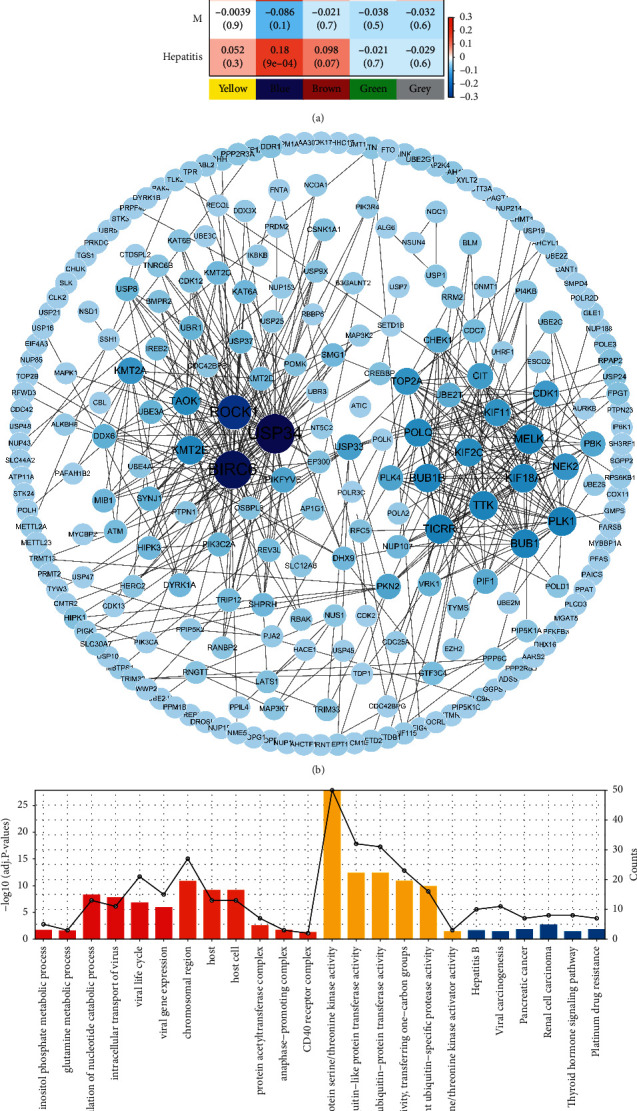
Identification of coexpressed metabolic genes associated with HBV hepatitis using WGCNA. (a) Heatmap of the correlation between gene modules and clinical traits of HCC. (b) Coexpression network of blue module genes. (c) GO and KEGG enrichment analyses of genes in the blue coexpression network.

WGCNA analysis was performed on MRGs from the KEGG, Reactome, Human-GEM, and BRENDA databases to obtain metabolically relevant coexpressed genes. The optimal soft threshold power of the scale-free network was 12 based on the logarithm log (*k*) of the node, and connectivity K was negatively linked to the logarithm log (*P* (*k*)) of the probability of that node, with a correlation coefficient >0.90 (Supplementary Figure 1(a)). We found five modules as per the mean linkage hierarchical clustering and optimal soft threshold power (Supplementary Figure 1(b)). We assigned a total of 3937 genes to the five modules (blue: 1010, brown: 188, green: 109, yellow: 181, and grey: 2449) (Supplementary Table 1), and the association between the modules and six clinical traits, including age, gender, grade, TNM, stage, and hepatitis, was analyzed. The results revealed the remarkable link of the blue module with HBV infection (Cor = 0.18, *p* value = 9*e* − 04), *T* (Cor = 0.24, *p* value = 1*e* − 05), *N* (Cor = 0.21, *p* value = 8*e* − 05), stage (Cor = 0.23, *p* value = 1*e* − 05), and grade (Cor = 0.16, *p* value = 0.003), and it was positively correlated with other features (Figure 1(a)). Therefore, genes from the blue module were chosen for analysis in detail.

We found a total of 248 genes and 515 edges in the network of blue modules via the selection of pairs of relationships with threshold weights >0.05 (Figure 1(b)). The GO and KEGG enrichment analyses of the genes in the blue module network indicated significant enrichment in various biological processes and signaling pathways, including viral processes, metabolic processes, and hepatitis B (Figure 1(c)).

### 3.2. Prognostic Significance of Metabolism-Related Key Genes in Patients with HBV-Infected HCC

**Figure 2:**
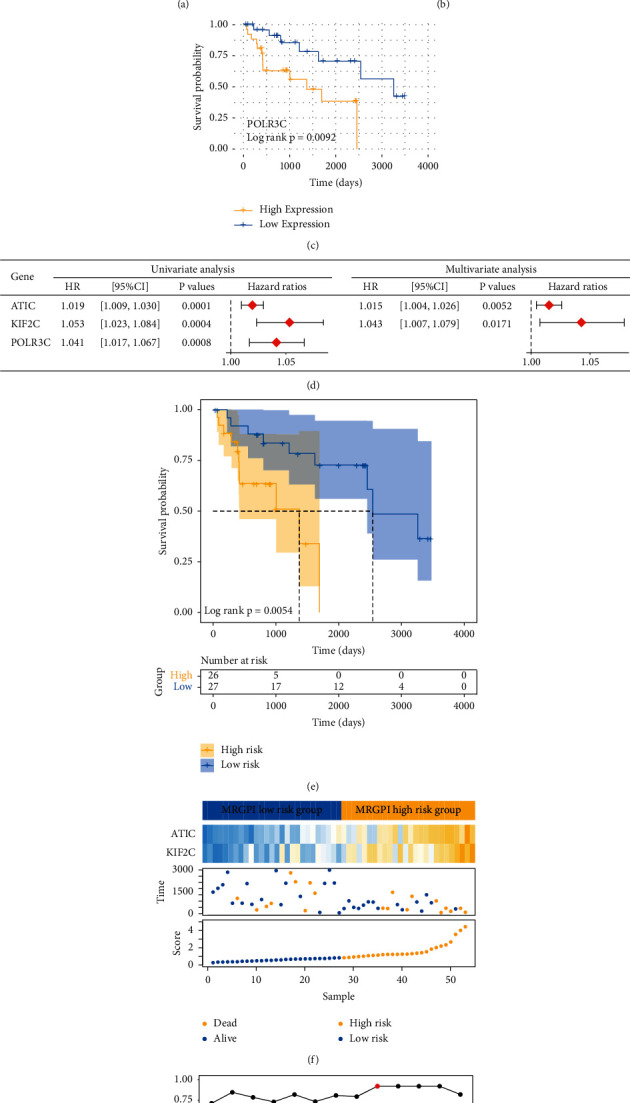
Construction of MRGPI in HCC. (a–c) Survival curves of ATIC, KIF2C, and POLR3C survival-related genes. (d) Comparison of univariate and multivariate Cox regression analyses of survival-related genes and independent prognostic markers. (e) Survival curves of prognostic models in patients with HBV infection. (f) Distribution of ATIC and KIF2C gene expressions, MRGPI scores, and survival status in each sample. (g) AUC values for prognostic models at different time intervals.

To investigate the prognostic values of metabolism-related key genes (*n* = 248) in the coexpression module (blue module) associated with hepatitis HBV in patients with HBV infection, univariate and multivariate Cox regression analyses were performed on these variables, respectively. Three metabolism-related key genes, including ATIC, KIF2C, and POLR3C, were considerably linked to survival and identified in HBV-infected HCC patients using univariate Cox regression analysis (Figures 2(a)–2(c)). Multifactorial Cox regression analysis was performed for identifying independent prognostic genes in HBV-infected HCC patients, showing that ATIC and KIF2C were two independent prognostic genes that exhibited high expression of high risk (Figure 2(d)). Furthermore, the prognostic index was constructed for all samples and calculated as MRGPI Score = EXP of ATIC × (0.0150) + EXP of KIF2C × (0.0417). The Kaplan–Meier survival curve revealed that HBV-infected HCC patients in the low MRGPI risk group had remarkably better survival in comparison with those in the high MRGPI risk group (*p* value = 0.0053) (Figures 2(e) and 2(f)). We carried out the evaluation of the predictive performance of the prognostic model with the help of the ROC curve, indicating good predictive ability with an area under the ROC curve (AUC) of 0.919 at 5 years. In addition, the AUC values for 1, 3, and 7 years were 0.712, 0.818, and 0.819, respectively (Figure 2(g)).

Moreover, the GEO dataset GSE14520 (*n* = 218) was used to validate ATIC, KIF2C, and POLR3C genes and the ability of the prognostic model. ATIC (*p* value = 0.00012), KIF2C (*p* value = 0.0054), and POLR3C (*p* value = 0.0092) showed significant correlations with survival (Supplementary Figures 2(a)–2(c)). In the prognostic model, patients in the low MRGPI risk group showed remarkably better survival than those in the high MRGPI risk group (*p* value = 0.0074) (Supplementary Figure 2(d)). This result is consistent with that from the TCGA LIHC dataset. In addition, the validation set model was evaluated using ROC curves, with an AUC value of 0.612 at 5 years (Supplementary Figure 2(e)).

### 3.3. Molecular Characterization of MRGPI Subgroups

**Figure 3:**
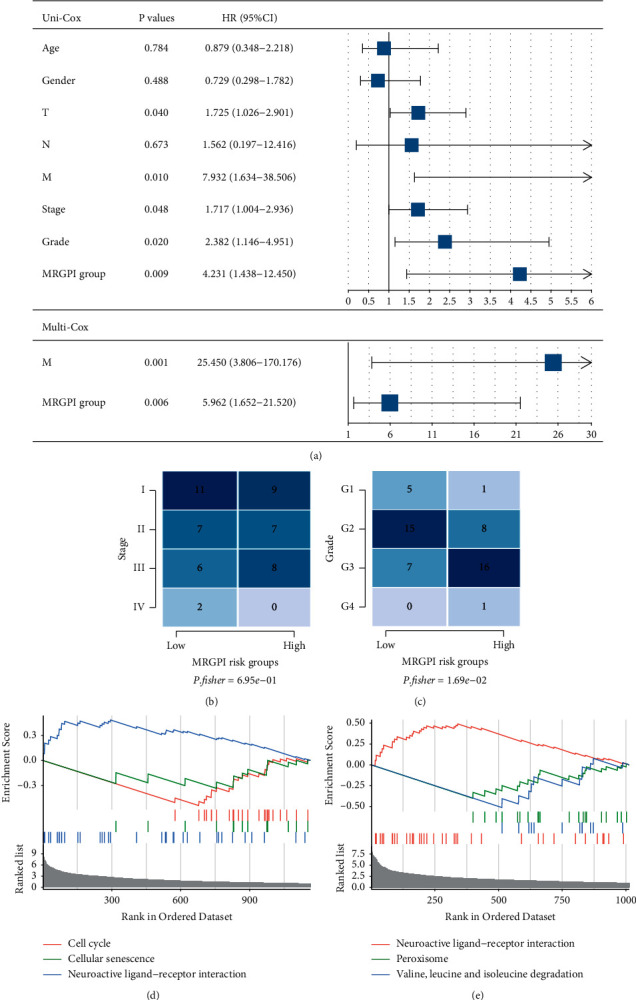
Characteristics of MRGPI subgroups. (a) Comparison of univariate and multivariate Cox regression analyses of MRGPI risk group characteristics and clinical information. (b, c) Heatmap of differences in stage and grade distributions between the high and low MRGPI risk groups. (d, e) Significant enrichment of GSEA pathways in the high and low MRGPI risk groups.

We carried out univariate and multifactorial Cox regression analyses on the MRGPI risk groups and clinical data such as gender, age, TNM, stage, and grade, to determine whether the MRGPI risk group is an independent prognostic factor for overall survival. In HBV patients, univariate Cox regression analysis revealed that the risk group was considerably linked to overall survival (HR = 4.2308347, 95% CI = 1.4377316–12.450141, *p* value = 0.0088). In addition, *T* (*p* value = 0.040), *M* (*p* value = 0.010), stage (*p* value = 0.048), and grade (*p* value = 0.020) were greatly linked to overall survival. A subsequent multifactorial Cox regression analysis revealed the MRGPI risk group being an independent prognostic factor was considerably linked to overall survival (HR = 5.961821, 95% CI = 1.651621–21.52026, *p* value = 0.006) (Figure 3(a)).

Clinical information and molecular characteristics were compared to explore differences between the two MRGPI risk groups. The results showed no significant differences in the stage distribution (Fisher's exact test, *p* value = 0.695) and significant differences in the grade distribution (Fisher's exact test, *p* value = 0.0169) (Figures 3(b) and 3(c)). DEGs between the high and low MRGPI risk groups were discovered using differential analysis, revealing that the expression levels of 1220 and 1079 genes, respectively, were elevated in the high and low MRGPI risk groups (Supplementary Table 2). Furthermore, GSEA enrichment analysis revealed that the gene set in the high MRGPI risk group was greatly enriched in seven related pathways, including neuroactive ligand-receptor interaction, cellular senescence, and cell cycle (Figure 3(d)), whereas the gene set in the low MRGPI risk group was significantly enriched in neuroactive ligand-receptor interaction, valine, leucine, and isoleucine degradation, peroxisome, and other related pathways (Figure 3(e)).

### 3.4. Immune Cell Compositions in MRGPI Risk Groups

**Figure 4:**
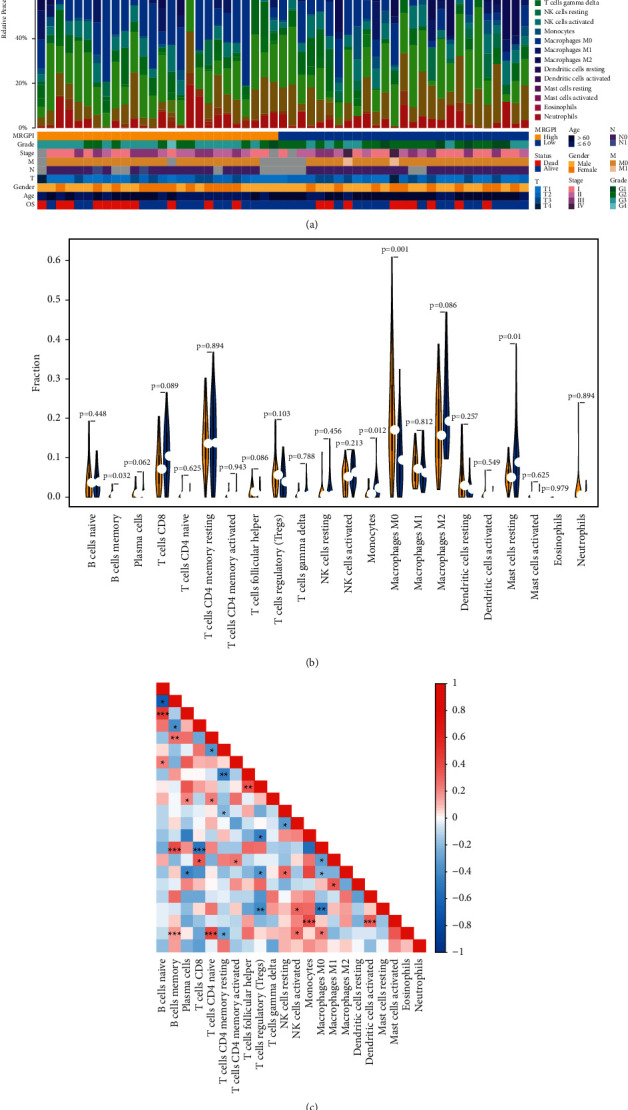
Immune cell compositions of MRGPI groups in TME. (a) Relative proportions of immune infiltration of 24 immune cell types in the MRGPI risk groups were calculated using the CIBERSORT method. (b) Differences in immune infiltration by immune cells in the MRGPI risk groups (yellow: high MRGPI risk group, blue: low MRGPI risk group). (c) Correlation patterns among immune cells in patients with HBV infection (ns: not significant; ^*∗*^*p* < 0.05; ^*∗∗*^*p* < 0.01; ^*∗∗∗*^*p* < 0.001).

The Wilcoxon test was employed for examining the distribution of immune cells in different MRGPI risk groups, and the CIBERSORT method was utilized to systematically analyze the infiltration levels of immune cells in the MRGPI risk groups.  Figure 4(a) describes the level of immune cell infiltration in the two MRGPI risk groups, as well as clinical data for each sample. Patients in the high MRGPI risk group had more memory B cells (*p* value = 0.032) and M0 macrophages (*p* value = 0.001), whereas those in the low MRGPI risk group had more monocytes (*p* value = 0.012) and resting mast cells (*p* value = 0.01) (Figure 4(b)). Furthermore, the linkage pattern among immune cells in HBV-infected patients revealed a link between the immunological milieu and the disease (Figure 4(c)).

### 3.5. Benefits of Immunotherapy in MRGPI Risk Groups

**Figure 5:**
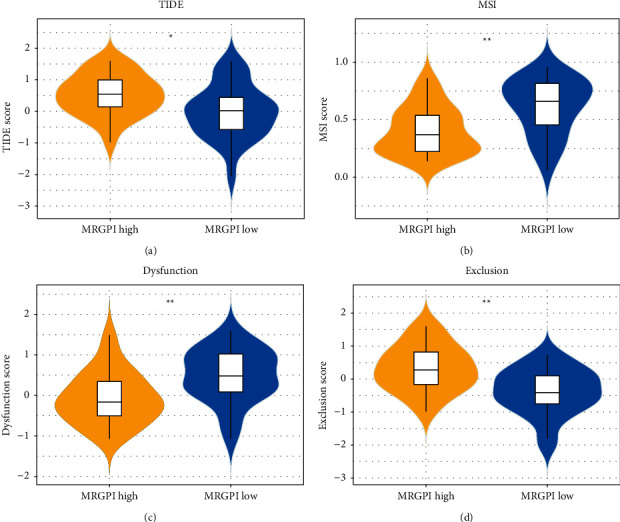
MRGPI association with *T*-cell exclusion and dysfunction. (a–d) TIDE, MSI, and *T*-cell dysfunction and exclusion scores in the MRGPI subgroups (ns: not significant; ^*∗*^*p* < 0.05; ^*∗∗*^*p* < 0.01; ^*∗∗∗*^*p* < 0.001).

TIDE was employed for assessing the prospective clinical efficacy of immunotherapy in the two MRGPI risk groups, with high TIDE prediction scores indicating a high potential for immune evasion and implying that these patients are less likely to gain benefit from immunotherapy. TIDE scores were lower in the low MRGPI risk group (*p* value = 0.015) than those in the high MRGPI risk group (Figure 5(a)), implying that patients in the low MRGPI risk group may have better treatment outcomes. In addition, in the low MRGPI risk group, the MSI (*p* value = 0.015) and *T*-cell dysfunction (*p* value = 0.0026) scores were greater, and the *T*-cell exclusion score (*p* value = 0.0017) was lower (Figures 5(b)–5(d)). The outcome showed that patients in the low MRGPI risk group were more likely to benefit from immunotherapy than those in the high MRGPI risk group.

### 3.6. Differences in Drug Treatment in MRGPI Risk Groups

**Figure 6:**
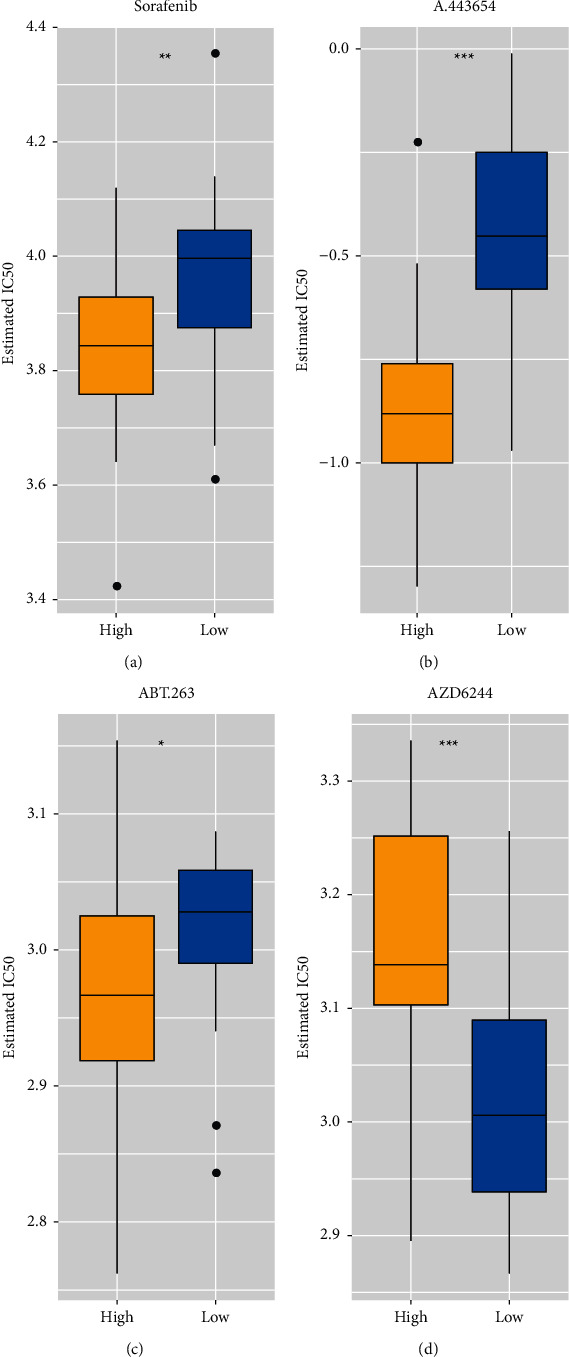
Differences in drug treatment in the MRGPI risk groups. (a–d) Differences in sensitivity of four drugs, including sorafenib, A.443654, ABT.263, and AZD6244, between the MRGPI risk groups in patients with HBV infection (ns: not significant; ^*∗*^*p* < 0.05; ^*∗∗*^*p* < 0.01; ^*∗∗∗*^*p* < 0.001).

The differences in response to drug treatment were considered in patients with HBV infection to assess treatment differences among four drugs, including sorafenib, A.443654, ABT.263, and AZD6244. Hence, the prediction models in the GDSC cell line dataset were trained using ridge regression, and the accuracy of predictions was assessed using 10-fold cross-validation. According to prediction models for these drugs, IC50 values were estimated for each sample in patients with HBV infection. The following significant differences were observed in the IC50 values for all four drugs: Sorafenib (*p* value = 0.0067), A.443654 (*p* value = 5.5*e* − 07), and ABT.263 (*p* value = 0.017) showed a higher sensitivity in the high MRGPI risk group than those in the low MRGPI risk group (Figures 6(a)–6(c)), and AZD6244 (*p* value = 0.00018) showed a higher sensitivity in the low MRGPI risk group than that in the high MRGPI risk group (Figure 6(d)).

## 4. Discussion

Recent evidence shows that metabolic reprogramming may be a hallmark of cancer [22]. Many reports have shown that lipid, glucose, and lactate metabolism have vital effects on TME, angiogenesis, local invasion, and distant metastasis [23–27]. Therefore, WGCNA was performed in the study to cluster MRGs and identify metabolism-related central genes in modules associated with HBV hepatitis. Survival analyses of 248 metabolism-related central genes were performed to construct an MRGPI of ATIC and KIF2C, and the accuracy of the model was validated using the GEO database. Based on different MRGPI scores in the HCC samples, immune cell infiltration and differences in response to immunotherapy were compared. The consistency between different cohorts suggests that the MRGPI has a great prognostic value for patients with HCC, and its components may be essential in the regulation of TME in patients with HBV-infected HCC.

According to many reports, ATIC is linked to various tumor cell proliferation and drug treatment sensitivity [28–30]. It has also been shown that ATIC inhibits autophagy and promotes proliferation, invasion, and metastasis of HCC cells in vivo via the AKT/FOXO3 signaling pathway [31]. Li and other scientists demonstrated that ATIC promotes HCC development by regulating the purine synthesis pathway and inhibits AMPK activation, thereby activating mTOR-S6 K1-S6 signaling to support the growth of HCC cells [32]. The results of the above two experiments are consistent with our findings of ATIC expression being considerably greater in the HCC tissues than that in the normal liver tissues. In addition, some scientists have identified anti-ATIC autoantibodies as a potential HCC-associated serum biomarker [33]. In summary, ATIC is a potential therapeutic and diagnostic target in patients with HCC.

Furthermore, many studies have reported that KIF2C is upregulated in colorectal, breast, and endometrial cancer tissues and is greatly linked to immune infiltration, lymph node metastasis, and OS in cancer patients [34–36]. Some scientists found that KIF2C was highly expressed in HCC, correlating with tumor malignancy. KIF2C is important in HCC progression via the Wnt/*β*-catenin-KIF2C-mTORC1 axis [37]. The results of the above experiments are consistent with our findings that KIF2C negatively affects the prognosis of patients with HBV-infected HCC. However, the mechanism of KIF2C as an MRG affecting HCC development in terms of metabolism remains unclear, warranting further research.

Based on the potential impacts of ATIC and KIF2C on TME, the link between MRGPI and immune cell composition in TME was explored. According to recent research, memory B cells in tertiary lymphoid structures enhance the response to immune checkpoint blockade therapy in metastatic melanoma and metastatic renal cell carcinoma patients, suggesting that memory B cells may potentially contribute to the antitumor response by producing antibodies against the tumor [38]. Since memory B cells independently promote antitumor immune function in immunotherapy, there is a possible basis for patients in the high MRGPI group to receive immune checkpoint blockade therapy. Growing evidence suggests that tumors turn monocytes in TME into a protumor factor, which is confirmed in HCC [39–41]. Resting mast cell infiltration is linked to a poor prognosis in HCC patients [42]. These results suggest that the high MRGPI group has a better immune microenvironment than the low MRGPI group, owing to the rational response of the immune system as the tumor progresses.

TIDE is a computational approach that models two separate mechanisms of tumor immune evasion: dysfunction of tumor-infiltrating cytotoxic *T* lymphocytes (CTLs) and exclusion of CTLs by immunosuppressive factors. TIDE more accurately predicts the prognosis of patients with melanoma treated with ICIs than other biomarkers, such as PD-L1 levels and tumor mutation burden [20]. In the present study, patients in the high MRGPI risk group had high TIDE scores and CTL exclusion levels, and those in the low MRGPI risk group had high CTL dysfunction scores. Accordingly, patients in the low MRGPI risk group have low immune escape levels and may benefit from immunotherapy through CTL activation. MSI is observed in some tumors where the number of repeat units at specific microsatellite loci is altered relative to normal tissues [43]. Research suggests that upon receiving immunotherapy, the prognosis of patients suffering from colorectal cancer with high MSI was improved [44]. In the present study, patients in the low MRGPI risk group had high MSI scores, which is in line with the outcomes of the TIDE analysis. Hence, immunotherapy may be more beneficial to patients in the low MRGPI risk group than those in the high MRGPI risk group.

Finally, differences in response to four drugs, namely, sorafenib, A.443654, ABT.263, and AZD6244, were assessed between the MRGPI risk groups. Patients in the high MRGPI risk group had a higher sensitivity to sorafenib than to the other drugs. A.443654 is a specific inhibitor of Akt and significantly inhibits viral replication in infected or transfected HCC [45]. In the present study, patients in the high MRGPI risk group showed a remarkably higher sensitivity to A.443654 than those in the low MRGPI risk group, suggesting the antiviral effect of A.443654 may be better in the high MRGPI risk group. Some studies reported that combining ABT-263 and sorafenib was safe and efficient in inducing apoptosis in cancer cells in vitro and inhibiting tumor growth and progression in vivo [46]. As with sorafenib, patients in the high MRGPI group showed a higher sensitivity to it. This suggests that sorafenib, in combination with ABT-263, has strong therapeutic potential for patients in the high MRGPI risk group. Unlike the previous three drugs, patients in the low MRGPI risk group were more sensitive to AZD6244 than those in the high MRGPI risk group. Hence, the selection of therapeutic agents based on MRGPI scores can help target the treatment of patients with HCC, reflecting the importance of MRGPI scores in the treatment of HCC.

## 5. Conclusions

An MRGPI, consisting of ATIC and KIF2C, is a promising metabolism-related prognostic biomarker. The MRGPI successfully predicted the prognosis of patients with HBV-infected HCC. Furthermore, the MRGPI grouping may distinguish immunological and molecular features. It may also help determine the efficacy of immunotherapy and conventional drug therapy.

## Figures and Tables

**Table 1:** Clinical information of hepatocellular carcinoma.

Clinical features		Patient
OS	Alive	222
	Dead	123

OS time	≤5 years	302
	>5 years	43

Age	≤60	165
	>60	180

Gender	Female	109
	Male	236

*T*	*T*1	170
	*T*2	85
	*T*3	74
	*T*4	13
	Missing value	3

*N*	*N*0	241
	*N*1	3
	Missing value	101

*M*	*M*0	246
	*M*1	3
	Missing value	96

Stage	I	163
	II	78
	III	80
	IV	3
	Missing value	21

Grade	*G*1	53
	*G*2	162
	*G*3	113
	*G*4	12
	Missing value	5

Hepatitis	HBV	53
	HCV	18
	Missing value	274

Total		345

## Data Availability

The data used to support the findings of this study are available from the corresponding author on reasonable request.

## Supplementary Materials

Supplementary Materials
*Supplementary Figure 1.* Identification of coexpressed metabolic genes associated with HBV hepatitis using WGCNA. (a) Soft threshold selection in WGCNA network analysis. (b) Gene distribution in WGCNA network analysis. (c) Heatmap of topological overlap in WGCNA network analysis. *Supplementary Figure 2.* Validation of MRGPI models in other datasets. (a–c) Survival curves of ATIC, KIF2C, and POLR3C genes in the GSE14520 validation set. (d, e) Survival and ROC curves of the prognostic model in the GSE14520 validation set. *Supplementary Table 1.* Genes included in each module of WGCNA analysis. *Supplementary Table 2.* Differentially expressed genes in the high and low MRGPI risk groups.
