# Supplementary material for: A Novel Metabolism-Related Gene Signature for Predicting the Prognosis of HBV-Infected Hepatocellular Carcinoma
Source: J Oncol. 2022 Aug 28;2022:2391265. doi: 10.1155/2022/2391265 (PMC9441393; doi:10.1155/2022/2391265)
Supplement: Supplementary Materials — Supplementary Figure 1. Identification of coexpressed metabolic genes associated with HBV hepatitis using WGCNA. (a) Soft threshold selection in WGCNA network analysis. (b) Gene distribution in WGCNA network analysis. (c) Heatmap of topological overlap in WGCNA network analysis. Supplementary Figure 2. Validation of MRGPI models in other datasets. (a–c) Survival curves of ATIC, KIF2C, and POLR3C genes in the GSE14520 validation set. (d, e) Survival and ROC curves of the prognostic model in the GSE14520 validation set. Supplementary Table 1. Genes included in each module of WGCNA analysis. Supplementary Table 2. Differentially expressed genes in the high and low MRGPI risk groups. [file 2391265.f1.zip › Supplementary+Material+.docx]

**Supplementary Figure 1: Identification of co-expressed metabolic genes associated with HBV hepatitis using WGCNA**

**(A)** Soft threshold selection in WGCNA network analysis. **(B)** Gene distribution in WGCNA network analysis. **(C)** Heatmap of topological overlap in WGCNA network analysis.


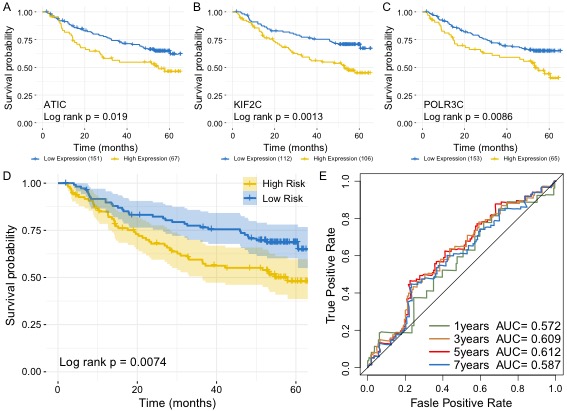


**Supplementary Figure 2: Validation of MRGPI models in other datasets**

**(A–C)** Survival curves of ATIC, KIF2C, and POLR3C genes in the GSE14520 validation set. **(D–E)** Survival and ROC curves of a prognostic model in the GSE14520 validation set.

**Supplementary Table 1: Genes included in each module of WGCNA analysis**

Gene Module

CYP1A2 grey

ACSM3 grey

ACADS grey

ECHS1 grey

HADH grey

ACADM grey

MECR grey

HADHA grey

HADHB grey

ACADL grey

ACADVL grey

ALOX12 blue

ALOX5 grey

CYP2C8 grey

CYP2C9 grey

CYP2D6 grey

CYP2E1 grey

CYP3A4 grey

ACSM2B grey

ACSM1 grey

GLYAT grey

CYP8B1 grey

CYP46A1 grey

CYP39A1 grey

HSD3B7 grey

AKR1D1 grey

AKR1C4 grey

CYP27A1 grey

SLC27A5 grey

SLC27A2 grey

CYP7B1 grey

RXRA grey

NR1H4 grey

NCOA1 blue

NCOA2 grey

CYP7A1 grey

AMACR grey

ACOX2 grey

HSD17B4 grey

SCP2 grey

ACOT8 grey

BAAT grey

ABCB11 grey

PLA2G6 grey

TAZ grey

LCLAT1 blue

PLA2G4A brown

PNPLA2 grey

PNPLA3 grey

DGAT2L6 grey

DGAT1 grey

DGAT2 grey

AWAT2 grey

MGLL grey

PLA2G4B grey

PLA2G4D grey

PLA2G4F grey

PLBD1 yellow

PLA2G4C grey

PNPLA8 blue

PLA2G2A grey

LPCAT1 grey

LPCAT2 grey

LPCAT3 grey

LPCAT4 brown

MBOAT2 brown

PLA2G4E grey

PLA2G1B grey

PLA2G2D green

PLA2G2E grey

PLA2G2F grey

PLA2G3 brown

PLA2G5 grey

PLA2G10 grey

PLA2G12A grey

PLA2R1 brown

PLB1 green

MBOAT1 grey

LPGAT1 grey

CRLS1 grey

MBOAT7 blue

PLA1A grey

SLC25A17 blue

PECR grey

PHYH grey

ALDH3A2 grey

CYP17A1 grey

POMC grey

HSD3B1 grey

HSD3B2 grey

HSD17B3 grey

CGA grey

LHB grey

SRD5A1 grey

SRD5A2 grey

SRD5A3 grey

ACOXL grey

ACOX3 grey

CROT grey

CRAT grey

ACOX1 grey

EHHADH grey

ACAA1 grey

MLYCD grey

DECR2 grey

ACOT4 grey

ACOT6 grey

MAOB grey

MAOA grey

GPX4 grey

HPGD grey

CYP1A1 grey

ALOX15 grey

SC5D grey

DHCR7 grey

DHCR24 grey

PANK2 blue

PANK1 grey

PPCS blue

PPCDC grey

COASY grey

CYP4F2 grey

CYP4F3 grey

CYP4F8 grey

TBXAS1 green

PTGIS brown

CYP4F22 grey

CYP1B1 grey

AHR grey

ARNT blue

ARNT2 blue

CYP11A1 grey

FDXR grey

FDX1 grey

CYP11B1 grey

CYP11B2 grey

CYP19A1 grey

CYP21A2 grey

CYP51A1 grey

CYP4V2 grey

HSD17B1 grey

HSD17B11 grey

AKR1B15 grey

HSD17B14 grey

HSD17B2 grey

CYP2J2 grey

CYP4A11 grey

CYP4B1 grey

CYP4F12 grey

UGP2 grey

UGDH grey

HSD11B1 grey

HSD11B2 grey

OPLAH grey

GCLC grey

GCLM grey

GGCT grey

GSS grey

GGT1 grey

CNDP2 grey

GDE1 blue

GDPD1 grey

GDPD3 grey

PNPLA6 grey

PNPLA7 grey

GDPD5 grey

NDST1 grey

NDST2 blue

HGSNAT blue

PLA2G15 brown

GPCPD1 grey

ST3GAL1 grey

ST3GAL2 blue

ST3GAL3 grey

ST3GAL4 grey

ST3GAL6 grey

ACSL1 grey

FADS2 grey

ELOVL1 blue

ELOVL2 grey

ELOVL3 grey

ELOVL5 grey

FADS1 grey

ABCD1 grey

CYP2S1 grey

CYP2U1 grey

CYP2W1 grey

CYP3A43 grey

CYP4F11 grey

PARP16 grey

PARP10 grey

PARP9 grey

NAMPT grey

SLC22A13 grey

SLC5A8 grey

NAPRT grey

NNMT grey

PTGS2 grey

NUDT12 grey

RNLS grey

SMOX brown

PAOX grey

NUDT1 yellow

NUDT15 grey

NUDT18 grey

NUDT16 grey

NUDT5 grey

NUDT9 grey

ADPRM grey

AKR1B1 brown

PCCA grey

PCCB grey

MCEE grey

MMAA grey

ABCC3 grey

SLC10A1 grey

SLCO1A2 grey

SLCO1B1 grey

SLCO1B3 grey

PDK1 grey

PDK2 grey

PDK3 blue

PDK4 grey

PPARD blue

PDP1 blue

PDPR blue

PDP2 grey

DIO1 grey

DIO2 grey

DIO3 grey

ETHE1 grey

TSTD1 grey

CYP2C19 grey

GPX1 grey

ALOX5AP green

LTC4S grey

MAPKAPK2 grey

LTA4H grey

PTGR1 grey

GGT5 grey

DPEP1 grey

GPD2 blue

GPD1 grey

GPD1L blue

GNPAT grey

AGPAT1 grey

AGPAT2 grey

AGPAT3 grey

AGPAT4 brown

AGPAT5 grey

GPAM grey

GPAT2 grey

LIPH grey

LIPI grey

PLD6 grey

PLD1 blue

PLD2 blue

DDHD1 grey

DDHD2 blue

ACP6 grey

ALPI grey

PEMT grey

CSNK2B yellow

CSNK2A1 blue

CSNK2A2 blue

CHKA grey

CHKB grey

PHOSPHO1 green

CHAT brown

PCYT1A blue

PCYT1B grey

LPIN1 grey

LPIN2 grey

LPIN3 grey

CEPT1 blue

CHPT1 grey

ETNK1 blue

ETNK2 grey

ETNPPL grey

PCYT2 grey

PISD blue

CDS2 grey

PGS1 blue

PTPMT1 grey

CDS1 grey

CDIPT grey

PI4KA blue

PI4K2B grey

SACM1L blue

MTMR2 blue

SBF1 blue

PI4K2A grey

PI4KB blue

ARF1 grey

ARF3 blue

PIK3C3 blue

PIK3R4 blue

PIK3C2A blue

FIG4 blue

VAC14 grey

PIKFYVE blue

PIK3C2G grey

TPTE2 grey

OCRL blue

INPP5E blue

INPP4A blue

INPP4B green

INPP5F blue

MTM1 grey

MTMR4 grey

MTMR12 blue

MTMR7 grey

PIP5K1A blue

PIP5K1B yellow

PIP5K1C blue

SYNJ1 blue

SYNJ2 blue

INPP5K grey

INPP5J grey

PIK3CA blue

PIK3R1 grey

PIK3R2 yellow

PIK3R3 brown

PIK3CB blue

PIK3CD green

PIK3CG green

PIK3R5 green

PIK3R6 green

PTEN grey

INPP5D green

INPPL1 blue

PIK3C2B blue

PIP4K2A green

PIP4K2B blue

MTMR1 blue

MTMR3 blue

MTMR6 blue

MTMR14 grey

MTMR9 blue

MTMR8 grey

PIP4K2C blue

SBF2 blue

BMX grey

PTGS1 grey

AKR1C3 grey

CBR1 grey

PTGDS grey

HPGDS grey

PTGR2 grey

CH25H grey

ACSBG1 grey

ACSBG2 grey

ACSF3 grey

SLC27A3 grey

ACSL3 grey

ACSL5 grey

ACSL6 grey

ACSL4 grey

ELOVL6 grey

ELOVL7 grey

ELOVL4 brown

HSD17B12 grey

TECR grey

CYP2R1 grey

CYP24A1 grey

CYP26A1 grey

CYP26B1 grey

CYP26C1 grey

CYP27B1 grey

DECR1 grey

ECI1 grey

PRPS1 grey

NAT2 grey

PRKCA blue

AZIN2 brown

AGMAT grey

AWAT1 grey

FAAH grey

FAAH2 grey

HLCS blue

BTD grey

TMLHE grey

SHMT1 grey

ALDH9A1 grey

BBOX1 grey

TH yellow

DDC grey

DBH grey

PNMT grey

ACAT2 grey

HMGCS1 grey

HMGCR grey

MVK grey

PMVK grey

MVD grey

IDI1 grey

IDI2 grey

FDPS grey

GGPS1 blue

FDFT1 grey

SQLE grey

LSS grey

LBR blue

TM7SF2 grey

MSMO1 grey

NSDHL grey

HSD17B7 grey

CS blue

ACO2 grey

IDH3A blue

IDH3B grey

IDH3G grey

IDH2 grey

NNT grey

OGDH grey

DLST grey

DLD grey

SUCLG1 grey

SUCLG2 grey

SUCLA2 blue

SDHA grey

SDHB grey

SDHC grey

SDHD grey

FH grey

MDH2 grey

FAHD1 grey

ME2 blue

ME3 grey

ABCD4 grey

MMACHC grey

MTRR blue

MTR grey

ABCC1 blue

GATM grey

GAMT grey

CKB grey

CKM yellow

CKMT2 grey

CKMT1A grey

CBS grey

CTH grey

POR grey

PAPSS1 blue

PAPSS2 grey

ABHD14B grey

BPNT1 grey

IMPAD1 grey

CSAD grey

ADO blue

GADL1 grey

GOT2 grey

MPST grey

ADH1A grey

ADH1B grey

ADH1C grey

ADH4 grey

ADH6 grey

ADH7 grey

ALDH2 grey

ALDH1B1 grey

ACSS1 grey

ALDH1A1 grey

ACSS2 grey

ADH5 grey

FMO1 grey

FMO2 grey

FMO3 grey

ACLY blue

MORC2 blue

ACACA grey

FASN grey

SCD grey

SCD5 grey

PPT2 yellow

CBR4 grey

HSD17B8 grey

MT-ATP6 grey

MT-ATP8 grey

SORD grey

KHK grey

ALDOB grey

GLYCTK grey

GNAS grey

RAPGEF3 grey

RAPGEF4 grey

PC grey

MDH1 grey

GOT1 grey

PCK1 grey

PCK2 grey

ENO1 grey

ENO2 grey

ENO3 grey

PGAM1 grey

PGK1 grey

GAPDH grey

GAPDHS grey

TPI1 grey

FBP1 grey

FBP2 grey

GPI grey

G6PC grey

G6PC2 grey

G6PC3 blue

UGT1A1 grey

UGT1A6 grey

UGT1A4 grey

UGT1A10 grey

ESD grey

AKR1A1 grey

AGK blue

GLDC grey

AMT grey

PHKA1 grey

PHKB grey

PHKG1 grey

CALM1 grey

PHKA2 grey

PHKG2 grey

PYGM grey

PYGL grey

AGL grey

PGM1 grey

PGM2 grey

PGM2L1 brown

GAA grey

GYG1 blue

GYS1 blue

GYG2 grey

GYS2 grey

PPP1R3C grey

EPM2A grey

GCK grey

HK1 brown

HK2 grey

HK3 green

ADPGK brown

GNPDA1 brown

GNPDA2 blue

PPP2R5D blue

PRKACA grey

PRKACB blue

PRKACG grey

PFKFB1 grey

PFKFB2 grey

PFKFB3 blue

PFKFB4 grey

PFKL grey

PFKM grey

PFKP grey

PGP yellow

PKM brown

PKLR grey

HEXA grey

HEXB grey

SUMF1 grey

SUMF2 blue

GALC grey

SMPD1 grey

GBA grey

PSAP brown

GBA2 grey

UGT8 grey

CERK grey

HAL grey

UROC1 grey

AMDHD1 grey

FTCD grey

HDC grey

HNMT grey

CARNS1 grey

CPT1A grey

CPT1B grey

CPT2 grey

MID1IP1 grey

THRSP grey

ACACB grey

PRKAA2 grey

PRKAB2 grey

PRKAG2 grey

L2HGDH blue

D2HGDH grey

ADHFE1 grey

SAT1 grey

AASS grey

CRYM grey

PIPOX grey

HYKK grey

PHYKPL grey

ALDH7A1 grey

AADAT grey

GCDH grey

DHFR grey

FPGS grey

SHMT2 grey

MTHFD1L blue

MTHFD1 grey

MTHFR grey

ALDH1L1 grey

MTHFD2 grey

MTHFD2L grey

ALDH1L2 brown

MTHFS grey

TXNRD1 grey

GSR grey

MAT1A grey

GNMT grey

AHCY grey

SCLY grey

UBIAD1 grey

MTAP blue

APIP grey

ENOPH1 blue

ADI1 grey

MAT2B grey

MAT2A blue

TPMT grey

COMT grey

AS3MT grey

GSTO1 grey

N6AMT1 grey

TRMT112 yellow

INMT grey

ACAD10 grey

ACAD11 grey

ACAA2 grey

MCAT grey

ACSF2 grey

ACOT2 grey

ACOT9 brown

THEM5 grey

THEM4 grey

ACOT1 grey

ACOT11 grey

ACOT12 grey

ACOT13 grey

ACOT7 grey

PCTP grey

MOCS1 grey

NFS1 grey

MOCS3 blue

MOCS2 grey

GPHN grey

MOCOS grey

IDH1 grey

QPRT grey

NMRK1 grey

NMRK2 grey

NMNAT2 grey

NADSYN1 grey

NADK grey

NMNAT1 grey

NMNAT3 grey

NT5E grey

BST1 grey

CD38 green

NADK2 grey

ADCY2 grey

ADCY3 brown

ADCY4 brown

ADCY6 blue

ADCY7 green

ADCY9 grey

ADCY1 grey

ADCY5 grey

ADCY8 grey

HAO2 grey

NUDT7 grey

ASRGL1 grey

PAH grey

PCBD1 grey

QDPR grey

TAT grey

HPD grey

HGD grey

GSTZ1 grey

FAH grey

IL4I1 grey

ENTPD1 blue

ENTPD2 grey

ENTPD3 grey

ENTPD4 blue

ENTPD5 grey

ENTPD6 grey

ENTPD8 grey

PRODH grey

PRODH2 grey

ALDH4A1 grey

NT5C2 blue

NT5C1B blue

NT5C1A grey

NT5C yellow

DNPH1 grey

PNP grey

GDA grey

XDH grey

ITPA yellow

PPAT blue

LHPP grey

GART blue

PFAS blue

PAICS blue

ADSL blue

ATIC blue

IMPDH1 brown

IMPDH2 yellow

GMPS blue

ADSSL1 grey

ADSS blue

DCK blue

APRT yellow

AMPD1 green

AMPD2 grey

AMPD3 green

HPRT1 grey

DGUOK yellow

ADAL grey

ADK grey

ADA green

GMPR grey

GMPR2 grey

CAD blue

DHODH grey

UMPS grey

NT5M grey

NT5C3A grey

UPP1 brown

UPP2 grey

TYMP grey

DPYD grey

DPYS grey

UPB1 grey

AGXT2 grey

TK2 grey

TK1 blue

UCKL1 blue

UCK1 grey

UCK2 blue

CDA grey

ME1 grey

LDHA grey

LDHB grey

LDHC grey

LDHAL6B grey

PDHB grey

PDHA1 grey

PDHA2 yellow

DLAT grey

PDHX grey

HAGH grey

NDUFV3 grey

NDUFA12 grey

NDUFS4 grey

NDUFS6 yellow

NDUFS1 blue

NDUFV1 grey

NDUFV2 grey

NDUFS5 grey

MT-ND4 grey

MT-ND5 grey

MT-ND2 grey

MT-ND3 grey

MT-ND6 grey

NDUFB6 grey

MT-ND1 grey

NDUFA1 grey

NDUFA2 grey

NDUFA3 yellow

NDUFA6 grey

NDUFA7 yellow

NDUFA8 grey

NDUFAB1 grey

NDUFB2 grey

NDUFB3 grey

NDUFB4 grey

NDUFB5 grey

NDUFB7 grey

NDUFB8 grey

NDUFB9 yellow

NDUFB10 grey

NDUFC1 grey

NDUFC2 grey

NDUFA13 yellow

NDUFA11 yellow

NDUFB11 yellow

NDUFA10 grey

NDUFB1 grey

NDUFA5 grey

NDUFA9 grey

NDUFS2 grey

NDUFS3 grey

NDUFS7 grey

NDUFS8 grey

COQ10A grey

COQ10B grey

TRAP1 grey

ETFA grey

ETFB grey

ETFDH grey

COX8A grey

COX7B grey

COX7C grey

COX6A1 grey

COX6B1 yellow

COX6C grey

COX5A grey

COX5B grey

MT-CO1 grey

MT-CO2 grey

MT-CO3 grey

COX4I1 grey

COX7A2L grey

NDUFA4 grey

COX19 grey

COX11 blue

COX14 grey

COX16 grey

COX20 grey

COX18 grey

LRPPRC blue

SCO1 grey

SCO2 grey

SURF1 grey

TACO1 grey

CLPS yellow

PNLIP grey

RDH11 grey

AKR1B10 grey

AKR1C1 grey

RETSAT grey

DARS blue

EEF1E1 yellow

EPRS blue

IARS blue

AIMP2 grey

KARS grey

LARS blue

MARS blue

QARS yellow

AIMP1 grey

RARS grey

SARS grey

PSTK grey

SEPHS2 grey

RPS10 yellow

RPS11 yellow

RPS12 yellow

RPS13 yellow

RPS14 yellow

RPS15 yellow

RPS16 yellow

RPS17 yellow

RPS18 yellow

RPS19 yellow

RPS15A yellow

RPS2 yellow

RPS20 yellow

RPS21 yellow

RPS23 yellow

RPS24 yellow

RPS25 yellow

RPS26 yellow

RPS27A yellow

RPS28 yellow

RPS29 yellow

RPS3 yellow

FAU yellow

RPS3A yellow

RPS5 yellow

RPS6 yellow

RPS7 yellow

RPS8 yellow

RPS9 yellow

RPSA yellow

RPS27 yellow

RPS4X yellow

RPS4Y1 grey

RPLP0 yellow

RPLP1 yellow

RPLP2 yellow

RPL10A yellow

RPL11 yellow

RPL12 yellow

RPL13 yellow

RPL13A yellow

RPL14 yellow

RPL15 yellow

RPL17 yellow

RPL18 yellow

RPL18A yellow

RPL19 yellow

RPL21 yellow

RPL23 yellow

RPL23A yellow

RPL24 yellow

RPL27 yellow

RPL27A yellow

RPL28 yellow

RPL29 yellow

RPL30 yellow

RPL31 yellow

RPL32 yellow

RPL34 yellow

RPL35 yellow

RPL35A yellow

RPL36 yellow

RPL37 yellow

RPL37A yellow

RPL38 yellow

RPL4 yellow

UBA52 yellow

RPL41 yellow

RPL5 yellow

RPL6 yellow

RPL7 yellow

RPL7A yellow

RPL8 yellow

RPL9 yellow

RPL10 yellow

RPL22 yellow

RPL26 yellow

RPL3 yellow

RPL36A yellow

RPL39 yellow

SECISBP2 blue

EEFSEC grey

PHGDH grey

PSAT1 grey

PSPH blue

SRR grey

TPH1 grey

TPH2 grey

AANAT grey

ASMT grey

KDSR blue

PRKD1 grey

PRKD2 blue

PRKD3 blue

DEGS1 blue

FA2H grey

DEGS2 grey

CSNK1G2 blue

PPM1L grey

VAPA blue

VAPB blue

COL4A3BP blue

SPTLC1 blue

SPTLC2 blue

SPTSSA grey

SPTSSB grey

SPTLC3 grey

ORMDL1 blue

ORMDL2 grey

ORMDL3 grey

SGMS2 blue

SPHK1 grey

SPHK2 grey

CERS1 grey

CERS2 blue

CERS3 grey

CERS4 grey

CERS5 blue

CERS6 blue

SGPP1 blue

SGPP2 blue

ALDH3B1 grey

ALDH3B2 grey

BDH2 grey

AACS blue

ACSS3 grey

ACAT1 grey

HMGCS2 grey

HMGCL grey

HMGCLL1 grey

BDH1 grey

GCH1 grey

GCHFR grey

PRKG2 grey

SPR grey

DUOX1 grey

DUOX2 grey

TPO grey

IYD grey

TSHB grey

AGMO grey

GK grey

MOGAT1 grey

MOGAT2 grey

MOGAT3 grey

CAV1 brown

FABP4 grey

LIPE grey

PPP1CA grey

PPP1CB blue

PPP1CC blue

PNPLA4 grey

PLIN3 brown

PNPLA5 grey

TDO2 grey

IDO1 green

IDO2 grey

AFMID grey

KMO grey

KYNU grey

HAAO grey

ACMSD grey

PDSS1 blue

PDSS2 grey

COQ2 grey

COQ6 grey

COQ3 grey

COQ5 grey

COQ9 grey

COQ7 grey

NAGS grey

CPS1 grey

OTC grey

ASS1 grey

NMRAL1 grey

ASL grey

ARG1 grey

ARG2 grey

OXCT1 grey

OXCT2 grey

TPK1 grey

THTPA blue

RFK blue

FLAD1 grey

ENPP1 grey

ACP5 grey

VNN1 grey

VNN2 grey

CYB5R3 grey

GSTO2 grey

PDXK grey

PNPO grey

AOX1 grey

HSP90AA1 grey

AKT1 blue

NOS3 grey

DDAH1 grey

DDAH2 grey

LYPLA1 grey

CYGB grey

STK11 blue

ADIPOR1 grey

ADIPOR2 grey

ADIPOQ grey

GUK1 grey

ABCG2 grey

ABCB1 grey

CYP3A5 grey

ACY1 grey

ACY3 grey

AKR7A2 grey

AKR7A3 grey

GPT grey

GPT2 grey

OAT grey

ALDH18A1 grey

PYCR1 grey

PYCR2 grey

ASNS brown

ASPG grey

GLUD1 grey

GLUL grey

GLS blue

GLS2 grey

NAT8L grey

ASPA grey

FOLH1 grey

NAALAD2 grey

RIMKLA grey

RIMKLB brown

BCAT1 green

BCAT2 grey

BCKDHA grey

BCKDHB grey

DBT grey

BCKDK grey

PPM1K grey

IVD grey

MCCC2 grey

MCCC1 grey

AUH grey

ACADSB grey

HSD17B10 grey

ACAD8 grey

HIBCH grey

HIBADH grey

ALDH6A1 grey

CHDH grey

SARDH grey

CRYL1 grey

DCXR grey

XYLB grey

GALK1 grey

GALT grey

GALE grey

GCG grey

GCGR grey

HOGA1 grey

DAO grey

DDO grey

AGXT grey

GRHPR grey

HAO1 grey

ALAS1 grey

ALAS2 grey

ALAD grey

HMBS grey

UROS grey

UROD grey

CPOX blue

PPOX grey

FECH grey

COX10 grey

HMOX1 grey

HMOX2 grey

BLVRB grey

BLVRA grey

TALDO1 grey

TKT grey

AK1 grey

AK2 grey

AK5 green

AK7 grey

AK8 grey

AK9 grey

AK4 grey

CMPK1 blue

DTYMK yellow

CTPS1 grey

CTPS2 blue

RRM1 blue

RRM2 blue

RRM2B grey

GLRX grey

NME1 yellow

NME2 grey

NME4 grey

DCTPP1 yellow

NUDT13 grey

NME3 grey

DCTD blue

DUT grey

TYMS blue

TYR grey

OCA2 grey

SLC45A2 grey

TYRP1 grey

ODC1 grey

AMD1 blue

SRM yellow

SMS brown

G6PD grey

PGLS yellow

PGD grey

RBKS grey

RPIA blue

RPE blue

RPEL1 grey

DERA grey

SHPK grey

ALDH3A1 grey

CES1 grey

CES2 grey

AOC1 grey

AOC2 grey

AOC3 grey

BPHL grey

1-Mar grey

2-Mar grey

CYB5B grey

CBR3 grey

CES3 grey

AGPS blue

KCNJ11 yellow

ABCC8 yellow

VAMP2 grey

STX1A brown

BHMT2 grey

INPP5A blue

INPP5B blue

INPP1 grey

IMPA1 grey

IMPA2 grey

ISYNA1 grey

MIOX grey

PLCD4 grey

PLD4 green

ITPKA grey

ITPKB brown

ITPKC grey

ITPK1 grey

MINPP1 grey

IPMK blue

IPPK blue

IP6K1 blue

IP6K2 blue

IP6K3 grey

NUDT4 grey

NUDT3 blue

NUDT10 brown

NUDT11 brown

PPIP5K1 grey

PPIP5K2 blue

GCAT grey

SDS grey

SDSL grey

FAR1 blue

FAR2 grey

GALM grey

HKDC1 grey

BPGM blue

PGAM4 grey

ALDH1A3 grey

LDHAL6A grey

OGDHL grey

ACO1 grey

IDNK grey

TKTL1 grey

TKTL2 grey

PRPS2 grey

PRPS1L1 grey

GUSB grey

KL grey

UGT2B4 grey

UGT2B7 grey

UGT2B10 grey

UGT2B15 grey

UGT2B17 grey

UGT2B11 grey

UGT2A1 grey

UGT2B28 grey

UGT1A8 grey

UGT1A7 grey

UGT1A5 grey

UGT1A9 grey

UGT1A3 grey

UGT2A3 grey

UGT2A2 grey

DHDH grey

MPI grey

PMM1 grey

PMM2 blue

GMDS grey

TSTA3 grey

FPGT blue

ALDOA grey

ALDOC grey

GLA grey

GLB1 grey

LCT grey

OXSM grey

OLAH grey

PPT1 brown

CPT1C grey

EBP grey

NQO1 grey

AKR1C2 grey

SULT2B1 grey

CYP3A7 grey

HSD17B6 grey

ATP6V1A blue

ATP6V1B1 grey

ATP6V1B2 blue

ATP6V0C blue

ATP6V1C1 grey

ATP6V1E1 brown

ATP6V0B grey

ATP6V1G2 grey

ATP6V0A1 grey

ATP6AP1 grey

ATP6V0E1 grey

ATP6V0D1 grey

ATP6V1F brown

ATP6V1G1 grey

TCIRG1 grey

ATP6V0A2 blue

ATP6V0A4 grey

ATP6V1D grey

ATP6V1H grey

ATP6V1E2 blue

ATP6V1G3 grey

ATP6V0E2 grey

ATP6V0D2 grey

ATP6V1C2 grey

ATP12A grey

ATP4A grey

ATP4B grey

ABHD14A-ACY1 grey

NME6 blue

NME7 blue

NME1-NME2 grey

NT5C3B yellow

NT5C1B-RDH14 grey

NTPCR grey

PDE1A brown

PDE1C grey

PDE2A grey

PDE3A grey

PDE3B grey

PDE1B green

PDE10A grey

PDE11A grey

NUDT2 grey

POLA1 blue

POLD1 blue

POLD2 grey

POLE blue

POLE2 blue

PRIM1 blue

PRIM2 blue

POLD3 blue

POLA2 blue

POLE3 blue

POLE4 grey

POLD4 grey

POLR3D yellow

POLR2A blue

POLR2B blue

POLR2C grey

POLR2D blue

POLR2E grey

POLR2F grey

POLR2G yellow

POLR2H yellow

POLR2I yellow

POLR2J yellow

POLR2K grey

POLR2L yellow

POLR1C yellow

POLR3F blue

POLR3G grey

POLR3C blue

POLR3A blue

POLR1A blue

ZNRD1 yellow

POLR1D grey

POLR3K grey

POLR3B blue

POLR3E blue

POLR1E grey

POLR1B blue

POLR3GL grey

POLR3H grey

TWISTNB blue

POLR2J2 grey

POLR2J3 grey

ADCY10 grey

GUCY1A2 brown

GUCY2C grey

GUCY2F grey

GUCY2D grey

NPR1 grey

NPR2 grey

PNPT1 blue

HDDC3 grey

PDE4A grey

PDE4B green

PDE4C grey

PDE4D grey

PDE7A blue

PDE8A blue

PDE8B grey

PDE7B grey

AK3 brown

ENPP3 grey

NAT1 grey

CYP2A6 grey

UPRT blue

CANT1 blue

CMPK2 grey

ALDH5A1 grey

GFPT1 blue

GFPT2 brown

ABAT grey

NIT2 grey

BHMT grey

DMGDH grey

TST grey

AHCYL1 blue

AHCYL2 blue

MRI1 blue

MUT grey

EZH1 blue

EZH2 blue

KMT2A blue

SETMAR grey

SUV39H1 grey

PRDM2 blue

KMT2D blue

SETD1A blue

KMT2B grey

SETDB1 blue

EHMT2 blue

PRDM7 grey

SETD1B blue

SETD2 blue

ASH1L blue

KMT2E blue

PRDM9 grey

KMT2C blue

NSD1 blue

SUV39H2 blue

EHMT1 blue

SETD7 grey

SETDB2 grey

SETD3 blue

DOT1L blue

PRDM6 brown

CAMKMT grey

PLOD3 grey

COLGALT2 grey

COLGALT1 brown

SAT2 grey

CNDP1 grey

P4HA1 grey

P4HA2 grey

P4HA3 grey

L3HYPDH grey

MIF grey

GAD1 grey

GAD2 yellow

MARS2 grey

TXNRD2 grey

TXNRD3 grey

SEPSECS grey

ANPEP grey

GGT7 grey

GGT6 grey

GSTA1 grey

GSTA2 grey

GSTA3 grey

GSTA4 grey

GSTM1 grey

GSTM2 grey

GSTM3 grey

GSTM4 grey

GSTM5 grey

GSTP1 grey

MGST1 grey

MGST2 grey

MGST3 grey

GSTA5 grey

GSTK1 grey

GSTT2B grey

LAP3 grey

GBE1 grey

GANC grey

MGAM grey

SI grey

AMY1A grey

AMY1B grey

AMY1C grey

AMY2A grey

AMY2B grey

DOLPP1 grey

ALG5 grey

DPAGT1 blue

ALG3 grey

ALG6 blue

ALG1 grey

ALG2 blue

ALG11 blue

ALG12 blue

ALG10 blue

ALG10B blue

MOGS blue

GANAB blue

MGAT1 grey

MAN2A2 blue

MAN2A1 blue

MGAT2 blue

MGAT3 grey

FUT8 blue

B4GALT1 grey

B4GALT3 blue

B4GALT2 grey

MGAT5 blue

MGAT5B brown

MAN1A1 grey

MAN1A2 blue

MAN1B1 yellow

MAN1C1 grey

ST6GAL1 grey

ST6GAL2 grey

ST6GALNAC1 grey

B3GNT6 grey

GCNT1 grey

GCNT3 grey

GCNT4 grey

POMT1 grey

POMT2 blue

POMGNT1 grey

B3GAT1 grey

B3GAT2 grey

POMGNT2 blue

B3GALNT2 blue

POMK blue

FKTN blue

FKRP blue

B4GAT1 grey

ISPD grey

CHIT1 green

CHIA grey

NAGK brown

PGM3 grey

UAP1 grey

UAP1L1 grey

RENBP grey

NANS yellow

GNPNAT1 grey

AMDHD2 grey

UXS1 blue

IDS grey

IDUA grey

ARSB grey

HPSE green

HPSE2 yellow

SGSH blue

GNS blue

GALNS brown

HYAL1 grey

SPAM1 grey

HYAL3 grey

HYAL2 grey

HYAL4 grey

XYLT1 brown

XYLT2 blue

B4GALT7 grey

B3GAT3 grey

CSGALNACT2 blue

CSGALNACT1 grey

CHSY1 brown

CHPF2 blue

CHPF grey

CHSY3 brown

GK2 grey

CEL grey

LIPC grey

PNLIPRP1 yellow

LIPF grey

LIPG grey

PNLIPRP3 grey

PIP5KL1 grey

PIGL grey

PIGM blue

PIGX blue

PIGN blue

PIGB blue

PIGF blue

PIGS blue

PIGT blue

PIGU blue

PIGA blue

PIGC blue

PIGH grey

PIGP grey

PIGQ grey

DPM2 yellow

PLD3 grey

PTDSS1 grey

PTDSS2 yellow

PLA2G16 grey

LCAT grey

JMJD7-PLA2G4B blue

PLA2G12B grey

PLA2G2C grey

LYPLA2 grey

PAFAH1B1 blue

PAFAH1B2 blue

PAFAH1B3 yellow

PAFAH2 grey

PLA2G7 grey

TMEM86B grey

ENPP2 grey

PTGES grey

PTGES3 blue

PTGES2 yellow

GPX2 grey

GPX3 grey

GPX5 grey

GPX7 grey

GPX6 grey

GPX8 brown

CYP2B6 grey

ASAH1 grey

ASAH2 grey

SMPD2 grey

SMPD3 grey

SMPD4 blue

ENPP7 grey

ACER1 grey

ACER2 blue

GAL3ST1 grey

NEU1 grey

NEU2 grey

NEU3 grey

NEU4 grey

B4GALT6 grey

B3GNT5 grey

B4GALT4 blue

FUT3 grey

FUT5 grey

FUT6 grey

FUT9 grey

FUT4 blue

FUT1 grey

FUT2 grey

B3GNT2 blue

B3GNT3 grey

B3GNT4 grey

ST8SIA1 green

FUT7 grey

ABO grey

GCNT2 grey

B3GALT1 grey

B3GALT2 grey

B3GALT5 grey

A4GALT brown

NAGA grey

A3GALT2 grey

B3GALNT1 blue

ST3GAL5 grey

B4GALNT1 grey

B3GALT4 grey

ST8SIA5 grey

ST6GALNAC3 brown

ST6GALNAC4 grey

GLO1 grey

LDHD grey

HYI grey

CAT grey

GCSH grey

ECHDC1 grey

ST20-MTHFS grey

PDXP grey

PHOSPHO2 grey

PANK4 blue

PANK3 blue

LIAS grey

LIPT2 grey

DHRS4 grey

DHRS4L2 grey

RDH5 grey

RDH12 grey

LRAT grey

RPE65 grey

BCO1 grey

ALDH1A2 grey

SDR16C5 grey

COX15 blue

HCCS grey

CP grey

FXN grey

HEPH brown

EARS2 grey

ICMT blue

IARS2 blue

VARS grey

VARS2 grey

LARS2 blue

CARS brown

CARS2 grey

RARS2 grey

WARS brown

WARS2 blue

HARS grey

HARS2 grey

FARSA grey

FARSB blue

FARS2 grey

SARS2 yellow

GATB grey

QRSL1 blue

GATC blue

AARS grey

AARS2 blue

DARS2 blue

NARS blue

NARS2 grey

RDH10 grey

RDH16 grey

CRLF1 grey

RDH14 blue

DHRS3 grey

RDH8 grey

RDH13 grey

MPO grey

ALOX12B grey

EGLN3 grey

EGLN1 blue

EGLN2 yellow

KDM4A blue

KDM8 grey

KDM4C grey

KDM4B grey

KDM4D blue

ALKBH2 grey

ALKBH3 grey

PLOD2 blue

JMJD6 blue

ALKBH1 blue

FTO blue

ALKBH5 grey

FMO4 grey

FMO5 grey

PAM brown

DOHH grey

SOD2 grey

SOD1 grey

SOD3 grey

FTH1 grey

ALDH8A1 grey

GLUD2 grey

LOX grey

LOXL3 green

LOXL2 brown

CYB5R4 blue

CYB5R2 grey

TXNDC17 grey

SUOX grey

QSOX1 grey

QSOX2 blue

GFER grey

NSUN2 blue

TRDMT1 blue

TRMT61A grey

TRMT10A grey

TRMT5 blue

ALKBH8 blue

METTL11B grey

METTL16 grey

METTL3 blue

PCMT1 blue

GLYATL2 brown

ZDHHC2 blue

ZDHHC4 grey

ZDHHC16 grey

ZDHHC17 blue

ZDHHC9 grey

NAA30 blue

NAA40 blue

NAA60 grey

SOAT2 grey

SOAT1 blue

NAT16 grey

CASD1 blue

KAT6B blue

KAT8 blue

KAT2B grey

EP300 blue

HAT1 blue

KAT2A grey

CREBBP blue

NMT1 blue

NMT2 grey

TGM2 grey

TGM3 grey

C1GALT1 grey

C1GALT1C1 grey

B3GALT6 grey

FUT11 blue

POFUT1 blue

POFUT2 blue

B4GALT5 blue

GALNT9 grey

GALNTL5 grey

GALNT2 grey

GALNT3 grey

GALNT4 blue

GALNT6 brown

GALNT11 blue

GALNT12 grey

GALNT13 grey

GALNT14 brown

GALNT1 blue

GALNT7 grey

FUT10 yellow

UGCG grey

B4GALNT4 grey

ART3 grey

ART1 grey

ST6GALNAC6 grey

ST6GALNAC5 green

ST8SIA4 green

FNTA blue

PGGT1B blue

DGKA green

DGKE grey

DGKD blue

DGKG grey

DGKZ brown

DGKH blue

DGKB grey

DGKI brown

DGKQ grey

DGKK grey

DOLK grey

ATG14 blue

UVRAG blue

FN3K grey

FN3KRP grey

GNE grey

PNKP blue

CLP1 blue

ERBB2 grey

CSF1R green

IGF1R grey

MET grey

ROS1 brown

PDGFRB brown

KIT grey

FGFR1 brown

INSRR brown

PDGFRA brown

EPHA1 grey

FGFR2 grey

ERBB3 grey

FGFR4 yellow

FGFR3 grey

EPHA2 grey

EPHA3 brown

EPHA8 yellow

EPHB2 grey

LTK grey

AXL brown

RYK blue

TIE1 brown

FLT4 brown

KDR grey

FLT3 green

EPHB3 grey

EPHA5 grey

EPHB4 grey

EPHB1 grey

EPHA4 grey

ROR1 grey

ROR2 brown

TEK brown

MST1R grey

TYRO3 grey

DDR1 blue

MERTK grey

ERBB4 grey

EPHA7 grey

NTRK3 brown

NTRK2 yellow

DDR2 brown

FLT1 grey

NTRK1 brown

RET grey

EGFR grey

ALK brown

INSR grey

JAK2 green

ABL1 blue

FYN brown

FES grey

YES1 blue

LYN green

HCK green

SRC grey

FER blue

JAK1 blue

TYK2 blue

CSK green

MATK grey

TEC grey

TXK green

ABL2 blue

FRK grey

ZAP70 green

SYK green

BLK green

JAK3 green

ITK green

PTK6 grey

LCK green

BTK green

PTK2 grey

PTK2B green

TGFBR2 blue

BMPR1A blue

AMHR2 grey

TGFBR1 blue

BMPR1B grey

ACVRL1 grey

ACVR1B blue

ACVR1 blue

ACVR2A blue

MOS grey

ILK blue

IRAK2 grey

IRAK1 grey

LIMK2 grey

TESK1 blue

TESK2 grey

RIPK2 grey

RAF1 blue

ARAF grey

BRAF blue

RIPK1 blue

RIPK3 green

WEE1 grey

TNK2 grey

LIMK1 brown

PDPK1 blue

SGK1 grey

AKT3 grey

RPS6KB1 blue

AKT2 yellow

RPS6KA3 blue

PRKX blue

DMPK yellow

RPS6KA2 grey

RPS6KA1 grey

RPS6KB2 grey

RPS6KA6 grey

PSKH1 grey

CHEK1 blue

CASK blue

DCLK1 green

ULK1 grey

STK17B green

CHEK2 grey

DAPK1 blue

PRKAA1 blue

STK17A blue

CSNK1A1 blue

CSNK1D blue

CSNK1E blue

CSNK1G1 blue

CSNK1G3 blue

PAK3 grey

PAK4 blue

PAK1 blue

PAK2 blue

STK39 blue

PRPF4B blue

SRPK3 grey

HIPK2 grey

STK3 blue

STK4 blue

NEK3 grey

NEK2 blue

BUB1 blue

BUB1B blue

STK16 grey

CDC7 blue

TP53RK grey

STK31 grey

TTN grey

TSSK4 grey

TSSK6 grey

STK38 blue

AURKA blue

ULK3 grey

ATM blue

ATR blue

PRKDC blue

SMG1 blue

PIM1 grey

IKBKB blue

CHUK blue

IKBKE blue

PLCL1 grey

PKIA grey

PRKG1 brown

GRK1 grey

GRK7 grey

GRK4 grey

GRK5 brown

GRK6 grey

CAMK2B grey

CAMK2D blue

CAMK1 grey

CAMK4 green

CAMK2A grey

CAMK1D grey

CAMK1G grey

CAMK2G blue

CAMKK2 blue

MYLK grey

MYLK2 brown

EEF2K blue

PLK3 grey

PLK1 blue

PLK4 blue

PLK2 grey

HOXC5 grey

CDKL5 blue

CDK1 blue

CDK4 blue

MAK blue

CDK11B grey

CDK2 blue

CDK8 blue

CDK7 yellow

CDK9 grey

CDK3 grey

CDKL1 grey

CDK6 grey

CDK5 grey

CDK16 blue

CDK17 blue

CDK18 grey

CDK10 grey

CDK15 brown

CDK12 blue

CDK13 blue

MAPK13 grey

MAPK3 blue

MAPK1 blue

MAPK4 grey

MAPK8 blue

MAPK9 blue

MAPK10 brown

MAPK7 blue

MAPK11 grey

MAPK6 blue

MAPK14 blue

MAPK12 grey

MAP3K6 grey

MAP3K3 blue

KSR2 grey

MAP3K1 blue

GSK3B blue

TTBK2 blue

TTBK1 grey

GSK3A blue

ACVR1C grey

FASTK grey

FASTKD2 blue

DYRK1B blue

DYRK3 grey

CLK1 grey

CLK2 blue

CLK3 blue

DYRK1A blue

DYRK2 blue

TTK blue

DYRK4 brown

MAP2K7 blue

MAP2K2 grey

MAP2K4 blue

MAP2K3 grey

MAP2K6 grey

MAP2K1 blue

MAP2K5 grey

MAP3K5 grey

MAP3K2 blue

MAP3K4 blue

PGK2 grey

DLG1 blue

GMPPB grey

PAPOLG blue

MTPAP blue

PAPOLA blue

DNTT grey

GDPGP1 grey

GNPTAB blue

GNPTG grey

SGMS1 blue

SEPHS1 blue

SULT1A2 grey

SULT1A1 grey

SULT1A3 grey

SULT1B1 grey

SULT1C2 grey

SULT1C4 grey

SULT4A1 grey

SULT2A1 grey

SULT1E1 grey

CHST11 blue

CHST12 brown

CHST13 grey

NCEH1 grey

CMBL grey

CLC grey

ACHE grey

BCHE grey

DPH7 blue

ALPL brown

ALPP grey

PPP3R2 grey

DUSP6 grey

DUSP10 grey

DUSP26 grey

DUSP2 green

PTP4A1 grey

DUSP1 grey

PPM1A blue

PPP5C blue

PPP2CA grey

PGAM5 blue

EPHX2 grey

PTPRE grey

PTPN1 blue

CDC25C blue

PTPRR grey

CDC25B blue

CDC25A blue

DUSP9 grey

PTPN5 grey

PTPN7 green

PTPN2 blue

PTPN3 blue

PTPN6 green

PTPN9 blue

PTPN4 blue

PTPN11 blue

PTPN12 blue

PTPN13 brown

PTPN14 blue

PTPN18 grey

PTPN21 blue

PTPN22 green

PTPN23 blue

PTPRA blue

PTPRB brown

PTPRC green

PTPRD grey

PTPRF blue

PTPRG blue

PTPRH grey

PTPRJ blue

PTPRK blue

PTPRM grey

PTPRN2 grey

PTPRN brown

PTPRO green

PTPRQ grey

PTPRS grey

PTPRT grey

PTPRU grey

PTPRZ1 brown

EYA2 grey

ACP1 blue

SMPDL3A grey

TDP1 blue

PLCB1 blue

PLCG2 grey

PLCH1 grey

PLCB3 blue

PLCE1 blue

PLCD1 grey

PLCG1 blue

PLCB4 brown

CNP blue

GDPD2 grey

NAPEPLD blue

STS grey

ARSA grey

PRKCSH grey

TREH grey

MPG grey

SMUG1 grey

CCNO grey

UNG blue

TDG blue

METAP2 blue

METAP1D grey

METAP1 blue

PEPD grey

ACE2 grey

ACE grey

ACR grey

CTSG grey

KLK6 grey

KLKB1 grey

CMA1 grey

F2 grey

REM2 grey

REN grey

NTAN1 grey

WDYHV1 grey

NIT1 grey

TAX1BP3 brown

ACER3 blue

NAAA grey

VNN3 grey

PADI1 grey

PADI3 grey

PADI2 grey

PADI4 grey

PADI6 grey

ADAT1 blue

APOBEC1 grey

ADAR blue

ADARB1 brown

AICDA green

ENTPD7 blue

DCPS grey

ASNA1 grey

HSPA9 grey

WRN blue

BACH1 blue

PIF1 blue

RECQL blue

IGHMBP2 grey

BRIP1 blue

BLM blue

DDX11 grey

DHX16 blue

DDX3X blue

DDX3Y grey

DDX4 grey

DDX25 yellow

EIF4A3 blue

DDX19B grey

DHX9 blue

DDX6 blue

DDX5 blue

UPF1 blue

DDX21 blue

DHX34 blue

DDX58 blue

PEX6 grey

PEX1 blue

RASL11A grey

ARL9 brown

RAB21 blue

RAC3 grey

RHEBL1 brown

RHOH green

RAB37 grey

RHOA blue

KRAS blue

CDC42 blue

RAC1 brown

RAC2 green

RHOB grey

RHOC grey

RAB2A grey

HRAS yellow

RAB2B blue

RAB1B blue

RAB1A blue

RAB8A grey

RAB6B blue

RHEB blue

DNM3 grey

DNM2 blue

DNM1 grey

MX1 grey

DNM1L blue

OPA1 blue

PHPT1 yellow

SGPL1 blue

NPL grey

CLYBL grey

CA14 grey

CA12 grey

CA9 grey

CA2 grey

CA3 grey

CA1 grey

CA4 grey

CA5A grey

CA5B grey

CA6 green

CA7 grey

CA8 grey

CDYL blue

PTS grey

CHAC2 grey

GGACT grey

PDIA3 grey

PDIA2 grey

PDIA4 grey

ERP27 yellow

P4HB grey

TXNDC5 blue

SPAST blue

KATNA1 blue

KATNAL1 blue

KIF16B blue

KIF11 blue

KIF18A blue

KIF1A grey

KIF2C blue

KIF5B blue

CFTR grey

TOP2A blue

TOP2B blue

YARS grey

YARS2 blue

SLC27A4 grey

UBA5 blue

UBA1 blue

UBA3 blue

UBA6 blue

TTL blue

RTCA blue

UQCRB yellow

ATP2C1 blue

ATP2C2 grey

ATP2B1 blue

ATP2B2 grey

ATP2B3 grey

ATP1A1 blue

ATP7B grey

ATP7A blue

HSPA8 grey

TAP1 green

TAP2 green

ABCB9 grey

ABCB10 blue

ABCB8 grey

ATP8B1 blue

PLSCR1 blue

PLSCR2 grey

PLSCR4 grey

ATP8A2 grey

ATP10A grey

ATP8A1 grey

ATP8B2 grey

ATP9A blue

ATP11A blue

ATP11C grey

ATP10D blue

ATP10B grey

ABCC2 grey

ABCB5 grey

ABCC4 grey

ABCC10 blue

ABCC6 grey

ABCC5 blue

TCF4 brown

RALBP1 blue

ZADH2 blue

GEM brown

TMEM54 grey

HCN3 grey

ACYP1 blue

ACYP2 grey

PPP1R3B grey

PYGB brown

LYZL1 grey

GALK2 grey

FAM227B grey

GLB1L grey

TMPPE blue

LALBA grey

GMPPA grey

RNF123 blue

AMIGO3 grey

DLX4 grey

GLOD4 grey

HAGHL grey

CYB5D1 blue

RWDD2A grey

ACAD9 grey

ACPP grey

RBAK blue

PDE6C grey

PDE6A grey

PDE6B grey

IL18BP green

PDE5A grey

PDE6H grey

PDE6D grey

PDE9A yellow

PDE6G green

ALLC grey

TRPM2 green

NUDT14 grey

FAM20B blue

NOL9 blue

FGGY grey

DPYSL2 blue

DPYSL3 brown

APOBEC4 grey

EEF1A2 grey

EEF1A1 yellow

GFM2 grey

EEF2 grey

GFM1 blue

TUFM grey

REV3L blue

POLQ blue

POLB grey

POLI blue

RFC5 blue

POLK blue

POLM blue

POLN grey

MYBBP1A blue

POLG blue

POLL grey

POLH blue

POLG2 blue

ATRIP blue

TREX2 grey

TREX1 blue

ACP2 grey

LGSN grey

LEFTY1 grey

KIAA0319 grey

VPS29 blue

CPNE7 grey

THNSL1 grey

SIRT2 grey

SIRT6 grey

SIRT1 blue

SIRT5 grey

OARD1 grey

MACROD1 grey

SIRT3 grey

MACROD2 grey

SIRT7 grey

DHTKD1 grey

SCCPDH grey

SMYD3 grey

SPCS1 grey

SPCS2 grey

SPCS3 grey

SEC11A grey

SEC11C grey

PLOD1 grey

CERCAM brown

SULT1A4 grey

TMEM91 grey

ALOXE3 grey

TFB1M grey

TARBP1 blue

TRMT11 grey

METTL22 grey

FTSJ1 brown

METTL2A blue

VCPKMT blue

NDUFAF5 grey

METTL4 blue

SETD6 blue

FBL yellow

FTSJ3 blue

PRMT8 brown

NOP2 blue

HEMK1 grey

NSUN4 blue

TRMT13 blue

METTL8 blue

EMG1 yellow

NSUN5 yellow

RRP8 grey

PRMT7 grey

TGS1 blue

METTL5 grey

METTL21C grey

CARM1 blue

SMYD2 grey

METTL21A blue

ECE2 grey

METTL14 blue

MEPCE grey

PRMT2 blue

TYW3 blue

TFB2M grey

PRMT9 blue

METTL2B blue

TRMT10B blue

COMTD1 grey

METTL17 blue

LCMT2 blue

METTL15 blue

METTL7B grey

METTL18 grey

TRMT10C grey

NSUN3 blue

NSUN7 grey

CMTR2 blue

METTL23 blue

PRMT3 blue

METTL7A grey

BCDIN3D grey

FBLL1 grey

PRMT6 grey

LCMT1 yellow

METTL6 blue

NSUN6 grey

MRM1 grey

LRTOMT grey

PRDX6 grey

EPX grey

LPO grey

PXDN grey

PXDNL grey

DNMT3A blue

DNMT3L grey

PRDX1 grey

PRDX5 yellow

PRDX3 grey

PRDX2 grey

NPEPPS blue

GGTLC2 grey

GGT2 grey

GGTLC1 grey

NANP blue

TGDS grey

MANSC1 blue

CHI3L1 grey

MTFMT grey

GALNT16 grey

GALNT18 grey

GALNT15 grey

GALNT5 brown

GALNT10 grey

GALNTL6 grey

POC1B-GALNT4 blue

A4GNT grey

DHDDS grey

ALG13 grey

ALG14 grey

ALG9 grey

ALG8 grey

MGAT4A blue

MGAT4B grey

MGAT4C grey

MANBA blue

ENGASE grey

FUCA2 grey

FUCA1 grey

APOC1 grey

APOC2 grey

APOC3 grey

FGA grey

TFRC blue

APOE grey

FGR green

MUSK grey

STYK1 brown

EPHA6 grey

EPHB6 grey

PTK7 grey

CLK4 grey

SRMS grey

LMTK3 grey

LMTK2 blue

SPRYD3 grey

PBK blue

PEAK1 blue

TNK1 grey

EPHA10 grey

INSL3 grey

CDC14A grey

DUSP13 grey

CDC14B grey

DUSP12 blue

SSH1 blue

CDKN3 blue

EYA1 brown

DUSP3 grey

DUSP16 grey

EYA4 grey

DUSP22 grey

DNAJC6 grey

DUSP4 grey

STYXL1 grey

DUSP5 grey

SSH2 blue

DUSP11 blue

DUSP15 grey

PTPDC1 blue

EYA3 blue

DUSP23 grey

DUSP19 blue

DUSP7 blue

DUSP18 blue

FRMPD2 grey

SSH3 blue

PTP4A2 blue

PTP4A3 grey

DUSP8 grey

DUSP28 grey

DUPD1 grey

DUSP21 grey

DUSP27 grey

CHMP4A grey

TPTE grey

DUSP14 grey

ADPRH blue

CTSA grey

LGMN green

CTSZ grey

CTSH grey

CTSC brown

CTSD grey

NAPSA grey

CTSL grey

CTSV grey

CTSK grey

CTSS green

CTSB grey

TPP1 grey

CTSW green

CTSF grey

CTSE grey

CTSO grey

UEVLD blue

PPA2 grey

PPA1 grey

HIGD2A grey

TECRL grey

FADS6 grey

FADS3 grey

EGFL8 grey

ALOX15B grey

CYP2C18 grey

CYP4Z1 grey

CYP4X1 grey

CYP2F1 grey

CYP2A13 grey

CYP2A7 grey

LIPT1 blue

TBCB yellow

MYO5B blue

CYP4A22 grey

SLC27A1 grey

AASDH blue

RNPEP grey

DPEP3 grey

DPEP2 green

GGTLC3 grey

AADAC grey

DAGLA grey

DAGLB grey

ABHD12 grey

ABHD6 grey

ECI2 grey

KRTAP11-1 grey

CHURC1-FNTB grey

PCYOX1 grey

UGT3A1 grey

UGT3A2 grey

FAXDC2 grey

MAGEA2B grey

MAGEA2 grey

ARSD grey

LIPA grey

DBP grey

GLT8D1 grey

PIGV grey

UGGT2 blue

PIGZ grey

GLT8D2 brown

GCNT7 blue

STT3A blue

UGGT1 grey

B4GALNT3 grey

OGT blue

GBGT1 grey

B3GNT7 grey

POGLUT1 grey

STT3B blue

B4GALNT2 grey

B3GNTL1 grey

B3GNT8 grey

ALG1L grey

GLT6D1 grey

MGAT4D grey

B3GNT9 brown

ALG1L2 brown

ST6GALNAC2 grey

SLC33A1 blue

ABHD5 grey

GK5 blue

LGALS13 yellow

CLCF1 blue

ENPP6 grey

SAPCD1 grey

TICRR blue

GPLD1 grey

HERC3 blue

PYURF grey

PIGW blue

PGAP1 blue

EXTL3 grey

EXTL2 blue

EXT2 brown

EXT1 grey

EXTL1 brown

HS3ST1 grey

HS3ST4 grey

HS3ST5 grey

HS3ST2 grey

HS3ST3B1 grey

HS3ST3A1 brown

HS3ST6 grey

CHST14 blue

DSE brown

CHST3 grey

CHST15 grey

NAGLU grey

CHST1 brown

CES5A grey

ABCD3 blue

PDZD4 grey

SLC6A8 grey

PNCK grey

SSR4 grey

BCAP31 grey

PLXNB3 grey

L1CAM brown

SLC51A grey

SLC51B grey

ABCC11 grey

PXYLP1 brown

ART4 grey

ART5 grey

AASDHPPT blue

SYCE2 grey

PLCB2 green

PLCZ1 grey

PLCH2 grey

PLCL2 grey

PLCD3 blue

PLCXD2 grey

TNS2 grey

NT5DC3 grey

CTDSPL2 blue

PMPCA grey

ASB12 grey

MDP1 grey

HCST green

GGH grey

PCBD2 grey

GLRX2 grey

CYBRD1 brown

DHRS9 grey

RBP2 grey

RBP1 grey

RBP4 grey

LENEP grey

SH3BP1 green

KLHL23 grey

LRP2 grey

CUBN brown

GC grey

EPHX1 grey

NAE1 blue

CA13 grey

ABTB2 grey

KANSL3 blue

SAP130 blue

PRRT2 grey

SETD5 blue

EIF4EBP3 grey

CPA3 grey

KLK1 grey

KLK2 grey

TRHDE grey

TPSD1 grey

TPSAB1 grey

TPSB2 grey

CALM2 blue

CALM3 grey

USP2 grey

USP28 blue

USP36 blue

USP13 grey

OTUD5 grey

USP33 blue

CYLD green

USP40 blue

USP48 blue

USP14 blue

USP11 blue

USP10 blue

USP31 grey

USP42 blue

USP46 blue

USP5 blue

USP4 blue

USP9Y grey

USP34 blue

USP35 grey

USP45 blue

STAMBP blue

USP22 blue

USP9X blue

USP6 grey

USP29 grey

USP26 grey

USP30 grey

PAN2 grey

USP15 blue

USP37 blue

USP44 grey

USP20 blue

STAMBPL1 grey

USP8 blue

USP3 blue

USP21 blue

USP12 blue

USP43 grey

USP25 blue

USP16 blue

USP41 grey

USP24 blue

USP1 blue

USP49 blue

USP38 blue

USP47 blue

USP32 blue

USP19 blue

USP18 grey

USP7 blue

USP17L2 grey

USP51 grey

USP27X grey

HECW1 grey

MYCBP2 blue

MYLIP blue

UBE3C blue

RNF216 blue

UBR7 blue

RNF14 grey

UBR2 blue

RNF19A blue

NEDD4L grey

NEDD4 grey

RNF126 grey

RAD18 blue

HLTF blue

UBE2D1 blue

CHFR blue

MKRN2 blue

UBE2T blue

UBE2A grey

UBE2K blue

ITCH blue

UBE2D4 grey

CNOT4 blue

RNF13 blue

HACE1 blue

HUWE1 blue

BRAP blue

MUL1 grey

RNF31 blue

HECTD1 blue

CDC34 grey

CCNB1IP1 grey

RNF125 grey

MIB1 blue

MID1 grey

MGRN1 blue

STUB1 grey

UBE2I blue

HERC1 blue

UBE2W blue

UBR5 blue

CBLL1 blue

NRF1 blue

UBE2R2 blue

NEURL1 yellow

UBE2S blue

RNF167 grey

SMURF2 blue

UBE2D3 blue

UBE4A blue

CBL blue

RNF8 blue

RNF130 grey

UBE3A blue

CBLB blue

FANCL blue

BIRC6 blue

RNF19B blue

RNF146 blue

UBE2B grey

TRIM32 blue

RBBP6 blue

WWP1 grey

HECTD3 blue

UBR4 blue

HERC2 blue

UBE2M blue

UBE4B blue

UBE2D2 grey

TRAF7 blue

TRIM5 grey

UBE2G1 blue

RNF128 grey

MKRN1 blue

RNF138 blue

MDM2 grey

RNF144B grey

BARD1 blue

HECW2 grey

RNF185 grey

UBE2Q2 blue

CBLC grey

UBR3 blue

RNF217 grey

SHPRH blue

UHRF2 blue

LRSAM1 grey

HERC4 blue

UBE3B blue

RNF144A brown

TRIP12 blue

TRIM11 blue

SH3RF1 blue

SH3RF2 grey

UBE2L6 green

TRIM63 grey

UBE2Z blue

UBR1 blue

AMFR grey

UBE2J2 blue

UBE2Q1 blue

SYVN1 grey

ZSWIM2 brown

RNF149 grey

RNF25 grey

RCHY1 blue

RNF180 grey

HECTD2 blue

RAG1 grey

RNF187 grey

RFWD3 blue

UBE2E3 blue

UBE2E1 blue

RNF139 grey

SH3RF3 grey

UBE2C blue

UBE2O blue

RNF152 grey

UBE2U grey

UBE2N blue

MKRN3 grey

ZNRF2 grey

RNF182 brown

PJA1 blue

SIAH2 grey

RNF41 blue

UBA7 grey

UBE2E2 brown

TTC3 blue

UBE2F grey

UBE2G2 blue

UBE2L3 brown

ZNRF1 grey

UBE2H blue

NHLRC1 grey

RNF133 grey

SIAH1 grey

TRIM33 blue

MIB2 grey

TOPORS blue

WWP2 blue

SMURF1 blue

UBE2J1 blue

DZIP3 blue

PJA2 blue

RNF5 grey

NEURL1B grey

UBE2QL1 grey

RNF103 grey

RNF115 blue

UHRF1 blue

OBSL1 grey

MYLK3 grey

MYLK4 grey

TGM1 grey

TGM5 grey

F13A1 green

F13B grey

TGM7 brown

TGM4 grey

TGM6 grey

FHIT grey

PREP blue

RNGTT blue

SIAE grey

PTRH2 yellow

PTRH1 grey

PGLYRP1 grey

PGLYRP3 grey

PGLYRP2 grey

PGLYRP4 grey

AGA grey

NGLY1 blue

HIF1AN blue

ASPH grey

ANXA3 grey

VKORC1 grey

QPCT grey

PDF grey

AOAH green

CAMKK1 brown

PRKAR2B grey

MAP3K14 grey

MAP3K9 blue

CDKL3 grey

MARK4 grey

CDK11A grey

MAP4K3 blue

MAP4K5 blue

PRKCH green

VRK2 blue

DAPK2 yellow

TRIO blue

EIF2AK2 blue

CDK14 blue

RIOK2 grey

WNK1 blue

PKN2 blue

SLK blue

PRKCQ brown

PRKCZ grey

ROCK1 blue

MAST4 grey

MAP4K4 blue

MYO3B grey

SPEG grey

MARK2 blue

STK10 green

MAP3K13 grey

NUAK1 brown

MARK3 blue

MKNK1 blue

MOK brown

ULK2 grey

MAST2 blue

EIF2AK1 grey

NLK blue

MAPKAPK5 blue

IRAK3 grey

MYO3A grey

SRPK1 blue

MAST3 grey

MKNK2 grey

VRK1 blue

RPS6KA5 blue

SGK2 grey

RIOK3 blue

PIM2 green

STK24 blue

SGK3 grey

MAP4K1 green

VRK3 grey

AURKC grey

MAST1 grey

MAP3K8 grey

PRKAR1A grey

HIPK3 blue

ICK grey

PRKAR2A blue

NEK11 grey

MAPKAPK3 grey

ACVR2B grey

NEK4 blue

PASK blue

STK25 blue

AAK1 blue

MARK1 grey

NEK6 grey

NEK9 blue

MASTL blue

CIT blue

PKN1 grey

RIOK1 blue

GTF2F1 blue

STK35 blue

WNK4 grey

PRKCG grey

PKMYT1 blue

EIF2AK4 blue

STK33 grey

MAP3K10 blue

LATS1 blue

DSTYK blue

ROCK2 blue

ERN2 grey

STK26 grey

TAOK3 blue

SRPK2 blue

MAP3K7 blue

RPS6KC1 blue

NEK1 blue

PAK6 grey

BMP2K blue

CDKL2 grey

MAP3K12 brown

MINK1 blue

HUNK grey

CDC42BPA blue

TLK2 blue

TAF1 blue

PSKH2 grey

TAOK2 blue

LATS2 blue

NEK7 blue

UHMK1 blue

STK32B grey

LRRK1 brown

TNIK blue

CDK20 grey

PINK1 grey

KALRN grey

PKN3 grey

BRSK1 grey

TAOK1 blue

SIK3 blue

NEK8 grey

RPS6KA4 blue

TSSK3 grey

HIPK1 blue

STK36 blue

NUAK2 grey

PRKCI blue

SNRK blue

PRKCD blue

WNK2 grey

MELK blue

STK32C brown

PRKCB green

DAPK3 brown

ULK4 grey

MAP4K2 grey

MLKL green

STK32A brown

SIK2 grey

DCLK2 grey

PRKCE grey

CDC42BPG blue

EIF2AK3 blue

OXSR1 blue

MAP3K11 grey

BRSK2 grey

ERN1 grey

GAK grey

AURKB blue

CSNK1A1L grey

MAP3K15 grey

MAPK15 yellow

AATK grey

RIPK4 grey

TBK1 blue

BCR grey

PRKAR1B grey

SBK1 grey

LRRK2 grey

WNK3 grey

NEK5 grey

IRAK4 blue

PIM3 grey

TLK1 blue

CDC42BPB blue

BMPR2 blue

STK19 grey

CDKL4 grey

STK38L blue

TSSK1B grey

WEE2 grey

PHLPP2 blue

CTDP1 blue

PPP2R3A blue

PPP2R2C grey

PHLPP1 grey

PPEF1 grey

PPM1F brown

PPP3CB blue

PPM1G blue

PPP6C blue

PPP3CC yellow

RPAP2 blue

PPP1R1B brown

ILKAP blue

PPP1R1A grey

PPP2R1B grey

PPM1B blue

CTDSP1 grey

CTDSPL grey

PPP4C yellow

PPP1R3A grey

PPEF2 grey

PPP2R2B grey

SSU72 grey

PPM1M green

UBLCP1 blue

PPP2R3B grey

PPM1D blue

PPM1E grey

CTDSP2 blue

PPP1R2 blue

PDIA5 grey

PDIA6 blue

TMX3 blue

LPL grey

ABCA1 grey

AP1G1 blue

ABCB4 grey

ABCG1 grey

SLC17A6 grey

SLC17A7 brown

SLC17A8 grey

SLC5A6 grey

HAS3 grey

HAS1 brown

HAS2 grey

ATP2B4 grey

ATP2A3 green

ATP2A2 blue

ATP2A1 brown

SLC32A1 grey

SLC52A3 grey

SLC18A1 grey

SLC18A2 grey

SLC18A3 brown

NOS2 grey

NOS1 grey

ABCG5 grey

ABCG8 grey

HIRIP3 grey

ABCD2 green

SLC27A6 brown

HACL1 grey

SLC23A2 grey

SLC23A1 grey

SLC5A1 grey

ABCA8 grey

ABCA3 grey

ABCA4 grey

SLC52A1 grey

SLC52A2 yellow

ABCB6 grey

GM2A green

URAD grey

ABHD4 grey

OSBPL10 grey

OSBPL5 blue

OSBPL8 blue

CHST9 grey

CHST7 brown

SLC25A16 grey

UST grey

FFAR1 grey

SLC35D1 grey

CHAC1 grey

SLC35D2 grey

HS2ST1 blue

HS6ST1 grey

HS6ST2 grey

CHST2 brown

CHST5 grey

CHST6 grey

TSPO grey

SLC10A2 grey

SLC25A10 grey

PON1 grey

PON2 grey

PON3 grey

ABHD3 blue

MFSD2A grey

PITPNM1 brown

PITPNM2 grey

PITPNM3 grey

OSBPL2 blue

OSBPL3 blue

OSBPL6 blue

OSBPL7 grey

OSBPL9 grey

OSBPL1A blue

OSBP blue

UCP1 brown

UCP2 green

UCP3 blue

SLC25A27 grey

SLC25A14 grey

CHRM3 grey

ADRA2A green

ADRA2C grey

PDZD11 grey

ARV1 grey

CTRC grey

MMAB grey

SLC26A1 grey

SLC26A2 blue

SLC35B2 blue

SLC35B3 blue

SULT6B1 grey

TPST1 grey

TPST2 brown

CDO1 grey

SLC25A1 grey

SLC25A11 grey

SLC25A12 grey

SLC25A13 grey

SLC37A4 grey

SLC37A1 blue

SLC37A2 green

ABHD10 grey

SLC22A5 blue

SLC25A20 grey

SLC25A21 grey

SLC46A1 grey

SLC25A32 grey

VKORC1L1 blue

BSG grey

SLC16A1 grey

SLC16A8 grey

SLC16A3 grey

VDAC1 grey

MPC1 grey

MPC2 grey

UQCRH grey

UQCR11 yellow

UQCR10 grey

UQCRQ grey

UQCRC1 grey

UQCRC2 grey

UQCRFS1 grey

CYC1 grey

MT-CYB grey

BCO2 grey

SLC5A5 grey

SLC3A2 grey

SLC7A5 brown

SLC36A4 blue

SLC25A15 grey

SLC25A2 grey

SLC19A2 grey

SLC19A3 grey

SLC25A19 blue

ZDHHC21 blue

SLC2A1 grey

MAN2C1 grey

MAN2B1 blue

MAN2B2 grey

SAMHD1 green

SLC2A2 grey

SLC25A4 grey

ARL2 yellow

ARL2BP blue

SLC25A5 grey

SLC25A6 yellow

CACNA1C brown

CACNA2D2 grey

CACNA1D blue

CACNA1A grey

CACNA1E grey

ITPR1 grey

ITPR2 grey

ITPR3 blue

RGN grey

H6PD grey

FUK grey

GGCX grey

DNMT1 blue

DNMT3B grey

CKMT1B grey

TXNDC12 blue

GALNT8 grey

CHST10 grey

CMAS grey

PIGO blue

GPAA1 grey

PIGK blue

FAM213B grey

ACSM5 grey

ACSM2A grey

ACSM6 grey

ACSM4 grey

ALPPL2 grey

NUS1 blue

FNTB blue

ZMPSTE24 blue

RCE1 blue

CYCS grey

TARS blue

TARS2 grey

TARSL2 grey

GARS blue

PARS2 blue

DHPS grey

NDST3 brown

NDST4 brown

DNASE1 grey

ENDOV grey

DICER1 blue

DROSHA blue

LYZ grey

LYG2 grey

NEIL2 grey

NEIL1 grey

OGG1 grey

LNPEP grey

CPN1 grey

UCHL1 brown

UCHL3 grey

UCHL5 grey

BAP1 blue

YOD1 blue

HPN grey

ST14 grey

TMPRSS6 grey

MBTPS1 blue

KLK7 grey

KLK8 grey

KLK13 grey

F7 grey

F9 grey

PREPL blue

F11 grey

KLK11 grey

CELA1 green

ELANE grey

F12 grey

C1R grey

C1S grey

C5 grey

CFI grey

CFD grey

CFB grey

C3 grey

LONP1 grey

F10 grey

PCSK9 grey

PLAT brown

PROC grey

PLG grey

PLAU brown

FURIN grey

KLK3 grey

GZMM green

GZMA green

GZMB green

TMPRSS15 grey

CASP1 green

ESPL1 blue

CAPN3 grey

CASP2 blue

CASP3 blue

CASP6 blue

CASP7 blue

CASP8 blue

CASP9 grey

CASP10 blue

BACE2 grey

BACE1 blue

MME grey

ADAMTS2 brown

ADAMTS3 grey

ADAMTS14 brown

THOP1 grey

MMP13 brown

MMP3 grey

MEP1A grey

MEP1B grey

BMP1 grey

MMP7 grey

MMP2 brown

MMP8 green

MMP9 grey

IDE blue

MMP12 green

ECE1 grey

PAPPA brown

MMP14 brown

MBTPS2 blue

ADAM17 blue

ADAMTS13 grey

DCP2 blue

PARK7 grey

CRISP3 grey

MIA3 grey

PGM5 grey

TNNI3K grey

FPGT-TNNI3K grey

DHRS2 grey

HTATIP2 grey

MDH1B grey

CTBP1 blue

HSD11B1L grey

SDR9C7 grey

SDR42E1 grey

WWOX grey

ECHDC2 grey

ST20 grey

TAF9 grey

TNRC6B blue

NME5 blue

RPUSD4 grey

CRCP grey

GOT1L1 grey

GLYATL1 grey

UBAC2 grey

NAA10 yellow

NAA11 grey

NAA15 blue

NAA20 grey

ARID4B blue

TRMT12 grey

MOXD1 brown

CRYZ grey

CHST8 grey

CHST4 grey

GAL3ST2 grey

GAL3ST3 grey

HS6ST3 grey

GAL3ST4 green

DCT grey

ATG9B grey

IREB2 blue

COX7A2 grey

COX4I2 grey

COX6A2 grey

COX6B2 grey

COX7A1 grey

COX7B2 grey

COX8C grey

ZDHHC6 blue

HHAT grey

NAT14 yellow

SH3GLB1 blue

ZDHHC8 grey

PORCN brown

ZDHHC15 grey

YKT6 brown

NAT9 yellow

NAA50 blue

NAT10 blue

ESCO1 blue

NAT8 grey

ZDHHC7 blue

ZDHHC5 blue

ZDHHC1 blue

ZDHHC12 grey

ZDHHC3 blue

ZDHHC19 grey

ESCO2 blue

ZDHHC24 grey

ZDHHC14 grey

ZDHHC13 blue

ZDHHC22 brown

MBOAT4 grey

ZDHHC20 blue

ZDHHC23 blue

SATL1 grey

ZDHHC11 grey

GLYATL3 grey

ZDHHC18 grey

ZDHHC11B grey

SLC25A29 grey

SLC22A9 grey

SLC22A1 grey

SLC22A4 grey

ECH1 grey

STARD7 blue

STARD8 grey

STARD13 grey

STAR grey

STARD9 grey

STARD4 grey

STARD5 grey

STARD6 grey

STARD10 grey

RPP14 grey

SCARF2 brown

SLC7A9 grey

SLCO4A1 grey

CYB5R1 grey

CYB5RL blue

HPS3 blue

FTMT grey

RLBP1 grey

RBP3 grey

NLN grey

CPA1 yellow

CPA2 yellow

CPA5 grey

CPA6 grey

PARP3 grey

PARP12 grey

PARP4 blue

ZC3HAV1 blue

PARP11 green

PARP2 blue

PARP6 grey

PARP1 blue

PARP8 blue

TIPARP grey

PARP14 grey

PARP15 green

FKBP4 grey

FKBP6 grey

FKBP7 brown

FKBP1A brown

FKBP5 grey

PPIL2 blue

FKBP3 grey

PIN4 grey

FKBP8 grey

FKBP14 brown

PPIF grey

NKTR blue

FKBP1B grey

FKBP9 blue

PIN1 yellow

PPIL4 blue

FKBP11 grey

PPIG blue

FKBP10 grey

CWC27 blue

PPIB grey

PPIC blue

PPID grey

PPIH blue

FKBP2 grey

PPIL6 grey

PPIA yellow

PPIAL4G grey

PPIAL4C grey

PPIAL4E grey

KAT6A blue

GTF3C4 blue

CDY2A grey

ELP3 grey

CLOCK blue

KAT7 blue

KAT5 blue

MGMT grey

BRPF1 blue

TTLL10 grey

RNMT blue

CMTR1 blue

QTRT1 yellow

TRMT1 yellow

TRMT1L blue

METTL1 yellow

TRMU blue

TRIT1 blue

PUS1 yellow

NQO2 grey

ATE1 blue

MSRA grey

RCL1 grey

NAGPA grey

DPH5 grey

SLC8B1 grey

SLC47A1 grey

SLC47A2 grey

SLC22A2 grey

SLC22A18 grey

SLC41A1 blue

SLC41A2 grey

SLC22A7 grey

SLC22A8 grey

SLC22A6 grey

NPC1L1 grey

SCARB1 grey

FABP3 grey

CD36 grey

FABP2 grey

FABP1 grey

FABP7 brown

FABP5 brown

FABP6 grey

FABP12 grey

FABP9 grey

AQP7 grey

AQP3 grey

GLTP brown

SLC22A10 grey

SLC26A3 grey

SLC26A8 grey

SLC26A7 grey

SLC26A9 grey

SLC26A11 blue

SLC16A6 grey

SLC16A7 grey

SLC16A4 grey

SLC16A5 grey

FOLR1 grey

FOLR3 grey

SLC2A9 grey

SLC2A11 grey

SLC2A8 grey

SLC2A5 grey

SLC2A7 grey

SLC6A13 grey

SLC6A12 grey

SLC6A11 grey

SLC6A1 grey

SLC28A2 grey

SLC28A1 grey

SLC28A3 grey

SLC29A2 grey

SLC7A2 grey

SLC2A3 grey

SLC2A10 grey

SLC2A13 blue

RHAG grey

AQP6 grey

AQP8 grey

AQP10 grey

AQP5 grey

AQP2 grey

AQP4 grey

AQP1 grey

SLC17A2 grey

SLC17A3 grey

SLC17A1 grey

SLC34A1 grey

SLC17A4 grey

SLC34A2 grey

SLC20A1 grey

SLC20A2 grey

SLC34A3 grey

SLC7A11 grey

SLC1A3 brown

SLC1A6 grey

SLC1A1 grey

SLC1A2 grey

SLC1A7 grey

SLC13A3 grey

SLC13A2 grey

AQP9 grey

SLC14A2 grey

SLC14A1 grey

SLC44A1 blue

SLC44A2 blue

SLC44A5 grey

SLC44A3 grey

SLC44A4 grey

SLC12A2 grey

SLC12A1 grey

SLC5A9 grey

SLC2A4 grey

SLC22A3 grey

SLC29A1 grey

FOLR2 green

SLC5A2 grey

SLC2A12 brown

SLC2A6 grey

SLC2A14 yellow

SLC29A4 grey

SLC38A4 grey

SLC7A1 blue

SLC7A3 brown

SLC36A1 blue

SLC43A1 grey

SLC7A7 green

SLC43A2 grey

SLC16A10 grey

SLC7A10 grey

SLC6A18 grey

SLC7A8 brown

SLC6A15 grey

SLC1A5 brown

SLC38A1 blue

SLC1A4 grey

SLC38A2 grey

SLC6A19 grey

SLC6A14 grey

SLC38A5 brown

SLC38A3 grey

SLC6A7 brown

SLC6A20 grey

SLC9A3 grey

SLC9A1 blue

SLC9A2 grey

SLC9A5 brown

SLC9A4 grey

SLC9A9 green

SLC4A7 blue

SLC4A4 grey

SLC4A5 grey

SLC5A12 grey

SLC5A10 grey

SLC5A11 grey

SLC6A6 brown

SLC6A9 grey

SLC6A5 grey

SLC3A1 grey

SLC7A6 grey

SLC11A1 grey

SLC11A2 blue

SLC40A1 grey

SLC39A14 yellow

SLC39A8 grey

SLC39A5 grey

SLC39A6 blue

SLC39A3 grey

SLC39A1 blue

SLC39A4 grey

SLC39A2 green

SLC39A10 blue

SLC12A7 grey

SLC12A4 grey

SLC12A5 grey

SLC12A6 blue

SLC13A5 grey

SLCO1C1 brown

SLC16A2 grey

SLCO3A1 blue

SLCO2A1 grey

SLCO2B1 grey

SLC6A2 grey

SLC6A3 grey

SLC6A4 grey

SLC30A1 grey

SLC36A3 grey

SLC36A2 grey

TRNT1 blue

SLC12A3 green

SLC4A1 grey

CELSR3 grey

SLC26A4 grey

SLC4A9 grey

SLC4A3 grey

SLC4A2 blue

SLC26A6 grey

SLC4A8 brown

SLC4A10 brown

SLC8A3 brown

SLC8A2 yellow

SLC8A1 green

SLC24A1 blue

SLC24A4 green

SLC24A2 grey

SLC24A3 grey

SLC24A5 grey

SLC15A1 grey

SLC15A2 grey

SLC19A1 grey

SLC13A1 grey

SLC13A4 grey

SLC5A7 grey

SLC22A16 grey

SLC22A15 grey

SLC5A3 blue

SLC10A6 grey

SLC22A12 grey

SLC22A11 grey

ARCN1 blue

COPE yellow

COPA blue

COPB1 blue

COPG2 blue

COPG1 blue

COPB2 blue

SLC25A26 grey

DBI grey

FOSL1 brown

SLC25A42 grey

RHBG grey

RHCG grey

SLC25A3 grey

STARD3 grey

SFXN1 grey

SFXN3 brown

SLC25A22 grey

SLC25A18 grey

SLC25A38 grey

SLC16A12 grey

SLC16A9 grey

SLC16A13 grey

SLC25A37 grey

SLC25A28 grey

HIF1A blue

SLC35A1 grey

NUP50 blue

NUP188 blue

NUP93 brown

NUP88 grey

NUP155 blue

NUP153 blue

NUP214 blue

NUP210 blue

NUP54 blue

NUP210L grey

RANBP2 blue

NUP205 blue

NUP35 blue

NUP62CL grey

NUP62 blue

LENG9 grey

NUP160 blue

TPR blue

NDC1 blue

NUP133 blue

NUP37 blue

SEH1L blue

AAAS blue

RAE1 blue

NUP98 blue

NUP107 blue

GLE1 blue

NUP43 blue

NUP85 blue

AHCTF1 blue

SEC13 grey

POM121 blue

HDLBP blue

NPC1 blue

SLC17A5 grey

SLC29A3 grey

SLC35A3 blue

SLC35B4 grey

SLC35A2 blue

SLC9A7 blue

SLC9A8 grey

SLC35C1 grey

KPNA6 blue

XPO7 yellow

XPO6 blue

SLC30A6 blue

SLC30A7 blue

SLC39A7 grey

CINP grey

ANAPC11 yellow

SLC15A3 green

SLC15A4 grey

AMN grey

**Supplementary Table 2: Differentially expressed genes in high and low MRGPI risk groups**

Gene logFC PValue FDR Group

ESR1 -3.501415302 1.85E-16 3.64E-12 Low-group

CYP3A4 -6.326163347 5.21E-15 5.13E-11 Low-group

HMGA2 7.142776917 7.92E-15 5.21E-11 High-group

MEX3A 2.013691418 1.92E-14 9.48E-11 High-group

GCK -7.191210494 6.08E-14 2.40E-10 Low-group

C16orf89 -7.25164674 4.40E-13 1.45E-09 Low-group

PMEL -5.62947787 6.25E-13 1.76E-09 Low-group

MTHFD1 -1.926637155 2.25E-12 5.53E-09 Low-group

MROH2A -5.370855199 3.27E-12 7.17E-09 Low-group

RANBP3L -3.525004069 6.35E-12 1.24E-08 Low-group

SEZ6 6.880823428 6.93E-12 1.24E-08 High-group

MLANA -5.608133092 8.32E-12 1.37E-08 Low-group

SLC6A14 8.181402939 1.42E-11 2.09E-08 High-group

ANGPTL7 -9.254053363 1.50E-11 2.09E-08 Low-group

TRIM63 -8.029139091 1.59E-11 2.09E-08 Low-group

PLEKHB1 4.137355348 2.06E-11 2.54E-08 High-group

IGFN1 -8.476610036 2.42E-11 2.81E-08 Low-group

ASPA -2.41477235 3.71E-11 4.07E-08 Low-group

ARHGEF2 1.394957392 5.14E-11 5.33E-08 High-group

G6PD 2.253189061 6.52E-11 6.43E-08 High-group

TUSC5 -9.822903642 9.43E-11 8.65E-08 Low-group

CEP55 2.104973104 9.66E-11 8.65E-08 High-group

CYP2C8 -3.087734098 1.23E-10 1.05E-07 Low-group

ECM2 -1.995601855 1.32E-10 1.08E-07 Low-group

MAPK13 2.622206105 1.54E-10 1.17E-07 High-group

GABARAPL1 -1.762583583 1.54E-10 1.17E-07 Low-group

SEC14L2 -2.579290383 1.65E-10 1.20E-07 Low-group

RCAN1 -1.637881658 1.86E-10 1.31E-07 Low-group

CR2 5.594995114 2.14E-10 1.46E-07 High-group

HTR3A 7.527719926 3.70E-10 2.43E-07 High-group

SLC26A9 5.26358689 4.23E-10 2.69E-07 High-group

KIF2C 1.981440705 4.96E-10 3.05E-07 High-group

SPIB 3.793399201 5.34E-10 3.11E-07 High-group

TMEM100 -3.122256513 5.37E-10 3.11E-07 Low-group

DMBT1 7.217496197 5.92E-10 3.34E-07 High-group

CDCA7 3.054443532 7.95E-10 4.30E-07 High-group

RASGRF1 -4.314093695 8.07E-10 4.30E-07 Low-group

SLC16A11 -2.914601738 8.92E-10 4.54E-07 Low-group

CYP4V2 -1.586262112 9.04E-10 4.54E-07 Low-group

LIPE -3.450498787 9.28E-10 4.54E-07 Low-group

PDK4 -2.315246675 9.44E-10 4.54E-07 Low-group

HADH -1.233870283 9.95E-10 4.59E-07 Low-group

TMOD1 -2.439428079 1.00E-09 4.59E-07 Low-group

PACRG -3.137890797 1.03E-09 4.61E-07 Low-group

S100A14 2.782740836 1.37E-09 5.99E-07 High-group

GHR -2.210821928 1.46E-09 6.20E-07 Low-group

PADI1 -6.233523648 1.48E-09 6.20E-07 Low-group

ATP6V0D2 3.948536709 1.54E-09 6.32E-07 High-group

ZNF711 2.936187483 1.57E-09 6.34E-07 High-group

TRIP13 2.019650516 1.71E-09 6.63E-07 High-group

SLC1A5 1.980198537 1.72E-09 6.63E-07 High-group

GPR160 2.064160072 1.93E-09 7.33E-07 High-group

CYP8B1 -3.85127427 2.14E-09 7.94E-07 Low-group

RBP2 5.99766201 2.31E-09 8.43E-07 High-group

NUF2 1.988506658 2.60E-09 9.18E-07 High-group

ARID3A 2.345591737 2.61E-09 9.18E-07 High-group

PEBP4 -4.705815703 2.89E-09 9.61E-07 Low-group

MT-CYB -1.360723651 2.93E-09 9.61E-07 Low-group

OMG -4.253606587 2.95E-09 9.61E-07 Low-group

TMEM65 1.424942404 2.96E-09 9.61E-07 High-group

ALPI 8.012415694 2.98E-09 9.61E-07 High-group

KLKB1 -1.850539565 3.28E-09 1.04E-06 Low-group

TMEM155 4.85007288 3.34E-09 1.05E-06 High-group

C6 -2.435076335 3.52E-09 1.08E-06 Low-group

TMEM27 -2.910415695 3.98E-09 1.20E-06 Low-group

TRIM72 4.449990242 4.04E-09 1.20E-06 High-group

HSPB6 -4.130400977 4.08E-09 1.20E-06 Low-group

MPDZ -1.811924597 4.22E-09 1.20E-06 Low-group

ADRA1B -2.03159461 4.24E-09 1.20E-06 Low-group

PKIB 2.861432071 4.25E-09 1.20E-06 High-group

LMOD1 -2.734074613 4.78E-09 1.31E-06 Low-group

NDRG1 1.713552955 4.83E-09 1.31E-06 High-group

IGDCC4 3.316350226 4.92E-09 1.31E-06 High-group

SOX4 1.715040451 4.93E-09 1.31E-06 High-group

USP2 -2.106810819 5.21E-09 1.37E-06 Low-group

DLX6 5.708372773 5.40E-09 1.40E-06 High-group

KCNN2 -2.869658579 5.54E-09 1.42E-06 Low-group

DRD1 -3.746678572 5.72E-09 1.44E-06 Low-group

EXPH5 -2.778781725 6.35E-09 1.59E-06 Low-group

ADH1B -2.882236529 6.51E-09 1.59E-06 Low-group

CPED1 -2.254236861 6.52E-09 1.59E-06 Low-group

AGL -1.633790716 6.63E-09 1.59E-06 Low-group

RHOV 3.961827963 8.47E-09 2.01E-06 High-group

MMP10 3.554851179 9.04E-09 2.12E-06 High-group

CCK 7.269886605 9.19E-09 2.13E-06 High-group

CXorf66 -3.371015507 9.35E-09 2.14E-06 Low-group

SLC46A3 -2.125532559 9.59E-09 2.17E-06 Low-group

COLCA2 2.322035124 1.03E-08 2.31E-06 High-group

ETFDH -1.214773335 1.05E-08 2.33E-06 Low-group

GSTZ1 -1.785491182 1.07E-08 2.33E-06 Low-group

FABP3 -3.75054998 1.07E-08 2.33E-06 Low-group

PLEKHS1 4.593001369 1.14E-08 2.43E-06 High-group

TPRG1 -2.198437665 1.15E-08 2.43E-06 Low-group

KEL 4.13229441 1.18E-08 2.48E-06 High-group

SP8 5.800431699 1.20E-08 2.48E-06 High-group

CRAT -1.135302851 1.34E-08 2.74E-06 Low-group

ENPEP -1.915783823 1.35E-08 2.74E-06 Low-group

CALCR 4.578934843 1.42E-08 2.86E-06 High-group

CBLN4 -4.524852228 1.48E-08 2.92E-06 Low-group

SLC12A1 -5.704305597 1.49E-08 2.92E-06 Low-group

OPRK1 7.325731421 1.50E-08 2.92E-06 High-group

FRAS1 3.339084883 1.52E-08 2.92E-06 High-group

TIMP4 -3.821328415 1.58E-08 2.96E-06 Low-group

ALDH2 -1.335177697 1.58E-08 2.96E-06 Low-group

HHATL -7.306260655 1.62E-08 2.98E-06 Low-group

GARNL3 -1.840354564 1.65E-08 2.99E-06 Low-group

LGALS12 -5.085428976 1.66E-08 2.99E-06 Low-group

SLC5A6 -1.763625069 1.71E-08 3.02E-06 Low-group

F11 -1.712418829 1.72E-08 3.02E-06 Low-group

TDRD9 4.000276965 1.72E-08 3.02E-06 High-group

MYBL2 2.126563135 1.79E-08 3.12E-06 High-group

ABCG4 -3.600893079 1.81E-08 3.13E-06 Low-group

RHOB -1.429775879 1.84E-08 3.16E-06 Low-group

CTNNA3 -3.020104463 2.42E-08 4.11E-06 Low-group

SRSF12 1.876203094 2.62E-08 4.42E-06 High-group

FABP4 -3.176208061 2.67E-08 4.46E-06 Low-group

PLAC9 -3.450610799 2.69E-08 4.46E-06 Low-group

HID1 1.814591194 2.82E-08 4.63E-06 High-group

COL9A2 2.274829669 2.98E-08 4.85E-06 High-group

TTLL4 1.624472969 3.04E-08 4.91E-06 High-group

TSC22D3 -1.499755566 3.16E-08 5.00E-06 Low-group

MGAT4C -4.998664189 3.16E-08 5.00E-06 Low-group

NRN1 -2.376565928 3.17E-08 5.00E-06 Low-group

SPINT1 3.098885743 3.22E-08 5.04E-06 High-group

GTSE1 1.740037777 3.27E-08 5.07E-06 High-group

CDX2 4.671264685 3.34E-08 5.12E-06 High-group

BCAS4 1.55672743 3.35E-08 5.12E-06 High-group

CALML3 -5.531028927 3.56E-08 5.36E-06 Low-group

GALNT15 -2.132987939 3.56E-08 5.36E-06 Low-group

HJURP 1.644472772 3.90E-08 5.81E-06 High-group

LEP -6.698360589 3.93E-08 5.81E-06 Low-group

GOT2 -1.439128569 3.95E-08 5.81E-06 Low-group

MYL9 -3.318398823 3.98E-08 5.81E-06 Low-group

NEFH 3.264345071 4.31E-08 6.21E-06 High-group

FAM13A -1.900652452 4.32E-08 6.21E-06 Low-group

SULT1B1 -2.73568517 4.38E-08 6.21E-06 Low-group

DNAJC25 -1.135986621 4.38E-08 6.21E-06 Low-group

CDCP1 2.7214757 4.53E-08 6.39E-06 High-group

MLYCD -1.199626633 4.85E-08 6.75E-06 Low-group

CA4 -3.635929979 4.86E-08 6.75E-06 Low-group

UCK2 1.213614272 4.99E-08 6.88E-06 High-group

CD1A 3.487001395 5.07E-08 6.94E-06 High-group

ESRP1 4.735877436 5.13E-08 6.98E-06 High-group

TTYH1 4.098424187 5.31E-08 7.17E-06 High-group

SLC2A4 -2.146564308 5.44E-08 7.30E-06 Low-group

PTHLH 3.234976843 5.63E-08 7.50E-06 High-group

ZNF385C 2.351083618 5.80E-08 7.67E-06 High-group

FCN2 -3.439827685 5.87E-08 7.71E-06 Low-group

GPR50 9.641933247 6.01E-08 7.85E-06 High-group

VSIG2 -2.825011148 6.07E-08 7.87E-06 Low-group

TRDN -7.126780708 6.24E-08 8.03E-06 Low-group

CPEB3 -1.502491403 6.38E-08 8.17E-06 Low-group

CYP19A1 4.177773822 6.46E-08 8.22E-06 High-group

HBA2 -2.676221086 6.59E-08 8.33E-06 Low-group

MT-ND5 -1.519752223 6.76E-08 8.47E-06 Low-group

NDRG4 -3.652392533 6.82E-08 8.47E-06 Low-group

ABHD1 -2.570084736 6.88E-08 8.47E-06 Low-group

HBB -2.093999597 6.94E-08 8.47E-06 Low-group

LGI1 -3.614033926 6.96E-08 8.47E-06 Low-group

SLC27A5 -2.324544655 7.05E-08 8.53E-06 Low-group

GLI2 -4.433052727 7.13E-08 8.57E-06 Low-group

SEL1L3 2.233455393 7.22E-08 8.63E-06 High-group

MCM4 1.266228021 7.35E-08 8.72E-06 High-group

TNFAIP6 3.384155145 7.60E-08 8.97E-06 High-group

SLC22A1 -3.233866021 7.92E-08 9.29E-06 Low-group

IKBKE 1.564496309 8.65E-08 1.01E-05 High-group

HDC -2.329843912 8.97E-08 1.04E-05 Low-group

CBR4 -1.173049361 9.05E-08 1.04E-05 Low-group

MT-CO2 -1.179769352 9.20E-08 1.05E-05 Low-group

GAS2L3 1.447462785 9.25E-08 1.05E-05 High-group

NR0B1 5.266861711 9.37E-08 1.06E-05 High-group

ALAD -1.273156552 9.49E-08 1.07E-05 Low-group

PNPLA5 -5.123381566 9.55E-08 1.07E-05 Low-group

TTPAL -1.590325183 1.00E-07 1.11E-05 Low-group

GSTM5 -3.808422756 1.01E-07 1.12E-05 Low-group

HIBADH -1.219384707 1.02E-07 1.12E-05 Low-group

CNDP1 -3.796011472 1.03E-07 1.12E-05 Low-group

LPA -2.503423479 1.03E-07 1.12E-05 Low-group

TNP1 11.2976749 1.08E-07 1.16E-05 High-group

ACYP2 -1.028560076 1.11E-07 1.20E-05 Low-group

FBXO40 -5.178223772 1.12E-07 1.20E-05 Low-group

PINK1 -1.365932806 1.13E-07 1.21E-05 Low-group

MUC12 -4.577007187 1.16E-07 1.23E-05 Low-group

GFRA3 4.662065706 1.19E-07 1.26E-05 High-group

FXYD3 3.292089405 1.23E-07 1.29E-05 High-group

FDCSP 6.029323253 1.25E-07 1.31E-05 High-group

SAPCD2 1.950700931 1.29E-07 1.33E-05 High-group

ACKR2 -2.011868003 1.29E-07 1.33E-05 Low-group

MT-CO1 -1.162874979 1.30E-07 1.33E-05 Low-group

MCEMP1 3.597768023 1.40E-07 1.43E-05 High-group

ZNF239 2.228944032 1.51E-07 1.53E-05 High-group

SLC8A3 -2.8331982 1.51E-07 1.53E-05 Low-group

ARHGEF38 2.819885848 1.55E-07 1.56E-05 High-group

WDR86 -3.36456996 1.61E-07 1.61E-05 Low-group

AURKB 1.757264882 1.69E-07 1.68E-05 High-group

CES5A -2.512247619 1.75E-07 1.73E-05 Low-group

NCEH1 1.933725296 1.77E-07 1.74E-05 High-group

RFC4 1.125177831 1.88E-07 1.85E-05 High-group

ACAA2 -1.22173148 1.92E-07 1.87E-05 Low-group

GLDN 2.914619084 1.93E-07 1.87E-05 High-group

SLC7A10 4.622996463 1.93E-07 1.87E-05 High-group

IGF2BP2 2.077313314 1.99E-07 1.91E-05 High-group

LMNB1 1.325086623 2.00E-07 1.91E-05 High-group

CD19 3.238977318 2.01E-07 1.91E-05 High-group

CTSG -3.727599292 2.04E-07 1.93E-05 Low-group

4-Sep -1.990903526 2.04E-07 1.93E-05 Low-group

KNTC1 1.343548336 2.06E-07 1.93E-05 High-group

FRMD3 -2.053635989 2.11E-07 1.96E-05 Low-group

KDM8 -1.67133463 2.12E-07 1.96E-05 Low-group

POF1B 3.090243708 2.14E-07 1.97E-05 High-group

ERICH4 3.791043317 2.20E-07 1.99E-05 High-group

CDC14B -1.458712201 2.20E-07 1.99E-05 Low-group

SC5D -1.202210538 2.20E-07 1.99E-05 Low-group

GRHPR -1.200959073 2.27E-07 2.04E-05 Low-group

ESPL1 1.620520106 2.29E-07 2.05E-05 High-group

PZP -3.604167597 2.31E-07 2.05E-05 Low-group

PITPNM2 -1.209217525 2.31E-07 2.05E-05 Low-group

DMRT2 5.144992352 2.49E-07 2.20E-05 High-group

CTH -1.968958935 2.50E-07 2.20E-05 Low-group

TPSAB1 -3.417161795 2.53E-07 2.22E-05 Low-group

LAMP3 2.089330263 2.57E-07 2.25E-05 High-group

EPHA5 6.351718261 2.60E-07 2.26E-05 High-group

KIF18A 1.559106841 2.66E-07 2.30E-05 High-group

TNFSF4 1.81447663 2.74E-07 2.35E-05 High-group

BTD -1.181931776 2.75E-07 2.36E-05 Low-group

CDC20 1.829869823 2.80E-07 2.39E-05 High-group

CFAP61 3.572174131 2.85E-07 2.41E-05 High-group

MAFA -4.71762071 2.86E-07 2.41E-05 Low-group

BBOX1 -2.492830884 2.86E-07 2.41E-05 Low-group

UGT2B17 -4.244616336 2.89E-07 2.41E-05 Low-group

ACER1 -3.954475387 2.92E-07 2.43E-05 Low-group

ACSM2A -2.117471882 2.94E-07 2.44E-05 Low-group

CD300LG -3.346461022 2.98E-07 2.46E-05 Low-group

ZNF93 1.858066771 3.01E-07 2.47E-05 High-group

NEFL 4.435860879 3.06E-07 2.50E-05 High-group

CLSTN2 -3.131658361 3.09E-07 2.52E-05 Low-group

ACE2 -3.387229332 3.11E-07 2.52E-05 Low-group

C6orf132 2.911960197 3.16E-07 2.55E-05 High-group

UPP2 -3.33800692 3.16E-07 2.55E-05 Low-group

NLRP6 -2.616983747 3.23E-07 2.59E-05 Low-group

WNT3A 5.52736763 3.24E-07 2.59E-05 High-group

TMEM61 2.635115189 3.29E-07 2.62E-05 High-group

GLP1R 4.273394046 3.32E-07 2.63E-05 High-group

ANGPT4 -3.907101486 3.40E-07 2.68E-05 Low-group

IVD -1.172388805 3.41E-07 2.68E-05 Low-group

OGDHL -2.035509612 3.46E-07 2.71E-05 Low-group

MT-ND4 -1.216460201 3.54E-07 2.75E-05 Low-group

FNDC5 -3.292019699 3.59E-07 2.79E-05 Low-group

GJB6 5.326681475 3.68E-07 2.84E-05 High-group

JPH2 -3.09393686 3.91E-07 3.01E-05 Low-group

BUB1B 1.558374429 4.03E-07 3.09E-05 High-group

BCL2L14 2.627567135 4.15E-07 3.17E-05 High-group

CHGA 4.564811358 4.33E-07 3.29E-05 High-group

NKX2-5 5.419525738 4.51E-07 3.42E-05 High-group

SPOCK3 -7.727235336 4.53E-07 3.42E-05 Low-group

KIF23 1.536705198 4.56E-07 3.43E-05 High-group

FER1L5 1.93426634 4.63E-07 3.47E-05 High-group

ITGB4 1.76751331 4.75E-07 3.55E-05 High-group

MRO -1.596048747 4.90E-07 3.64E-05 Low-group

ACO1 -1.107877908 5.11E-07 3.78E-05 Low-group

SRD5A1 -1.465662445 5.13E-07 3.79E-05 Low-group

MAN1C1 -1.636988839 5.21E-07 3.83E-05 Low-group

ADH1C -2.866568349 5.29E-07 3.88E-05 Low-group

SGOL2 1.303474073 5.32E-07 3.88E-05 High-group

DMGDH -1.730462532 5.54E-07 4.03E-05 Low-group

TCL1A 4.197667825 5.64E-07 4.09E-05 High-group

TFDP3 -6.718470128 5.76E-07 4.16E-05 Low-group

FAM149A -1.301037459 6.09E-07 4.38E-05 Low-group

EHHADH -1.746562747 6.36E-07 4.56E-05 Low-group

COX7A1 -3.27380707 6.40E-07 4.57E-05 Low-group

TICRR 1.569099743 6.52E-07 4.64E-05 High-group

CHEK1 1.217375044 6.61E-07 4.69E-05 High-group

CYP2U1 -1.373197402 6.85E-07 4.84E-05 Low-group

FCN3 -2.364028955 6.99E-07 4.92E-05 Low-group

NEURL3 2.139881967 7.24E-07 5.08E-05 High-group

MT-ND6 -1.621123725 7.43E-07 5.19E-05 Low-group

B3GALT1 -4.687979701 7.54E-07 5.25E-05 Low-group

RNF152 -1.34102853 7.60E-07 5.27E-05 Low-group

SHC4 -2.824578318 7.65E-07 5.29E-05 Low-group

SRC 1.361166888 7.78E-07 5.36E-05 High-group

ST18 3.638600812 8.21E-07 5.62E-05 High-group

KIF18B 1.687188258 8.23E-07 5.62E-05 High-group

TMEM88 -1.556159817 8.29E-07 5.63E-05 Low-group

ACSL1 -1.63801557 8.46E-07 5.73E-05 Low-group

DEXI -1.021157791 8.95E-07 6.04E-05 Low-group

TNNC1 2.874348484 8.98E-07 6.04E-05 High-group

CACYBP 1.01196081 9.16E-07 6.14E-05 High-group

DEPDC1B 1.86156374 9.21E-07 6.16E-05 High-group

POR -1.122433362 9.27E-07 6.17E-05 Low-group

CHRND 4.648060565 9.38E-07 6.22E-05 High-group

CIDEA -6.616038227 9.73E-07 6.38E-05 Low-group

OR11A1 5.975175069 9.74E-07 6.38E-05 High-group

ARHGAP11B 1.289097634 9.82E-07 6.41E-05 High-group

BSPRY 2.429458686 9.95E-07 6.47E-05 High-group

CYP39A1 -2.562190503 1.02E-06 6.58E-05 Low-group

LHFPL3 4.594069135 1.02E-06 6.58E-05 High-group

PDE11A -2.076424888 1.03E-06 6.62E-05 Low-group

TG -2.517369261 1.05E-06 6.76E-05 Low-group

TREM1 2.925901544 1.06E-06 6.80E-05 High-group

TRIM59 1.534003776 1.08E-06 6.88E-05 High-group

ORC1 1.570714467 1.08E-06 6.90E-05 High-group

A4GNT 4.487993624 1.11E-06 7.06E-05 High-group

HELLS 1.408424368 1.12E-06 7.07E-05 High-group

HOXD1 3.051844098 1.13E-06 7.11E-05 High-group

FANCD2 1.279979676 1.13E-06 7.11E-05 High-group

GLYATL1 -2.058714973 1.14E-06 7.14E-05 Low-group

NOS1 -3.619797151 1.15E-06 7.14E-05 Low-group

ABCB4 -2.184042572 1.16E-06 7.21E-05 Low-group

UHRF1 1.677332025 1.18E-06 7.30E-05 High-group

RUNDC3B -1.919288078 1.21E-06 7.45E-05 Low-group

LUZP2 -3.948123046 1.21E-06 7.47E-05 Low-group

GABRA2 5.805540116 1.22E-06 7.52E-05 High-group

XRCC2 1.585214734 1.27E-06 7.77E-05 High-group

KLF9 -1.414900242 1.30E-06 7.91E-05 Low-group

SLC25A34 -1.563988822 1.35E-06 8.21E-05 Low-group

AHSP -4.649062172 1.38E-06 8.36E-05 Low-group

SYNGR3 2.863329335 1.40E-06 8.47E-05 High-group

TWIST2 -3.532098174 1.42E-06 8.59E-05 Low-group

KLK6 5.877455248 1.43E-06 8.60E-05 High-group

DMBX1 3.286631746 1.48E-06 8.86E-05 High-group

SYT8 4.31025272 1.48E-06 8.87E-05 High-group

GADD45A -1.241641011 1.52E-06 9.06E-05 Low-group

HAVCR1 3.296998373 1.56E-06 9.26E-05 High-group

MS4A1 3.567403951 1.57E-06 9.30E-05 High-group

AMACR -1.795748291 1.58E-06 9.30E-05 Low-group

PGRMC1 -1.110894133 1.59E-06 9.38E-05 Low-group

CYP3A43 -2.603436025 1.61E-06 9.44E-05 Low-group

STARD5 -1.578816218 1.62E-06 9.47E-05 Low-group

IGSF1 2.780276577 1.65E-06 9.61E-05 High-group

NWD1 -3.615711729 1.67E-06 9.65E-05 Low-group

MYBPC2 3.634775775 1.68E-06 9.69E-05 High-group

HLF -1.879313532 1.73E-06 9.96E-05 Low-group

HMX3 5.255426544 1.74E-06 9.98E-05 High-group

HAGH -1.100349358 1.75E-06 0.000100017 Low-group

TPSB2 -3.094377368 1.80E-06 0.000102936 Low-group

C8A -1.774738255 1.82E-06 0.000103517 Low-group

KIF11 1.318570629 1.82E-06 0.000103554 High-group

PNMA3 2.94048787 1.86E-06 0.000105384 High-group

TMEM233 -1.744375053 1.89E-06 0.000106493 Low-group

SKA3 1.629029773 1.89E-06 0.000106497 High-group

RNASE2 2.761984597 1.90E-06 0.000106497 High-group

RORA -1.334250487 1.98E-06 0.000110409 Low-group

ISM2 4.83447782 2.00E-06 0.000110932 High-group

DAPL1 -6.835933967 2.02E-06 0.000111583 Low-group

CACNA1I -3.657910279 2.02E-06 0.000111583 Low-group

CMA1 -3.828601913 2.03E-06 0.000111583 Low-group

CYP26B1 2.691656751 2.04E-06 0.000112269 High-group

KCNMA1 -1.716610271 2.06E-06 0.000112979 Low-group

ST3GAL6 -1.525636808 2.07E-06 0.000113277 Low-group

CD22 2.332744716 2.12E-06 0.000115414 High-group

MAL2 1.447474777 2.13E-06 0.000115479 High-group

AKAP6 -1.535267598 2.17E-06 0.000117708 Low-group

SPINT2 2.429688471 2.19E-06 0.000118009 High-group

SEC14L3 -3.138747277 2.20E-06 0.000118009 Low-group

MFAP3L -2.0348044 2.20E-06 0.000118009 Low-group

GYS2 -2.76520715 2.20E-06 0.000118009 Low-group

PTCH2 -2.662549489 2.21E-06 0.000118145 Low-group

KCNJ8 -1.614450594 2.23E-06 0.000118705 Low-group

DLK1 6.214719732 2.24E-06 0.000119085 High-group

CYP2B6 -2.486706606 2.25E-06 0.000119238 Low-group

PLK1 1.533400522 2.28E-06 0.000120403 High-group

TROAP 1.60540854 2.32E-06 0.000122511 High-group

LTB 2.168128512 2.36E-06 0.000123824 High-group

SERTM1 -6.817141317 2.39E-06 0.000125031 Low-group

CRTAC1 -3.57196728 2.40E-06 0.000125031 Low-group

EPM2A -1.095330421 2.45E-06 0.000127593 Low-group

ECT2 1.317768011 2.46E-06 0.00012768 High-group

UBE2T 1.324628851 2.58E-06 0.000133477 High-group

TMEM130 2.726965128 2.59E-06 0.000133612 High-group

SLC14A2 -3.123169647 2.61E-06 0.000134436 Low-group

SMR3A -7.07978137 2.62E-06 0.000134596 Low-group

SH3BP1 1.364986431 2.64E-06 0.000134915 High-group

SEMA6A 1.96373963 2.67E-06 0.000136266 High-group

CRLF2 3.623809599 2.68E-06 0.000136738 High-group

DHRS1 -1.184027383 2.73E-06 0.000138344 Low-group

IGFBP4 -1.301930889 2.77E-06 0.000140202 Low-group

MT-ND4L -1.391473822 2.81E-06 0.000141365 Low-group

TCTEX1D4 -2.209192076 2.83E-06 0.000141566 Low-group

FRMD7 -4.551246481 2.83E-06 0.000141566 Low-group

TRIM17 2.357296767 2.85E-06 0.000142105 High-group

RAD54L 1.587232828 2.93E-06 0.000146001 High-group

TMEM132C -5.648942405 2.97E-06 0.000147337 Low-group

UST -2.568193654 2.98E-06 0.000147337 Low-group

TMEM237 1.007222381 3.06E-06 0.000150681 High-group

LAPTM4B 1.506880674 3.11E-06 0.000152984 High-group

FHL5 -2.459464389 3.15E-06 0.000154636 Low-group

CDH23 -1.447600356 3.17E-06 0.000155259 Low-group

RNF103-CHMP3 2.348102628 3.19E-06 0.000155528 High-group

CDCA8 1.332866581 3.21E-06 0.000155706 High-group

ALDH6A1 -1.55074025 3.21E-06 0.000155706 Low-group

SELENBP1 -1.291284371 3.23E-06 0.000155751 Low-group

PDE2A -1.439153566 3.23E-06 0.000155751 Low-group

CHML 1.329836881 3.25E-06 0.000156259 High-group

DZIP1L 1.888300752 3.26E-06 0.000156259 High-group

MFI2 1.8503258 3.27E-06 0.00015654 High-group

STARD6 3.256365788 3.29E-06 0.000156821 High-group

GNA14 -1.587518496 3.32E-06 0.000157829 Low-group

RASAL1 2.54521655 3.32E-06 0.000157829 High-group

CHD5 2.85521793 3.33E-06 0.000157829 High-group

UGT1A7 3.689124839 3.37E-06 0.000158831 High-group

TWIST1 -3.514566132 3.37E-06 0.000158831 Low-group

EZH2 1.196168302 3.38E-06 0.000158831 High-group

SUCO 1.067759117 3.44E-06 0.000161051 High-group

TREML2 3.251192343 3.45E-06 0.000161051 High-group

SPEF2 1.565422663 3.46E-06 0.000161051 High-group

NEK2 1.556847786 3.47E-06 0.000161335 High-group

SUCNR1 2.629099907 3.57E-06 0.000165704 High-group

FANCB 1.351874709 3.59E-06 0.00016606 High-group

RECQL4 1.308230036 3.60E-06 0.00016606 High-group

TRO -1.717320062 3.61E-06 0.00016606 Low-group

MT-ATP6 -1.123447722 3.63E-06 0.000166997 Low-group

CCDC146 -1.543226959 3.65E-06 0.000167228 Low-group

ZFP92 2.177977064 3.66E-06 0.000167542 High-group

CTSO -1.196672264 3.78E-06 0.00017178 Low-group

NREP 1.271106031 3.92E-06 0.000177035 High-group

RCL1 -1.239142738 3.95E-06 0.000178222 Low-group

ARMC3 2.721013427 3.96E-06 0.000178222 High-group

FHDC1 1.903807477 4.04E-06 0.000180759 High-group

CASQ2 -2.398240918 4.04E-06 0.000180759 Low-group

REN -2.9517863 4.07E-06 0.000181458 Low-group

CDC25A 1.384385485 4.08E-06 0.000181543 High-group

RIPK2 1.119191401 4.13E-06 0.000183481 High-group

SORD -1.38876963 4.15E-06 0.000183701 Low-group

CD81 -1.074066344 4.17E-06 0.000184223 Low-group

VEPH1 2.610094477 4.23E-06 0.000186591 High-group

PRR11 1.307734218 4.25E-06 0.000186816 High-group

SPDYC -3.07638891 4.30E-06 0.000188945 Low-group

KIAA0101 1.277033437 4.37E-06 0.000191378 High-group

CFI -1.337637616 4.39E-06 0.000191378 Low-group

MT-CO3 -1.098766763 4.39E-06 0.000191378 Low-group

PEG10 3.616225552 4.48E-06 0.00019466 High-group

FAM60A 1.24395133 4.50E-06 0.000194921 High-group

HIC2 1.325275857 4.53E-06 0.000195833 High-group

NDC80 1.428388959 4.57E-06 0.000196484 High-group

SLC6A12 -1.695015172 4.58E-06 0.000196484 Low-group

SPATA18 -2.103638803 4.59E-06 0.000196484 Low-group

C11orf80 1.245420556 4.60E-06 0.000196484 High-group

TMEM156 2.419874235 4.67E-06 0.000199302 High-group

FANCI 1.10950866 4.73E-06 0.00020049 High-group

CYP26A1 -2.935284576 4.73E-06 0.00020049 Low-group

GPHN -1.264677397 4.74E-06 0.00020049 Low-group

CDK5R2 6.062053356 4.74E-06 0.00020049 High-group

KIF20A 1.411970317 4.83E-06 0.000204065 High-group

RCOR2 2.165546249 4.88E-06 0.000205332 High-group

FGF8 5.672166649 4.89E-06 0.000205332 High-group

C6orf195 2.614470157 4.90E-06 0.000205332 High-group

ALAS1 -1.193646922 4.98E-06 0.000208597 Low-group

SLC6A15 6.197662294 5.03E-06 0.000209937 High-group

KCNK10 2.782015786 5.04E-06 0.000210093 High-group

OAF -1.062508225 5.06E-06 0.000210468 Low-group

ARL14 3.822239711 5.07E-06 0.000210468 High-group

SPHK1 2.085712068 5.12E-06 0.000211847 High-group

MEP1B -2.871387163 5.14E-06 0.000212304 Low-group

DSCC1 1.192340003 5.15E-06 0.000212304 High-group

STON1-GTF2A1L -4.641880088 5.20E-06 0.000213627 Low-group

CDC6 1.421663965 5.22E-06 0.000213852 High-group

SLC7A1 1.808298655 5.23E-06 0.000213882 High-group

PEX11G -1.30630818 5.32E-06 0.000216067 Low-group

HTR2B -2.337966429 5.33E-06 0.000216067 Low-group

ANLN 1.523734584 5.33E-06 0.000216067 High-group

ANO9 2.123289524 5.45E-06 0.000219999 High-group

ADIPOQ -6.69626269 5.49E-06 0.000221242 Low-group

ACOX1 -1.036655728 5.61E-06 0.000225411 Low-group

GNE -1.295887206 5.63E-06 0.000225732 Low-group

S100A9 2.757895635 5.72E-06 0.000228711 High-group

BTNL9 -1.580115504 5.77E-06 0.000230165 Low-group

PTPRT 3.391674066 5.78E-06 0.000230165 High-group

CNGB1 2.834618308 5.89E-06 0.000234082 High-group

CCNB1 1.317144801 6.10E-06 0.000241575 High-group

SPEG 2.921619174 6.15E-06 0.000242841 High-group

SLCO1A2 -2.270803417 6.19E-06 0.000243915 Low-group

DLX5 3.086355253 6.24E-06 0.000244664 High-group

C14orf159 -1.073262761 6.24E-06 0.000244664 Low-group

RSPO2 -4.361612616 6.26E-06 0.000244664 Low-group

BUB1 1.384967152 6.27E-06 0.000244664 High-group

GABRA4 5.481153338 6.35E-06 0.000247547 High-group

PYGM -2.341517468 6.38E-06 0.000247894 Low-group

ADI1 -1.159071638 6.41E-06 0.000247931 Low-group

C12orf49 1.065651923 6.41E-06 0.000247931 High-group

ORC6 1.530143076 6.43E-06 0.000247931 High-group

CYP2A13 -4.212509023 6.43E-06 0.000247931 Low-group

YPEL1 -1.671268179 6.54E-06 0.000251947 Low-group

LCAT -1.710608355 6.58E-06 0.000252868 Low-group

FCRL1 3.580844838 6.81E-06 0.000260942 High-group

MAGEB17 -4.44934798 6.86E-06 0.000260942 Low-group

C11orf96 -1.659353204 6.87E-06 0.000260942 Low-group

ZNF391 2.020004055 6.87E-06 0.000260942 High-group

LRRC1 1.466313002 6.88E-06 0.000260942 High-group

IGF1 -1.832713116 6.88E-06 0.000260942 Low-group

FAM159A 2.315660618 6.95E-06 0.000262907 High-group

CA9 3.590376954 7.15E-06 0.000269911 High-group

OR2H1 4.003304549 7.29E-06 0.000274814 High-group

PLXNA3 1.237290266 7.43E-06 0.000279623 High-group

AGTR2 -6.340026589 7.45E-06 0.000279849 Low-group

ATP6V0E2 -1.302063735 7.48E-06 0.000280309 Low-group

CENPF 1.436590482 7.52E-06 0.000281371 High-group

MMP9 1.831074876 7.61E-06 0.000283529 High-group

IQGAP3 1.519538482 7.63E-06 0.0002837 High-group

AQP6 -4.537787809 7.82E-06 0.00028973 Low-group

CDH10 4.888479612 7.86E-06 0.000290746 High-group

SLIT1 2.241973243 7.90E-06 0.000291701 High-group

SORBS2 -1.158447768 7.94E-06 0.000292661 Low-group

OFCC1 3.961601277 7.97E-06 0.000293151 High-group

MCM6 1.116453992 8.00E-06 0.000293484 High-group

TDRD5 3.747402292 8.01E-06 0.000293545 High-group

ZNF124 1.252052039 8.09E-06 0.000295758 High-group

PPAPDC3 -2.429090179 8.11E-06 0.000296211 Low-group

MCM2 1.232414094 8.18E-06 0.000298148 High-group

ERVFRD-1 -2.606941839 8.22E-06 0.000299023 Low-group

CLEC3B -1.852472876 8.24E-06 0.000299192 Low-group

TNFRSF13C 2.45080067 8.29E-06 0.000300542 High-group

ALAS2 -2.495570442 8.37E-06 0.000302807 Low-group

ALDH4A1 -1.340412574 8.41E-06 0.000302933 Low-group

BICC1 2.198709523 8.48E-06 0.0003045 High-group

CD79A 2.801996208 8.58E-06 0.000307544 High-group

P2RX2 -4.700920369 8.63E-06 0.000308467 Low-group

CELF3 3.527094844 8.64E-06 0.000308467 High-group

TRIM67 2.307679306 8.67E-06 0.000308816 High-group

ANXA10 -2.306049266 8.68E-06 0.000308816 Low-group

UBE2C 1.599226316 8.69E-06 0.000308816 High-group

SCTR 2.914643834 8.90E-06 0.000314813 High-group

EXO1 1.415349702 8.93E-06 0.000314813 High-group

BAIAP2L2 1.7259708 8.94E-06 0.000314813 High-group

TEX15 3.95969233 8.96E-06 0.000314813 High-group

STK39 1.485970519 8.97E-06 0.000314813 High-group

KLC3 2.502027947 9.01E-06 0.000315413 High-group

PDCD1 2.290741174 9.08E-06 0.000317472 High-group

P2RX3 -1.920944978 9.12E-06 0.000318101 Low-group

BPIFA1 6.884420492 9.14E-06 0.00031848 High-group

TRNP1 2.073391887 9.28E-06 0.000322662 High-group

SYN2 -3.000711334 9.67E-06 0.000335519 Low-group

LGALS14 6.82672748 9.71E-06 0.000336368 High-group

SHOX2 2.656445116 9.79E-06 0.000338657 High-group

CLDN18 3.179520959 9.82E-06 0.000338782 High-group

ADCYAP1 -2.682801424 9.83E-06 0.000338782 Low-group

VSTM4 -1.502006312 9.85E-06 0.000338782 Low-group

MFSD6 1.090669755 9.93E-06 0.000340463 High-group

SFI1 1.149814878 1.00E-05 0.000342006 High-group

FAM227A 1.845814719 1.00E-05 0.000342006 High-group

CCT6B -1.512018464 1.00E-05 0.000342006 Low-group

CYP2W1 2.269567216 1.01E-05 0.00034465 High-group

SLC30A8 4.141130303 1.02E-05 0.000345258 High-group

BLM 1.307049408 1.02E-05 0.000345542 High-group

PSORS1C1 2.198797603 1.02E-05 0.000345542 High-group

RXFP4 2.8909889 1.02E-05 0.000345542 High-group

NLGN1 3.879658173 1.04E-05 0.000350024 High-group

HMGB2 1.08384339 1.04E-05 0.000350024 High-group

LMOD2 -3.354718945 1.05E-05 0.00035263 Low-group

FOXP3 -2.133155411 1.05E-05 0.000353139 Low-group

RP1 -3.92517334 1.06E-05 0.000353139 Low-group

PPM1L -1.164003108 1.06E-05 0.000353294 Low-group

METTL7A -1.000795529 1.06E-05 0.000353719 Low-group

TRIM71 3.155187338 1.06E-05 0.000353719 High-group

CCL14 -1.649353711 1.07E-05 0.000354479 Low-group

KIF4A 1.467074758 1.07E-05 0.000354479 High-group

ZNF385B -2.021289668 1.07E-05 0.0003553 Low-group

DOK6 2.177441174 1.08E-05 0.000357042 High-group

GRIP1 2.569992341 1.09E-05 0.00036107 High-group

C12orf75 2.027774587 1.11E-05 0.000364693 High-group

CAGE1 3.048092743 1.11E-05 0.000364693 High-group

C1orf116 2.23428441 1.11E-05 0.00036484 High-group

ATAD2 1.101303786 1.12E-05 0.000366851 High-group

MUC6 4.250767152 1.14E-05 0.000373413 High-group

BAAT -1.495825847 1.15E-05 0.00037372 Low-group

AXDND1 1.615418475 1.17E-05 0.000380008 High-group

SPACA7 -3.224010836 1.18E-05 0.000381564 Low-group

CRHBP -2.428012594 1.19E-05 0.000385043 Low-group

HMGA1 1.149156334 1.20E-05 0.000385043 High-group

SEPP1 -1.157072393 1.21E-05 0.000387499 Low-group

SHCBP1 1.695007212 1.21E-05 0.000389011 High-group

RP11-468E2.6 -1.996779429 1.22E-05 0.000391628 Low-group

CLEC4G -2.794413825 1.23E-05 0.000393744 Low-group

CCDC74B 2.839623015 1.24E-05 0.000394432 High-group

PEG3 2.850597677 1.24E-05 0.000394432 High-group

SH3D21 1.320291637 1.24E-05 0.000394432 High-group

FAM129C 2.560690193 1.25E-05 0.000395841 High-group

NUGGC -1.95669684 1.25E-05 0.000396884 Low-group

CXCL5 4.042572477 1.25E-05 0.000396884 High-group

TLDC2 1.453994884 1.28E-05 0.000404039 High-group

DCAF13 1.019389093 1.30E-05 0.000406562 High-group

ADRA1A -2.548844428 1.30E-05 0.000406562 Low-group

KLK13 4.344175516 1.34E-05 0.000416792 High-group

NR3C2 -1.324159426 1.35E-05 0.000419654 Low-group

ROS1 -3.284843265 1.36E-05 0.000421186 Low-group

SIX3 3.883229715 1.36E-05 0.000421186 High-group

TRPV5 2.974810984 1.37E-05 0.000426013 High-group

SYN3 1.963386777 1.38E-05 0.000428474 High-group

ARRDC4 -1.635775086 1.39E-05 0.000428474 Low-group

TAF7L -2.074720332 1.39E-05 0.000428792 Low-group

CTSF -1.223957868 1.39E-05 0.000428792 Low-group

NXNL1 -6.942079549 1.40E-05 0.000428792 Low-group

PFKP 1.964397271 1.40E-05 0.000428792 High-group

CLDN5 -1.745646677 1.41E-05 0.000432056 Low-group

KIAA1524 1.373366391 1.43E-05 0.000437513 High-group

SLC24A5 -5.634531493 1.45E-05 0.00044421 Low-group

TAS1R3 2.456086049 1.48E-05 0.000450545 High-group

WNT8B -2.662237301 1.49E-05 0.000452845 Low-group

IL17RB 1.474466831 1.51E-05 0.000457222 High-group

FAM153A 2.512715052 1.51E-05 0.000457996 High-group

TP53INP2 -1.075027 1.54E-05 0.000464171 Low-group

SLC6A13 -1.869368877 1.55E-05 0.000466952 Low-group

ZNF680 -1.085673837 1.57E-05 0.000471278 Low-group

CLC -5.965305996 1.59E-05 0.000476742 Low-group

CENPK 1.376123955 1.59E-05 0.000477798 High-group

TGM4 3.629654656 1.61E-05 0.000480332 High-group

EPPK1 2.068524887 1.62E-05 0.000484078 High-group

MISP 3.453547717 1.62E-05 0.000484078 High-group

SLC9A2 2.549619754 1.63E-05 0.000485117 High-group

CSDC2 -2.970233946 1.64E-05 0.000487419 Low-group

ASB15 -4.453096741 1.65E-05 0.000488791 Low-group

SMIM22 3.551203271 1.66E-05 0.00049199 High-group

NUDT11 2.678619239 1.67E-05 0.000495505 High-group

GRID2IP 2.010764449 1.68E-05 0.000497376 High-group

MSMO1 -1.150185468 1.69E-05 0.000498352 Low-group

DPF3 -1.660995609 1.70E-05 0.000501106 Low-group

GALNT7 1.727384508 1.71E-05 0.000502964 High-group

KCTD17 1.498794505 1.72E-05 0.000505917 High-group

SCGB1A1 4.654494431 1.75E-05 0.000512079 High-group

CCL19 3.191909463 1.76E-05 0.000513901 High-group

COL25A1 -2.423772415 1.76E-05 0.000513901 Low-group

GOLGA6A -2.6641914 1.77E-05 0.000517934 Low-group

MATN3 3.129646401 1.80E-05 0.000524464 High-group

HIST1H4E 1.926689452 1.80E-05 0.000524464 High-group

ZPLD1 3.427789059 1.81E-05 0.000525084 High-group

APOA5 -1.812050166 1.81E-05 0.000525084 Low-group

PSAPL1 4.201418059 1.81E-05 0.000525643 High-group

FCHO1 1.671308995 1.82E-05 0.000525963 High-group

TM6SF2 -1.492758329 1.85E-05 0.000535011 Low-group

PCDHA5 2.907021549 1.88E-05 0.000542458 High-group

RTKN2 1.734403868 1.89E-05 0.000543048 High-group

WNT2B -2.227889574 1.94E-05 0.000558233 Low-group

CADPS 3.201729502 1.97E-05 0.000563925 High-group

LPIN1 -1.197560516 1.97E-05 0.000564349 Low-group

GMPR -2.346076226 1.98E-05 0.000566844 Low-group

GADD45G -1.371775686 2.00E-05 0.000567454 Low-group

SLCO2A1 -1.71938879 2.00E-05 0.000567454 Low-group

SELE -2.100935296 2.00E-05 0.000567454 Low-group

ABAT -1.56640289 2.00E-05 0.000567454 Low-group

SDR16C5 5.498951562 2.00E-05 0.000567454 High-group

LDHD -1.321365592 2.01E-05 0.000570129 Low-group

QDPR -1.071699939 2.02E-05 0.000570763 Low-group

CFAP57 -1.882488176 2.04E-05 0.000576311 Low-group

CDK1 1.381840662 2.08E-05 0.000584567 High-group

KCNE1 -1.849129869 2.11E-05 0.000590743 Low-group

SLC1A2 -2.395080425 2.14E-05 0.000600312 Low-group

NCAPG 1.437049757 2.15E-05 0.000602104 High-group

CKAP4 1.051260604 2.18E-05 0.000607963 High-group

STIL 1.180655809 2.20E-05 0.000609083 High-group

B3GNT3 2.188762353 2.20E-05 0.000609356 High-group

BANK1 2.669696174 2.22E-05 0.000611934 High-group

ZC3HAV1L 1.730493199 2.24E-05 0.000616542 High-group

UNC13D 1.787523761 2.27E-05 0.000623784 High-group

CCDC170 -1.805448523 2.28E-05 0.000624557 Low-group

AQP9 -2.181706437 2.30E-05 0.000629121 Low-group

ADAD2 -1.796239138 2.30E-05 0.000629121 Low-group

RP11-178L8.4 -1.793879041 2.37E-05 0.000643153 Low-group

FCRL2 3.40568542 2.37E-05 0.000643153 High-group

UBXN10 -2.120832201 2.37E-05 0.000643153 Low-group

SH2D5 2.295392526 2.38E-05 0.000643153 High-group

BCL11A 1.907790561 2.39E-05 0.000645077 High-group

FUT4 1.478461058 2.40E-05 0.00064799 High-group

ARHGAP36 -6.834249039 2.43E-05 0.000653659 Low-group

C1R -1.401830267 2.43E-05 0.000653794 Low-group

CBX2 1.540377917 2.44E-05 0.000656162 High-group

PFKFB1 -1.861059087 2.46E-05 0.000658534 Low-group

GNAO1 -2.129375746 2.46E-05 0.000658616 Low-group

SLC23A2 -1.215726687 2.48E-05 0.00066455 Low-group

ABCG2 -1.637627652 2.50E-05 0.000664632 Low-group

CDCA2 1.4770805 2.50E-05 0.000664632 High-group

ST8SIA2 -4.015655978 2.50E-05 0.000664632 Low-group

DHODH -1.409830861 2.51E-05 0.000667294 Low-group

CD7 2.012890629 2.52E-05 0.000669185 High-group

SDC1 -1.056013452 2.53E-05 0.00067118 Low-group

SUPT3H 1.007456854 2.57E-05 0.000679914 High-group

LPCAT1 1.116997109 2.57E-05 0.000679914 High-group

ACSL6 -1.897121658 2.59E-05 0.000683592 Low-group

MUC1 2.846853782 2.61E-05 0.000686789 High-group

MYRIP -1.726241402 2.67E-05 0.000701094 Low-group

KRBA1 1.262935574 2.68E-05 0.000702434 High-group

SLC38A1 1.712613753 2.72E-05 0.00071139 High-group

CCNJL 1.979221986 2.73E-05 0.000711501 High-group

KLHL23 1.053324101 2.76E-05 0.000717401 High-group

HAO1 -1.630699838 2.77E-05 0.000718256 Low-group

SMYD1 -3.748982478 2.78E-05 0.000718256 Low-group

ABCC6 -1.187274662 2.78E-05 0.000719109 Low-group

ITM2C 1.056783543 2.82E-05 0.000727303 High-group

ACSM2B -1.669706431 2.87E-05 0.000738295 Low-group

HMX2 5.406599686 2.88E-05 0.000739161 High-group

GINS1 1.329464028 2.88E-05 0.000739161 High-group

ALOX15B -2.990812237 2.89E-05 0.000740666 Low-group

CYP2A6 -2.988706281 2.90E-05 0.000742714 Low-group

KIAA1614 1.496475362 2.94E-05 0.000752136 High-group

CKMT2 -2.5419344 2.94E-05 0.000752136 Low-group

RSPO3 -3.136619319 2.95E-05 0.000752136 Low-group

MXRA8 -1.908155683 2.95E-05 0.000752722 Low-group

TMEM25 -1.661513951 2.97E-05 0.000755096 Low-group

KHK -1.361554413 2.97E-05 0.000755096 Low-group

PHYHD1 -1.975687271 2.98E-05 0.000757972 Low-group

NLRP14 -2.262719655 2.99E-05 0.000758716 Low-group

CLSPN 1.548081845 3.00E-05 0.000758716 High-group

HBA1 -3.117792639 3.00E-05 0.000758716 Low-group

FBP1 -1.520984712 3.00E-05 0.000758716 Low-group

TFR2 -1.424017237 3.01E-05 0.000758716 Low-group

ZNF692 1.009750664 3.01E-05 0.000758716 High-group

ATP6V1G3 3.886775236 3.03E-05 0.0007618 High-group

DEPDC7 -1.374521604 3.05E-05 0.000766315 Low-group

AVPR1A -3.037116241 3.06E-05 0.000766334 Low-group

HS3ST4 6.227213897 3.07E-05 0.000769814 High-group

ENPP1 -1.147762514 3.08E-05 0.000771156 Low-group

GPIHBP1 -1.393833235 3.09E-05 0.000771156 Low-group

FCGR2B -2.215767344 3.12E-05 0.000777609 Low-group

AMZ1 2.483307879 3.12E-05 0.000777865 High-group

KLHL14 3.257205567 3.13E-05 0.000777891 High-group

P2RY6 1.716925882 3.13E-05 0.000777891 High-group

NUSAP1 1.003217659 3.14E-05 0.000778374 High-group

RAB25 3.771244122 3.14E-05 0.000778754 High-group

KRTAP4-1 5.133769696 3.18E-05 0.000786602 High-group

PFKFB4 1.435255102 3.18E-05 0.000787172 High-group

PARPBP 1.204261409 3.19E-05 0.000788569 High-group

PLEKHG4B 3.115256459 3.20E-05 0.000788961 High-group

TGFA 1.796839226 3.21E-05 0.000791126 High-group

MRC1 -1.305642944 3.32E-05 0.000814062 Low-group

RACGAP1 1.038190801 3.32E-05 0.000814062 High-group

CCNA2 1.503468078 3.34E-05 0.000818955 High-group

HP -1.981037754 3.41E-05 0.000833829 Low-group

C6orf222 3.638186488 3.43E-05 0.000838231 High-group

CADM1 -1.103204434 3.47E-05 0.000845374 Low-group

SRPX -2.01972314 3.48E-05 0.000847483 Low-group

POU2AF1 2.102678938 3.52E-05 0.000856015 High-group

TMC4 2.205802281 3.52E-05 0.000856397 High-group

ARHGEF39 1.125910043 3.53E-05 0.000856397 High-group

WISP2 -2.98817556 3.54E-05 0.000856568 Low-group

ASNS 1.818030889 3.56E-05 0.000859679 High-group

RAD51 1.172318295 3.56E-05 0.000859679 High-group

PCBP3 -2.475270304 3.57E-05 0.000860633 Low-group

KIFC1 1.246212114 3.57E-05 0.000860633 High-group

PLVAP -1.048709734 3.59E-05 0.000863136 Low-group

TTC36 -2.734564992 3.59E-05 0.000863136 Low-group

SLC25A25 -1.239005046 3.60E-05 0.000863136 Low-group

TMPRSS11D -4.521870285 3.60E-05 0.000863136 Low-group

FAM101A 2.515202563 3.61E-05 0.000863136 High-group

TSPAN15 1.284298388 3.61E-05 0.000863136 High-group

NT5DC4 1.904348563 3.68E-05 0.000876078 High-group

NUDT6 -1.322511836 3.69E-05 0.000878015 Low-group

SIAE -1.064683466 3.70E-05 0.000879275 Low-group

ABCC9 -1.651950926 3.73E-05 0.000885081 Low-group

TRIM31 1.827347003 3.77E-05 0.00089434 High-group

RYR2 -2.494892227 3.83E-05 0.000906513 Low-group

MYLK -1.085685921 3.86E-05 0.000913021 Low-group

PCK2 -1.329911456 3.90E-05 0.000921416 Low-group

BANF2 2.169808454 3.94E-05 0.00092894 High-group

BHMT -2.252811147 3.99E-05 0.000938427 Low-group

HSPB2 -2.473064565 4.02E-05 0.000942721 Low-group

TMEM150C -1.312446091 4.03E-05 0.000945271 Low-group

SYTL5 -2.237528842 4.04E-05 0.00094675 Low-group

DLX2 3.575843252 4.05E-05 0.000947063 High-group

SLC10A4 2.851526926 4.06E-05 0.000948734 High-group

PLIN4 -2.085754572 4.10E-05 0.000956636 Low-group

NCAPH 1.276548141 4.15E-05 0.000967973 High-group

DQX1 3.073231315 4.16E-05 0.000968546 High-group

PTN -1.821959939 4.17E-05 0.000968546 Low-group

RNF17 3.51557453 4.19E-05 0.000973593 High-group

SLC25A15 -1.529177039 4.21E-05 0.000976871 Low-group

CRABP2 2.027738754 4.28E-05 0.000989664 High-group

OXTR 2.273081555 4.29E-05 0.000990782 High-group

TECRL -4.628565574 4.30E-05 0.000993067 Low-group

MT1B 8.341867413 4.31E-05 0.000994347 High-group

SLC34A2 3.862861998 4.35E-05 0.001002388 High-group

EDDM3A -7.653464697 4.38E-05 0.001005317 Low-group

PNPO -1.022413044 4.41E-05 0.001011232 Low-group

SOX2 3.906498578 4.42E-05 0.001012768 High-group

RGS22 -3.169620508 4.42E-05 0.001012768 Low-group

FUT9 5.099775533 4.44E-05 0.001015568 High-group

MYCN 2.023392827 4.45E-05 0.001016669 High-group

SEMA3A 2.142489172 4.46E-05 0.00101825 High-group

GABRP -2.871338765 4.49E-05 0.001022205 Low-group

TBC1D26 -5.478812812 4.51E-05 0.001025886 Low-group

PLA2G7 1.522997782 4.51E-05 0.001025886 High-group

DNER 4.680694204 4.52E-05 0.001027554 High-group

CD207 2.287030637 4.56E-05 0.001034253 High-group

MCM10 1.371837946 4.60E-05 0.001042003 High-group

GNRH2 -3.126088132 4.63E-05 0.001046144 Low-group

TIGD2 -1.157042387 4.63E-05 0.001046144 Low-group

MAT1A -1.410389533 4.64E-05 0.001047512 Low-group

SLC52A2 1.062401878 4.65E-05 0.001047512 High-group

ALX1 5.279722537 4.65E-05 0.001047512 High-group

RTP3 -2.024318769 4.67E-05 0.001051504 Low-group

KCNIP2 -2.171066933 4.69E-05 0.001054072 Low-group

KCNJ10 2.262110052 4.70E-05 0.001054072 High-group

ACSM5 -1.848416435 4.70E-05 0.001054072 Low-group

FMO4 -1.242661592 4.71E-05 0.001054072 Low-group

MBOAT4 2.151536926 4.71E-05 0.001054072 High-group

BEND3 1.042814202 4.73E-05 0.00105821 High-group

PHLDA1 -1.328940175 4.75E-05 0.001060243 Low-group

SCP2 -1.239734367 4.77E-05 0.001063122 Low-group

WDR62 1.195429207 4.78E-05 0.001064547 High-group

KLHDC7B 2.275515922 4.80E-05 0.001066463 High-group

PMAIP1 1.556022934 4.82E-05 0.001067313 High-group

EPHX2 -1.205249784 4.82E-05 0.001067313 Low-group

HNF1B 1.291609298 4.92E-05 0.001087877 High-group

EXOC3L2 -1.016679307 4.96E-05 0.001092437 Low-group

PLG -1.622708371 4.98E-05 0.00109508 Low-group

PABPC1L 1.136718464 5.03E-05 0.001105151 High-group

VWF -1.25396226 5.07E-05 0.001110392 Low-group

GSG2 1.437754254 5.07E-05 0.001110392 High-group

TIGD1 1.296548487 5.10E-05 0.001114289 High-group

MTL5 1.28907187 5.12E-05 0.001117489 High-group

FOXI1 5.364861667 5.15E-05 0.001121395 High-group

SLC7A14 -3.336387132 5.18E-05 0.001126745 Low-group

CDK15 -3.02103006 5.24E-05 0.00113598 Low-group

CRYGN -3.716255843 5.27E-05 0.001141724 Low-group

TPX2 1.190222186 5.31E-05 0.00114819 High-group

NHLH2 3.795487065 5.33E-05 0.001150613 High-group

ADAMTS14 1.606980469 5.34E-05 0.001150613 High-group

KCNH3 2.345601095 5.35E-05 0.001152278 High-group

DIRAS3 -2.088311511 5.37E-05 0.001153905 Low-group

RBP4 -1.228422879 5.38E-05 0.001155128 Low-group

DLGAP5 1.368688251 5.39E-05 0.001155128 High-group

C16orf45 -1.298041264 5.43E-05 0.001162527 Low-group

KCNK17 -2.414041699 5.43E-05 0.001162527 Low-group

CLEC17A 3.047373314 5.44E-05 0.001163052 High-group

HPX -1.468998937 5.45E-05 0.001163052 Low-group

PRKG2 3.140365083 5.49E-05 0.001170236 High-group

KIF15 1.463491112 5.54E-05 0.001178421 High-group

CDT1 1.261223099 5.54E-05 0.001178841 High-group

ABCA6 -1.461371303 5.56E-05 0.001180343 Low-group

LIN28A 3.371987625 5.56E-05 0.001180343 High-group

MYH3 -1.832140839 5.60E-05 0.001185523 Low-group

SYT13 3.659860814 5.60E-05 0.001185523 High-group

MAP1A -2.126754507 5.62E-05 0.001188253 Low-group

TSLP -1.948321846 5.65E-05 0.001193399 Low-group

NAAA -1.082202657 5.70E-05 0.001200449 Low-group

MFSD2A -2.666485932 5.70E-05 0.001200449 Low-group

ASPRV1 -1.073980145 5.71E-05 0.001200449 Low-group

C18orf54 1.156519544 5.73E-05 0.001203049 High-group

BIRC5 1.397440482 5.79E-05 0.001213527 High-group

DYNC1I1 -2.443245203 5.85E-05 0.001223519 Low-group

KAAG1 2.286287714 5.91E-05 0.0012363 High-group

AQP5 -2.786436542 5.92E-05 0.001236809 Low-group

PLEKHG4 1.779462027 5.95E-05 0.001241721 High-group

FAM64A 1.728374025 5.96E-05 0.001241831 High-group

ZBTB16 -1.743700369 5.97E-05 0.001242572 Low-group

BCKDHA -1.18582795 5.99E-05 0.001245979 Low-group

ATP8A2 2.074232269 6.00E-05 0.001246001 High-group

ZNF486 1.74752975 6.06E-05 0.001256254 High-group

SAA2 -3.367219268 6.12E-05 0.001266595 Low-group

FABP5 1.20167539 6.13E-05 0.001267844 High-group

PXMP2 -1.210913635 6.14E-05 0.001268968 Low-group

DCXR -1.556918302 6.19E-05 0.001277939 Low-group

SLC16A3 1.315058053 6.26E-05 0.001289872 High-group

PRC1 1.069578729 6.34E-05 0.001305115 High-group

HAND2 -2.492059609 6.38E-05 0.001310986 Low-group

PCDHA6 3.056602866 6.42E-05 0.001312638 High-group

TOR3A 1.017050683 6.43E-05 0.001312638 High-group

IRX6 -4.099818942 6.43E-05 0.001312638 Low-group

RASL10B -2.628167083 6.46E-05 0.00131715 Low-group

LHX4 1.321362435 6.51E-05 0.001325229 High-group

COLEC10 -2.019508896 6.59E-05 0.001338871 Low-group

GRPR -2.748417378 6.59E-05 0.001338871 Low-group

SH2D3A 1.526185391 6.60E-05 0.001338871 High-group

ADRB1 -2.166481015 6.76E-05 0.001363742 Low-group

ADAMTSL3 -1.273375576 6.76E-05 0.001363742 Low-group

ASPM 1.254152408 6.77E-05 0.001364086 High-group

PF4 -2.561705991 6.78E-05 0.001364086 Low-group

CST8 4.199696327 6.79E-05 0.001364086 High-group

TUBE1 -1.062713985 6.79E-05 0.001364086 Low-group

ZNF860 1.872531413 6.80E-05 0.001364086 High-group

DTL 1.219248499 6.84E-05 0.001372263 High-group

RNF125 -1.315423567 6.91E-05 0.001382795 Low-group

PRKCG 2.611698567 6.96E-05 0.001387627 High-group

CCNB2 1.214978858 6.99E-05 0.001391444 High-group

AR -1.697761566 7.03E-05 0.001395504 Low-group

MTRNR2L12 -1.589557948 7.03E-05 0.001395504 Low-group

MAP1LC3A -1.351542289 7.04E-05 0.001395504 Low-group

RAB42 1.516900976 7.09E-05 0.001404714 High-group

KIF14 1.322957827 7.10E-05 0.001405927 High-group

PPFIA4 2.27977131 7.13E-05 0.001409977 High-group

GPD1 -1.639519766 7.18E-05 0.001418833 Low-group

RIBC2 1.690997307 7.20E-05 0.001420562 High-group

CDCA7L 1.188561667 7.27E-05 0.001432272 High-group

HSD17B6 -1.682977195 7.30E-05 0.001436687 Low-group

LAMP5 2.78378829 7.32E-05 0.001440789 High-group

ELN -1.888362671 7.33E-05 0.00144109 Low-group

DAO -1.577233016 7.38E-05 0.001448054 Low-group

ZNF738 1.42370009 7.40E-05 0.001451039 High-group

ITPR2 -1.170147134 7.42E-05 0.001453652 Low-group

CTSK -2.264131465 7.44E-05 0.001456186 Low-group

KRT80 2.576269585 7.46E-05 0.00145779 High-group

LYPD2 -2.716636472 7.47E-05 0.00145779 Low-group

CD177 3.255116984 7.50E-05 0.00146154 High-group

PPP1R9A 1.724074248 7.51E-05 0.001462806 High-group

CYP4F2 -1.868493029 7.59E-05 0.00147727 Low-group

FUT3 2.491487756 7.71E-05 0.001499188 High-group

TES 1.117563745 7.73E-05 0.001500789 High-group

COL23A1 -1.848562569 7.74E-05 0.001500789 Low-group

ANGPTL6 -1.422048546 7.74E-05 0.001500789 Low-group

SOX7 -1.230434053 7.82E-05 0.001514125 Low-group

MTBP 1.013396647 7.83E-05 0.001515611 High-group

SALL1 -1.013299584 7.85E-05 0.001517409 Low-group

DNASE1L3 -1.578033758 7.93E-05 0.001531078 Low-group

B3GALNT1 1.212289344 8.04E-05 0.00154954 High-group

PI15 -2.894082071 8.21E-05 0.001578147 Low-group

KIF26B 1.794545171 8.22E-05 0.001579277 High-group

GPM6A -2.073710303 8.30E-05 0.001591431 Low-group

TRIM46 1.628159146 8.36E-05 0.001601236 High-group

ZBTB12 1.126959244 8.45E-05 0.001616792 High-group

TRIM2 -1.165231847 8.53E-05 0.001628826 Low-group

NMNAT2 -2.614133586 8.57E-05 0.001631649 Low-group

GCNT1 1.67186934 8.59E-05 0.001633421 High-group

FAM124B -1.700778838 8.71E-05 0.001652781 Low-group

SH3GL3 -4.731914114 8.74E-05 0.001655464 Low-group

C18orf42 -3.010738346 8.76E-05 0.001657387 Low-group

KIF3C 1.378100178 8.77E-05 0.00165748 High-group

CARNS1 1.990870663 8.79E-05 0.001657592 High-group

SULT2A1 -1.645514488 8.80E-05 0.001659284 Low-group

BARD1 1.018812414 8.86E-05 0.001667173 High-group

EMP1 -1.208008457 8.86E-05 0.001667173 Low-group

CXorf67 3.901012895 8.89E-05 0.001670068 High-group

BEND4 2.985725899 8.92E-05 0.001673872 High-group

MT-ATP8 -1.122221222 8.94E-05 0.001673964 Low-group

ARHGAP40 3.020981225 8.94E-05 0.001673964 High-group

ERCC6L 1.337282107 8.98E-05 0.001678765 High-group

B3GNT5 1.533762964 9.10E-05 0.001696781 High-group

STRA8 2.5879351 9.10E-05 0.001696781 High-group

RAVER2 1.366259376 9.11E-05 0.001697341 High-group

PCSK6 -1.366846765 9.16E-05 0.001704263 Low-group

CSPP1 1.015234796 9.32E-05 0.001726139 High-group

GATSL3 -1.101899201 9.33E-05 0.001726139 Low-group

SALL4 2.214976218 9.36E-05 0.001731169 High-group

MROH2B 3.537038952 9.48E-05 0.001745669 High-group

CST5 3.45078768 9.48E-05 0.001745669 High-group

RGS11 -2.147226423 9.48E-05 0.001745669 Low-group

HBM -4.223861069 9.52E-05 0.001750292 Low-group

PTK7 1.615248577 9.53E-05 0.001750292 High-group

SAGE1 4.415721957 9.62E-05 0.001766052 High-group

PBLD -1.299711088 9.71E-05 0.001780604 Low-group

SCN4B -1.274141101 9.72E-05 0.001780604 Low-group

FAM72C 1.747432153 9.78E-05 0.001789116 High-group

ST6GALNAC5 2.481207155 9.79E-05 0.001790071 High-group

FHOD3 2.165982718 9.81E-05 0.001792981 High-group

DCHS2 2.478776657 9.89E-05 0.001804999 High-group

MYCL -1.32614018 0.000100032 0.001822391 Low-group

SMR3B -6.360893179 0.000100216 0.001824066 Low-group

WBSCR17 -3.132346332 0.000100416 0.001826022 Low-group

STAP1 2.341293378 0.000101313 0.00183917 High-group

SYBU -1.272039867 0.000101326 0.00183917 Low-group

LILRB5 -1.199506642 0.000101994 0.001848081 Low-group

FBXO2 -1.761763735 0.000102004 0.001848081 Low-group

PCDHA10 2.418633781 0.000102101 0.001848129 High-group

AZGP1 -1.480050027 0.000102477 0.001853238 Low-group

KCNH2 3.043602287 0.000102882 0.001858854 High-group

PRAMEF7 4.750118781 0.000103422 0.001866899 High-group

C3orf36 -1.944891617 0.000103893 0.001870883 Low-group

CLGN 1.961977694 0.000103952 0.001870883 High-group

DEPDC1 1.496034542 0.000104 0.001870883 High-group

P2RX5 1.745528719 0.000104022 0.001870883 High-group

AATK 1.672135763 0.000104168 0.001871794 High-group

FAM216B 5.029273621 0.000104645 0.001878001 High-group

TRPC4 -1.869423843 0.000104704 0.001878001 Low-group

CT83 5.479977585 0.000105436 0.001889416 High-group

LPAR2 1.301947033 0.000105894 0.001895896 High-group

NEURL1 1.679879868 0.000106636 0.001905725 High-group

ADH1A -1.817545966 0.000107363 0.001916969 Low-group

MELK 1.248382726 0.000107795 0.001922944 High-group

AP1G2 1.003508057 0.00010802 0.001925224 High-group

CLLU1OS 3.136844823 0.000108772 0.001935119 High-group

ATP2C2 2.479144231 0.000109063 0.001938543 High-group

RCAN3 1.13118385 0.000109182 0.001938919 High-group

NEIL3 1.55490892 0.000109607 0.001943297 High-group

PAEP 4.127129712 0.000109626 0.001943297 High-group

C16orf96 -2.074134992 0.000110774 0.001960119 Low-group

CD70 -2.392721615 0.000110876 0.001960169 Low-group

PLSCR5 6.754067035 0.000112092 0.00197812 High-group

HIST2H2BF 1.626935608 0.000112263 0.001979356 High-group

JPH3 3.011906977 0.00011302 0.001990937 High-group

TRPC7 2.607343845 0.000113201 0.00199233 High-group

MS4A2 -2.404360985 0.000114201 0.002008147 Low-group

TRIM35 -1.006203739 0.000114471 0.002011093 Low-group

PNMA5 -3.349169018 0.00011475 0.002014211 Low-group

CCDC158 -1.456797192 0.000114959 0.002016082 Low-group

ZDHHC13 1.148616247 0.000115762 0.002026551 High-group

ALDOB -1.792801893 0.000116634 0.002040004 Low-group

CHAF1B 1.311403072 0.000117037 0.002041898 High-group

S100A8 2.289023395 0.000117053 0.002041898 High-group

MTCL1 1.470654679 0.000117189 0.002042465 High-group

CDC45 1.211170687 0.000118392 0.00206104 High-group

HN1 1.058694512 0.000118464 0.00206104 High-group

ITPR3 1.734013261 0.000118983 0.002068248 High-group

CCBE1 -2.089685957 0.000119979 0.002081886 Low-group

PCDHA4 2.44129368 0.000120285 0.002084375 High-group

MASP1 -1.190385182 0.000120334 0.002084375 Low-group

SPATA17 2.169957115 0.000120486 0.002085177 High-group

CT45A10 5.1708965 0.000120824 0.002087738 High-group

IGF1R 1.600939324 0.000120846 0.002087738 High-group

SLFN13 1.932508993 0.000120997 0.002088525 High-group

DUOX1 1.487407625 0.000121469 0.002091182 High-group

COX6B2 2.770626001 0.000122235 0.002102519 High-group

CCDC60 -3.38388501 0.000125171 0.002151154 Low-group

SPC25 1.241434216 0.000125965 0.002162058 High-group

IDO2 -2.6383781 0.000126229 0.00216261 Low-group

ALDH8A1 -1.389756481 0.000126318 0.00216261 Low-group

CENPL 1.067864256 0.000126386 0.00216261 High-group

FAT4 -1.436856268 0.000126874 0.002169068 Low-group

SPINK5 -1.988948946 0.000127337 0.002173661 Low-group

GPT2 -1.198171004 0.000127363 0.002173661 Low-group

SUN3 3.272953593 0.000127587 0.002175606 High-group

TEX43 -2.039604308 0.000129286 0.002200764 Low-group

AOX1 -1.636967086 0.000130729 0.002223411 Low-group

CPA3 -2.248962279 0.000131399 0.002231318 Low-group

TST -1.058031853 0.00013142 0.002231318 Low-group

MAPK8IP1 -1.045543932 0.000131572 0.00223197 Low-group

MAD2L1 1.14362749 0.000131706 0.002232324 High-group

MX2 1.339494819 0.000133247 0.002254559 High-group

ECHDC2 -1.000110666 0.000133805 0.00226206 Low-group

CARD14 1.51697743 0.000135399 0.002283128 High-group

CD24 1.693454994 0.000135534 0.002283455 High-group

EME1 1.169856501 0.000136183 0.002291451 High-group

SLC4A1 -2.755183043 0.000136241 0.002291451 Low-group

TYMS 1.162885276 0.000137173 0.002301745 High-group

CENPI 1.296787883 0.000137175 0.002301745 High-group

PADI2 -2.462319499 0.000137203 0.002301745 Low-group

PRKCE -1.131465517 0.00013815 0.002313693 Low-group

TOP2A 1.326272778 0.00013982 0.002337688 High-group

CNTNAP5 3.288508174 0.000142447 0.002379586 High-group

AFF2 3.106452589 0.000144861 0.00241378 High-group

HAAO -1.143310567 0.000145243 0.002417936 Low-group

SH3YL1 1.524132214 0.000145356 0.002417936 High-group

SLITRK4 1.857712491 0.000146248 0.002429328 High-group

KLK11 4.777244085 0.000146287 0.002429328 High-group

TMEM51 1.27302024 0.000147513 0.002447622 High-group

MTRNR2L6 -1.645152815 0.000148913 0.002465786 Low-group

TUBB3 1.984951367 0.000149034 0.002465786 High-group

EMCN -1.039675301 0.000149444 0.002469264 Low-group

HEY2 -1.200705948 0.000151005 0.002492969 Low-group

IGFBP5 -1.783120466 0.000151318 0.002495107 Low-group

CAT -1.014903602 0.000151387 0.002495107 Low-group

SLC4A4 -1.427171258 0.000151539 0.002495527 Low-group

FBLN2 -1.968549187 0.000153294 0.002520204 Low-group

AKAP3 -1.432361242 0.000153758 0.002525731 Low-group

FCER2 2.244165464 0.000155557 0.002551039 High-group

SMCO3 -1.63928739 0.000157766 0.002585112 Low-group

SGOL1 1.23535548 0.000160572 0.002624539 High-group

MAMDC2 -1.743495166 0.000161733 0.002637482 Low-group

PTTG1 1.358119134 0.000161765 0.002637482 High-group

PROM1 3.062034525 0.000163555 0.002664459 High-group

STEAP3 -1.238277667 0.000163794 0.002666153 Low-group

AEBP1 -2.072398279 0.000163958 0.00266661 Low-group

FAM117B 1.146760586 0.000166022 0.002691297 High-group

SKOR1 -1.264528679 0.000166634 0.002698648 Low-group

SLC9B2 -1.008724967 0.000166749 0.002698648 Low-group

MOGAT1 -1.779560485 0.000167025 0.002700905 Low-group

CLEC1B -2.921922543 0.000167644 0.002708687 Low-group

CDC7 1.181727243 0.000167824 0.002709375 High-group

BPIFA2 3.733073986 0.000168661 0.002720652 High-group

SLC27A2 -1.496746377 0.000168849 0.002721465 Low-group

LYZ 1.939458043 0.000169136 0.002723861 High-group

NTSR2 -5.02779452 0.000170098 0.002735502 Low-group

CENPE 1.171799083 0.000171095 0.002746442 High-group

SORCS1 3.468196134 0.000171487 0.002750496 High-group

TAL1 -1.187835308 0.000172474 0.002761825 Low-group

FOXM1 1.219938479 0.000172683 0.00276293 High-group

CRYAA -2.305143099 0.000172832 0.002763063 Low-group

GSG1 -3.703826831 0.000174946 0.002792339 Low-group

VDR 1.493538769 0.000175553 0.00279975 High-group

3-Mar 1.215664326 0.000175879 0.002802689 High-group

RP13-672B3.2 -1.487963994 0.00017641 0.002808878 Low-group

SUSD2 -1.615668619 0.000176867 0.002813879 Low-group

EPCAM 2.77919571 0.000177208 0.002817036 High-group

NTRK3 -2.133472565 0.000178619 0.002835962 Low-group

DNMT3L -2.178799305 0.00017883 0.002835962 Low-group

COL4A3 -1.935848595 0.000178904 0.002835962 Low-group

TYRP1 -2.998579396 0.000178974 0.002835962 Low-group

DUOX2 3.623380161 0.00017923 0.002837739 High-group

ADH6 -1.434483266 0.000179773 0.002842485 Low-group

ENTPD2 1.348064303 0.000179818 0.002842485 High-group

RGAG4 -1.467407216 0.000180339 0.002848429 Low-group

PLAC1 2.686876351 0.0001805 0.002848699 High-group

WDHD1 1.072241966 0.000181428 0.002860383 High-group

FTCDNL1 -1.020455624 0.000181942 0.002863185 Low-group

FABP6 3.213685918 0.000182413 0.002864907 High-group

WDPCP -1.037870138 0.000182564 0.002864907 Low-group

AQP10 3.428718907 0.00018269 0.002864907 High-group

HSF2BP 1.160559928 0.000182939 0.002866536 High-group

FAM72A 1.239228674 0.000183749 0.002874654 High-group

LIPJ -1.888028661 0.000184822 0.002889144 Low-group

CAPN13 2.906948802 0.000185984 0.002905006 High-group

ACAD11 -1.398142304 0.000188421 0.002939794 Low-group

ESYT3 1.765506965 0.000190501 0.002968499 High-group

TMEM252 -2.666441001 0.000191245 0.002977747 Low-group

ABCB11 -2.257430525 0.000193091 0.00299903 Low-group

RAP1GAP2 1.486761987 0.000193221 0.00299903 High-group

ETV5 1.073252929 0.000198907 0.003072763 High-group

GPR62 -1.787080649 0.000200485 0.00309472 Low-group

LPL -1.612325422 0.000201002 0.003100271 Low-group

PBK 1.312283154 0.000201916 0.003111941 High-group

ABO -1.910101915 0.000202288 0.003115239 Low-group

SCGB1D2 3.645745599 0.000203031 0.003124232 High-group

ABCA8 -1.908771685 0.000203716 0.003128083 Low-group

DSC2 1.139874867 0.0002039 0.003128083 High-group

C2orf82 1.918903866 0.000203916 0.003128083 High-group

TRH 4.589706069 0.000204507 0.00313471 High-group

CBLN3 -1.315442706 0.000204787 0.003136563 Low-group

NOX3 3.335213559 0.000205858 0.003150522 High-group

IL12A 1.356491788 0.000207849 0.003170397 High-group

KIF21B 1.314436766 0.000208641 0.003178305 High-group

AC006547.14 -1.200459915 0.000209073 0.003181018 Low-group

PCDHA3 2.623076271 0.000209616 0.00318578 High-group

ZNF432 1.053781667 0.000210286 0.003193501 High-group

ITGAE 1.298955492 0.00021047 0.003193829 High-group

ABHD2 -1.001178623 0.000211606 0.003206135 Low-group

GCGR -2.408781844 0.000212145 0.003209359 Low-group

POU3F2 2.468552254 0.000212847 0.003215056 High-group

SLC5A11 2.103660151 0.000213277 0.003219085 High-group

PPP1R32 -1.084318831 0.000214059 0.003227764 Low-group

EFNA5 2.35274724 0.000214539 0.003230699 High-group

NPHS2 4.600564923 0.000214847 0.003232868 High-group

DEFA5 6.152181635 0.000216373 0.003250877 High-group

EGLN3 1.661623345 0.000216764 0.00325426 High-group

ADRA2B -1.128851396 0.00021701 0.003255477 Low-group

ARMS2 -2.110421355 0.000217243 0.003256499 Low-group

DSCAML1 2.248880553 0.000217659 0.003260248 High-group

RAD51AP1 1.167359748 0.000217933 0.003261878 High-group

RP11-307N16.6 -2.08627392 0.000218137 0.003262448 Low-group

TCP11 -1.891650551 0.000219007 0.003272979 Low-group

KIAA2022 2.325875884 0.000219256 0.003274227 High-group

RND2 2.135082124 0.0002208 0.00328981 High-group

NR4A3 -1.543713776 0.000222316 0.003309614 Low-group

GPD1L 1.085947878 0.000222701 0.003310621 High-group

EDDM3B -4.960329472 0.000224007 0.003320481 Low-group

PNPLA7 -1.146740816 0.000224323 0.003320481 Low-group

PTF1A 3.415288286 0.000224468 0.003320481 High-group

AGR2 3.237355568 0.000224543 0.003320481 High-group

THRSP -2.810770242 0.00022561 0.003331257 Low-group

ZNF714 1.58312075 0.000229311 0.003380842 High-group

MAPK15 2.084438291 0.000230078 0.003389604 High-group

CAPN8 2.00439074 0.000230489 0.003393133 High-group

CLIC3 1.754369895 0.000231267 0.003402042 High-group

ENAH 1.070262366 0.000237649 0.003482928 High-group

TENM1 -2.252595025 0.000239273 0.003504127 Low-group

HGD -1.248105589 0.000241321 0.003531488 Low-group

NLGN4X -2.149698491 0.000242037 0.003539346 Low-group

MAMSTR 1.512277947 0.00024274 0.00354413 High-group

TRPM3 -2.25501166 0.000242904 0.00354413 Low-group

RMI2 1.168775449 0.000243337 0.003547817 High-group

IGFL2 2.448147629 0.000244965 0.003563659 High-group

WDR76 1.101096641 0.000245707 0.003568216 High-group

FAM153B 2.420760996 0.000245822 0.003568216 High-group

ACSBG1 -2.067132394 0.000246006 0.003568265 Low-group

STEAP4 -1.554492806 0.000246596 0.003574185 Low-group

SNTA1 -1.403069337 0.000246937 0.003576509 Low-group

MKI67 1.191364877 0.000248895 0.003600539 High-group

CCND2 -1.684425989 0.000252326 0.003641175 Low-group

ENPP6 -2.705622484 0.000253188 0.00365094 Low-group

ADARB2 2.293611174 0.000253384 0.003651101 High-group

CHL1 -3.182489529 0.000254207 0.003660285 Low-group

SERPING1 -1.095184629 0.000254654 0.003663156 Low-group

FIGF -2.092055584 0.000254778 0.003663156 Low-group

KCNS2 -2.69887226 0.000255104 0.003664311 Low-group

KDELR3 1.086097628 0.00025523 0.003664311 High-group

PSMC3IP 1.001385426 0.000259405 0.003713429 High-group

KCND3 -1.769776951 0.000261915 0.003741204 Low-group

CFP -1.330388136 0.000262229 0.003742982 Low-group

ZNF320 1.354125402 0.000263582 0.003759572 High-group

CREG2 1.65816026 0.000265318 0.003781601 High-group

KLHDC8A 1.852190435 0.00026704 0.003800299 High-group

NCR1 -1.716274747 0.000267209 0.003800299 Low-group

ACAN 1.825570551 0.000269796 0.003831575 High-group

GOLGA6B -2.894778173 0.000271367 0.003847328 Low-group

HRSP12 -1.195305129 0.000273015 0.003863362 Low-group

TMEM35 2.18840109 0.000276947 0.003913384 High-group

CA3 -1.8506138 0.000277527 0.003918782 Low-group

TRAF5 1.090260637 0.000277902 0.003920857 High-group

GRB7 1.078544851 0.000278072 0.003920857 High-group

CHST5 -3.371124719 0.000278459 0.003923504 Low-group

ZNF665 1.505148986 0.000278682 0.003923847 High-group

NUDT17 1.066955457 0.000280544 0.003947241 High-group

SFRP1 -2.007438666 0.000281233 0.003947569 Low-group

PPP1R14D 2.193845395 0.000281279 0.003947569 High-group

HBD -3.182023674 0.000281368 0.003947569 Low-group

GPSM2 1.051336831 0.000281953 0.003952956 High-group

PPP1R1B 2.859691289 0.000283168 0.003967172 High-group

CDCA3 1.109931536 0.000283867 0.003974132 High-group

CFHR4 -1.793357531 0.00028467 0.0039798 Low-group

CDKN3 1.29179013 0.000284754 0.0039798 High-group

SLCO1B3 -2.966450481 0.000284895 0.0039798 Low-group

CTHRC1 1.610512789 0.000285079 0.0039798 High-group

DDIT3 1.071816629 0.00028657 0.003995663 High-group

TRIM6 1.461652159 0.000286621 0.003995663 High-group

GALNT14 1.854127875 0.000288199 0.004011997 High-group

GFRA1 -1.795841028 0.000288567 0.004014281 Low-group

SCARA3 1.469325574 0.000290096 0.004032708 High-group

PPL -1.13947956 0.000290996 0.004042366 Low-group

SCGB2A2 3.186924101 0.000292127 0.004052386 High-group

AICDA 3.218197683 0.000292781 0.004058576 High-group

ZIM2 2.500434825 0.00029501 0.00408304 High-group

CAMK2A -2.35676082 0.000295167 0.00408304 Low-group

HIST1H1E 1.307515347 0.000295412 0.004083568 High-group

TSPYL5 -1.824756623 0.000296725 0.004098842 Low-group

SLC2A1 1.225339414 0.000297822 0.004111111 High-group

NPDC1 -1.02091062 0.000298101 0.004112079 Low-group

RP11-1220K2.2 3.174983706 0.000299656 0.004130648 High-group

MYOM2 -1.812573523 0.000300055 0.004131216 Low-group

LHX8 5.258515165 0.000300117 0.004131216 High-group

MROH7 -2.082289955 0.000301319 0.004144872 Low-group

C1QTNF1 -2.000111657 0.000303497 0.004171922 Low-group

XDH -1.477203291 0.000304538 0.004183308 Low-group

TRAIP 1.079249204 0.000306916 0.004208725 High-group

KIAA0319 1.518035106 0.000307029 0.004208725 High-group

CDC42BPG 1.508395905 0.000307638 0.004214149 High-group

TBX3 -1.498864137 0.000307946 0.004215442 Low-group

MT-ND3 -1.049476478 0.000309101 0.004227852 Low-group

HSD17B13 -2.593579682 0.000309282 0.004227852 Low-group

C1orf94 3.96587049 0.000316825 0.004318983 High-group

GPR110 -3.000709637 0.000325017 0.004417218 Low-group

FAM72B 1.333761596 0.000325151 0.004417218 High-group

HORMAD2 -2.421673204 0.000325741 0.00442218 Low-group

MUC20 1.668959897 0.000327132 0.004438006 High-group

MYRF 1.101964683 0.000327589 0.004441146 High-group

FAM182B 1.459264927 0.000328016 0.00444239 High-group

ELOVL7 1.89713327 0.000328131 0.00444239 High-group

SSTR3 2.815665141 0.000330777 0.004472074 High-group

FOSL1 -1.530145129 0.00033112 0.004473635 Low-group

ISL1 3.947116932 0.000331484 0.004475494 High-group

KAZN -1.856936166 0.000334535 0.0045105 Low-group

SKA1 1.374818098 0.000335267 0.004513074 High-group

CHRNA7 1.936721558 0.000335431 0.004513074 High-group

IGF2 2.832125016 0.000335642 0.004513074 High-group

POLQ 1.124629987 0.000341112 0.004570131 High-group

ZNF165 1.211873889 0.000341276 0.004570131 High-group

FKBP10 1.398035432 0.000342544 0.004581472 High-group

DUXA -5.350020262 0.000342588 0.004581472 Low-group

ADH4 -2.262644515 0.000346018 0.00461795 Low-group

FCRL4 3.515850062 0.000346378 0.004619622 High-group

BOP1 1.122003205 0.000348205 0.004637717 High-group

SPINK13 2.294391935 0.000353672 0.004695747 High-group

PCP4 -4.596093316 0.000353991 0.004695747 Low-group

LEFTY1 2.192257188 0.000355373 0.004707738 High-group

NUP210L -1.953663347 0.00035744 0.004731933 Low-group

FMO3 -1.610834513 0.000358144 0.004738071 Low-group

CES4A -1.621021748 0.000360244 0.004759471 Low-group

CENPM 1.288696529 0.0003618 0.004773338 High-group

RP11-505K9.4 -1.208407149 0.000362504 0.004773338 Low-group

14-Sep 3.927443573 0.000363143 0.004775364 High-group

OLFM2 -1.176302893 0.000364172 0.004785705 Low-group

RIMKLA 1.813657178 0.000370405 0.004864367 High-group

IL2RG 1.223380432 0.000370727 0.004865365 High-group

ZFR2 2.511428266 0.000371521 0.004872539 High-group

AFF3 1.36236835 0.00037254 0.00488265 High-group

ABCA12 1.846721419 0.000375574 0.004919146 High-group

ETNPPL -1.86726839 0.00037969 0.004956594 Low-group

PCDHA7 2.508320409 0.000380774 0.00496745 High-group

PLXNA4 -2.714417104 0.000384154 0.005004916 Low-group

ABCA9 -1.392143918 0.000385001 0.005010015 Low-group

ZC2HC1A 1.266839434 0.000387957 0.005038063 High-group

TMC7 1.102124057 0.000387976 0.005038063 High-group

DIAPH3 1.167249402 0.000391234 0.005065644 High-group

TREML4 3.106469444 0.000391335 0.005065644 High-group

ANG -1.387825274 0.000391385 0.005065644 Low-group

MCF2L2 1.743623701 0.000393244 0.005081163 High-group

RDH5 -1.224505457 0.000393357 0.005081163 Low-group

VIP 1.93919142 0.000394005 0.005086195 High-group

RHOXF2B 5.501481145 0.000398722 0.005133639 High-group

SCN4A -1.165418057 0.00040155 0.005166674 Low-group

PPP1R14C 2.48955432 0.000402747 0.005171951 High-group

FAS -1.063684309 0.000406349 0.005211414 Low-group

FZD9 -2.633071991 0.000407497 0.005220181 Low-group

PRR15L 1.822872563 0.000407562 0.005220181 High-group

BDH2 -1.028006421 0.000411969 0.00526978 Low-group

ARHGEF16 1.361590622 0.000413575 0.005283472 High-group

ZSCAN5B -2.283868484 0.000414116 0.005286952 Low-group

TMED3 1.240229674 0.000414981 0.005294564 High-group

SLC18A2 -1.751325913 0.000415441 0.005297002 Low-group

NAP1L5 -1.088834942 0.000418877 0.005324823 Low-group

ZNF761 1.033323986 0.000419267 0.005324823 High-group

PCDH20 -2.513493936 0.000420229 0.005330473 Low-group

CPN2 -1.209636591 0.00042143 0.005342274 Low-group

INSIG1 -1.416337758 0.000421942 0.00534532 Low-group

WBP5 1.160274219 0.000422543 0.005349501 High-group

OR13A1 3.390164227 0.000423345 0.005356209 High-group

ALOX5 1.304058203 0.000426641 0.005390989 High-group

MMP12 2.327733148 0.000429417 0.00541565 High-group

SERPINE2 1.662501751 0.000431048 0.005432746 High-group

ZIC5 2.619744023 0.000435091 0.005469716 High-group

AASS -1.68767766 0.00043727 0.005493603 Low-group

ATP11C -1.152291999 0.000440098 0.005525616 Low-group

CLDN8 -3.299564361 0.000442739 0.005548021 Low-group

CDO1 -1.4232013 0.000442748 0.005548021 Low-group

DGKG 1.357662907 0.000443009 0.005548021 High-group

SLC2A2 -1.431119774 0.000446626 0.00558623 Low-group

SGPP2 2.161562141 0.000449289 0.005612414 High-group

KCTD19 2.16507276 0.000455518 0.005675839 High-group

MAGI2 -1.011425896 0.000455936 0.005675839 Low-group

PLXNB3 1.741239787 0.000456066 0.005675839 High-group

UGT2B15 -1.758876169 0.000457874 0.005694395 Low-group

APBA1 -1.530185507 0.000463993 0.005755968 Low-group

ZNF525 1.346526889 0.000464535 0.005759068 High-group

FOXI3 2.978576379 0.000471057 0.005828927 High-group

ASTN2 -1.160708683 0.000475813 0.005873032 Low-group

CYP2E1 -2.388603057 0.000476995 0.005883928 Low-group

GPR182 -1.738409387 0.000479281 0.005908435 Low-group

CAPN6 3.111947899 0.000480593 0.005920906 High-group

LCN6 -1.822535275 0.000482411 0.00593589 Low-group

ATOH7 -1.431796432 0.000485177 0.005966198 Low-group

LGALS7B -3.27197221 0.000485712 0.005969049 Low-group

SH2D7 2.026150933 0.00048607 0.005969722 High-group

IL17REL 2.498383759 0.000488587 0.005996901 High-group

EREG 2.397052224 0.000490933 0.006018199 High-group

CCRN4L -1.087476033 0.000495614 0.006071814 Low-group

LMO3 -2.072960072 0.000496934 0.006084202 Low-group

SLC39A8 -1.004554454 0.000497481 0.006087121 Low-group

ACTBL2 3.188996656 0.000499934 0.006105755 High-group

ELFN1 -1.733586397 0.000500929 0.006110341 Low-group

NEFM 3.031206138 0.000501486 0.006113355 High-group

PADI3 -2.968334282 0.000503131 0.006129619 Low-group

DAK -1.176594294 0.000505072 0.006145672 Low-group

IGSF3 1.31426829 0.000506491 0.006159135 High-group

PYCARD 1.391432612 0.000509324 0.00618976 High-group

IYD -1.573300993 0.000511875 0.006209276 Low-group

ACSS3 -1.462259516 0.000512579 0.006213998 Low-group

SERPINA12 2.301519957 0.000513103 0.006216122 High-group

C11orf45 1.202264142 0.000514531 0.006226168 High-group

NT5DC2 1.133675441 0.000515637 0.006235734 High-group

MTRNR2L10 -1.445755431 0.000518213 0.006262414 Low-group

PSORS1C2 2.837776001 0.000518479 0.006262414 High-group

NAT2 -2.114578626 0.000522181 0.006295552 Low-group

SYT2 -1.773278299 0.000527645 0.006349775 Low-group

C15orf43 -2.331609618 0.000529734 0.006371029 Low-group

SEC31B -1.144829309 0.000532276 0.006393801 Low-group

SERPINB4 5.27176894 0.000532731 0.006395372 High-group

TUBB4A -2.432810962 0.000535891 0.006417668 Low-group

SGSM1 1.438473656 0.00053656 0.006421355 High-group

CCL21 2.402785987 0.000542135 0.006476709 High-group

RAET1L 2.924105758 0.000544721 0.006503657 High-group

FOSB -1.850275798 0.000548885 0.006549407 Low-group

AMER3 3.706465555 0.000549531 0.006553151 High-group

OBSCN 1.632742394 0.000550251 0.006557766 High-group

F9 -1.827166363 0.000552428 0.006579734 Low-group

NXF5 -2.218361643 0.000555079 0.006599342 Low-group

PHYHIP -1.671376756 0.000558361 0.006626375 Low-group

GDPD3 1.295237442 0.000558819 0.006627828 High-group

ADORA2B 1.325045422 0.00056114 0.006647351 High-group

ASF1B 1.039106854 0.00056228 0.006653829 High-group

CXCR5 3.446014379 0.000563818 0.006658583 High-group

NOL4 -2.978206555 0.000563855 0.006658583 Low-group

PRR23B 4.932939807 0.000564115 0.006658583 High-group

GPBAR1 -1.647877032 0.000564464 0.006658713 Low-group

ZNF675 1.009931526 0.00056692 0.006683691 High-group

IGSF23 -1.423833206 0.000568884 0.006702838 Low-group

GPR125 -1.161288701 0.000569501 0.006706094 Low-group

C2orf54 1.801103665 0.000572125 0.006730559 High-group

GDAP1L1 -3.173395122 0.000572261 0.006730559 Low-group

FER1L6 3.123701877 0.000576632 0.006777922 High-group

MTMR7 1.566195912 0.000579523 0.006803782 High-group

TTK 1.227821546 0.000580384 0.006807839 High-group

GPC2 1.169470623 0.000581936 0.00681991 High-group

C3orf52 1.921199955 0.00058228 0.00681991 High-group

C1orf145 1.701870781 0.000586052 0.006860014 High-group

NLRP4 -3.395804584 0.00059083 0.006907735 Low-group

ADAP1 1.671222254 0.000596954 0.006959695 High-group

BIRC3 1.394529079 0.00059704 0.006959695 High-group

KRTAP20-4 4.057089023 0.000598109 0.006960052 High-group

KCNK12 -3.300329526 0.000600494 0.006977432 Low-group

HUS1B 1.214215264 0.000601646 0.006984476 High-group

SLC1A1 -1.641977474 0.00060379 0.007005246 Low-group

PYCR1 1.688418261 0.000610176 0.007062706 High-group

DUSP9 2.070498194 0.000610759 0.007065309 High-group

HOXB9 2.87882588 0.000614319 0.007102319 High-group

DUSP13 2.779348533 0.0006147 0.007102556 High-group

ABCA4 1.5001049 0.000618379 0.007136707 High-group

EPS8L3 1.744637171 0.000619789 0.007144606 High-group

RP4-583P15.15 -1.226374851 0.000622167 0.007163575 Low-group

FUT7 1.325385038 0.000625126 0.007189309 High-group

DMRT1 3.176048613 0.000625608 0.007190662 High-group

CTSC 1.019212806 0.000626079 0.007191879 High-group

FAM181A 4.540027846 0.000626938 0.00719756 High-group

TRPM1 -2.172677729 0.000630991 0.007239864 Low-group

TAT -2.015735942 0.000631483 0.007241298 Low-group

FBN2 1.700660845 0.000632713 0.007250606 High-group

SLC9A4 3.21200291 0.000634252 0.007257265 High-group

E2F2 1.06841203 0.00063816 0.007292413 High-group

PSRC1 1.032045257 0.000638808 0.007293682 High-group

ARHGAP19-SLIT1 3.160815946 0.000639877 0.007294892 High-group

FBP2 -1.647350741 0.000640227 0.007294892 Low-group

PIF1 1.062021596 0.000642909 0.007321216 High-group

ZNF816 1.200051735 0.000645377 0.007340838 High-group

EPHA6 3.239132352 0.000646767 0.0073524 High-group

BMF 1.138672704 0.000647303 0.007354256 High-group

ANGPTL1 -1.52664711 0.000648089 0.007356291 Low-group

SLC31A2 -1.130443346 0.000648228 0.007356291 Low-group

CDH9 4.910177036 0.00064943 0.00736379 High-group

UGT2B10 -1.633914523 0.00065001 0.00736379 Low-group

C1S -1.084543535 0.000659596 0.007446709 Low-group

TMIE 1.592264958 0.000660675 0.007454625 High-group

EFHC2 2.471838271 0.000661908 0.007464259 High-group

GPC3 1.904933979 0.000662967 0.007465076 High-group

MLIP -1.502466549 0.000662975 0.007465076 Low-group

ZNF695 2.239365192 0.000666035 0.007489378 High-group

GRIA4 2.948885754 0.000666623 0.007491713 High-group

APOLD1 -1.011823576 0.000669259 0.007516384 Low-group

CCR7 1.767093968 0.000675103 0.00756976 High-group

WIPF3 1.642867103 0.00067571 0.007572249 High-group

SPAG6 2.218886857 0.00067671 0.007578586 High-group

KBTBD12 3.140568065 0.000677362 0.007578586 High-group

APOC3 -1.35723018 0.000677428 0.007578586 Low-group

CDH17 2.557468666 0.000678237 0.007582327 High-group

MAMDC4 -1.697415641 0.00067957 0.007589627 Low-group

OSBP2 1.113380607 0.000682707 0.007603279 High-group

PROZ -1.459772038 0.000682778 0.007603279 Low-group

CD14 -1.151200723 0.000683107 0.007603279 Low-group

GAPT 1.63041983 0.00068501 0.007620157 High-group

CPA2 3.233587288 0.000686765 0.007631067 High-group

ZNF285 1.310451974 0.000689591 0.007656536 High-group

FAM186B -1.069955724 0.000689976 0.007656536 Low-group

ALPK3 1.325015367 0.000690222 0.007656536 High-group

ACCSL -3.584989623 0.000694439 0.007694653 Low-group

ADCY1 -2.043593953 0.000701182 0.00775628 Low-group

CD1D -1.255079688 0.000702875 0.007770648 Low-group

FAM150B 2.450492841 0.000703299 0.007770979 High-group

OVOL1 2.547239286 0.000709297 0.007817008 High-group

NKX2-2 -3.110581396 0.000710863 0.007828235 Low-group

WNT9A 2.04544173 0.000712853 0.007833406 High-group

THSD1 -1.171867356 0.000713417 0.007833406 Low-group

SLC28A3 2.52503906 0.000713717 0.007833406 High-group

C16orf59 1.129908264 0.00071578 0.007851669 High-group

REEP2 1.485143663 0.000716218 0.007852108 High-group

ACPP 1.907594582 0.000718375 0.007862633 High-group

ASPHD2 1.144822713 0.000719032 0.00786496 High-group

COL5A3 -1.242401411 0.000719385 0.00786496 Low-group

FCRL3 2.157662124 0.000721575 0.007880155 High-group

FAM19A1 -1.818328148 0.000725303 0.007908453 Low-group

RP11-77K12.7 -3.481265631 0.00072537 0.007908453 Low-group

ANKS6 1.368591878 0.000726946 0.007921259 High-group

KRTCAP3 1.809517727 0.000732107 0.00796429 High-group

PTCHD4 -1.794563202 0.000733522 0.007970881 Low-group

PLEKHD1 2.158453545 0.000735686 0.007984567 High-group

KRBOX1 -2.132870924 0.000743522 0.008052919 Low-group

FZD7 1.167948435 0.000744406 0.008053634 High-group

C9orf173 -1.309463915 0.000746399 0.008070774 Low-group

SGCG -2.893063075 0.000747189 0.008074887 Low-group

CREB5 1.249405116 0.000750466 0.008101414 High-group

CIDEB -1.397184351 0.000755706 0.008142866 Low-group

GPR146 -1.134228611 0.000755779 0.008142866 Low-group

SLC28A1 -1.560276165 0.000755958 0.008142866 Low-group

TAS1R1 -1.587229239 0.000756637 0.008145725 Low-group

CKAP2L 1.130524825 0.000759232 0.008169202 High-group

SULT4A1 -3.266778704 0.000760406 0.008177374 Low-group

TLR4 -1.063721212 0.000768147 0.008247124 Low-group

GP9 -2.295788391 0.000770662 0.008268096 Low-group

CETP -1.342791785 0.000773822 0.008281157 Low-group

ZNF66 1.473360439 0.000773838 0.008281157 High-group

USH2A -1.490936438 0.000788676 0.008412545 Low-group

PAX9 2.286398087 0.000796393 0.00848453 High-group

PPP1R27 -1.516859253 0.000798041 0.008494052 Low-group

OR5V1 3.423376102 0.00080409 0.00854459 High-group

TNFRSF17 2.302775653 0.000815867 0.008656772 High-group

SMTNL2 2.073864355 0.000815964 0.008656772 High-group

TNMD -2.281431327 0.000817475 0.008663475 Low-group

CBLN2 3.852876754 0.000820077 0.008686388 High-group

CNR2 1.975428573 0.000821489 0.008692906 High-group

HS3ST6 2.073840228 0.000823059 0.008703941 High-group

NAALADL2 -1.223668088 0.000831542 0.008774072 Low-group

PPP1R3A 4.616243591 0.000831917 0.008774072 High-group

CYP2C9 -1.709293709 0.000836882 0.0088123 Low-group

TMEM136 1.056114778 0.000839274 0.008828048 High-group

ZIC2 1.954662448 0.000843594 0.008864036 High-group

INS-IGF2 -3.580273174 0.000844316 0.008866891 Low-group

ZNF737 1.449466494 0.000844882 0.008868116 High-group

MUSK 2.667026042 0.000849852 0.008913579 High-group

SQSTM1 1.10372293 0.000852475 0.008933534 High-group

SCNN1A -1.690184193 0.000853947 0.00893539 Low-group

SIM1 -2.759278817 0.000854011 0.00893539 Low-group

LONRF2 1.601083926 0.00085585 0.008949872 High-group

RP11-545J16.1 -2.266952903 0.000858497 0.0089666 Low-group

DAAM2 -1.139372655 0.000858814 0.0089666 Low-group

CES2 -1.096256573 0.000860214 0.008971924 Low-group

RNF133 1.701755578 0.000860234 0.008971924 High-group

LIPG -1.261459698 0.000864318 0.009009749 Low-group

WIF1 3.599954074 0.000867475 0.009037883 High-group

PTGDS -2.877697862 0.00087084 0.009068145 Low-group

RAP1GAP 1.117243445 0.000877887 0.009131876 High-group

SYT5 1.818443612 0.000878832 0.009136888 High-group

PLEKHN1 1.528762636 0.000883758 0.009183257 High-group

HKDC1 1.529011125 0.000884803 0.009189274 High-group

LAD1 1.564355364 0.000893031 0.009267608 High-group

PARK2 -1.076595908 0.000893286 0.009267608 Low-group

SLC5A10 -1.830511216 0.000894005 0.00927019 Low-group

ADAMTS19 3.719319079 0.000896296 0.009284178 High-group

SPTSSB 2.237423476 0.00089774 0.009294249 High-group

HRK -3.305008393 0.000901829 0.009321897 Low-group

G6PC2 -1.914009032 0.000907235 0.009365444 Low-group

HPR -1.492110761 0.000907943 0.009365444 Low-group

CPSF4L 1.708531326 0.000913588 0.009413822 High-group

SH3GL2 -2.522515723 0.000914903 0.009421881 Low-group

MIOX 2.374722161 0.000915326 0.009421881 High-group

UGT1A4 -2.167075504 0.000916441 0.009428437 Low-group

MYL1 -2.498913599 0.000917094 0.009430229 Low-group

TSPAN10 -1.692072668 0.000920167 0.009453403 Low-group

GREM2 -2.371145798 0.000920306 0.009453403 Low-group

SLC13A5 -1.437896471 0.000922886 0.009465103 Low-group

GLUL -1.557235545 0.000924101 0.009472633 Low-group

AIM1L 1.339625988 0.000925617 0.009478322 High-group

AADAT -1.447816585 0.00092949 0.009508099 Low-group

SNTG1 -2.417188919 0.000930093 0.009509331 Low-group

SYCP2 1.597002148 0.000931729 0.009521121 High-group

PROK1 -2.122700012 0.000942878 0.009610142 Low-group

CCNE2 1.337484061 0.000947132 0.00964851 High-group

HAO2 -2.016565805 0.000950152 0.009670209 Low-group

BMPER -1.901767461 0.000963489 0.009784803 Low-group

IGFL4 2.235327158 0.000964589 0.009785891 High-group

TTC39A 1.587138998 0.000966502 0.009800256 High-group

MAGEA10 3.511641989 0.000973515 0.009856155 High-group

MYO16 -1.367720823 0.000974202 0.009858049 Low-group

CTD-2583A14.9 -1.275454919 0.000982738 0.009934217 Low-group

LRTM2 3.356936015 0.000985701 0.009947283 High-group

ATOH1 3.842072426 0.000986049 0.009947283 High-group

GLYAT -2.032292643 0.000988453 0.009961341 Low-group

ZNF83 1.367152431 0.000989305 0.009964835 High-group

ANKRD24 -1.069267672 0.000993734 0.009994124 Low-group

TNNI2 1.693479461 0.001004345 0.010075136 High-group

MAB21L2 2.304059723 0.001007107 0.010097706 High-group

CMBL -1.045405604 0.001008099 0.010102513 Low-group

ZNF14 1.26891439 0.001015862 0.010171532 High-group

KRTAP19-1 3.929727948 0.001021854 0.010209221 High-group

ANXA13 1.540088805 0.001024592 0.010231388 High-group

ARHGDIG -2.248127775 0.001028409 0.010259108 Low-group

KRTAP7-1 -3.981711066 0.00103983 0.010352081 Low-group

TBR1 2.923417139 0.001043579 0.010384161 High-group

LHX6 -1.181822736 0.001047898 0.010416625 Low-group

SBSPON -1.62114205 0.001055838 0.010484972 Low-group

PLA2G2A -2.480892287 0.001058972 0.010510806 Low-group

DDR1 1.324819684 0.001062955 0.010532158 High-group

HSH2D 1.496517996 0.001063261 0.010532158 High-group

KCNU1 -3.617262933 0.001066214 0.01054842 Low-group

OR8A1 3.468194671 0.001066508 0.01054842 High-group

CYP51A1 -1.044188228 0.001068064 0.01055322 Low-group

MFSD6L 2.350728939 0.001071075 0.010572375 High-group

ZBTB7C -1.431807497 0.001072158 0.01057777 Low-group

KRT77 3.230769627 0.001074644 0.010591691 High-group

MYB 1.361690586 0.001085036 0.0106781 High-group

HSD11B1 -1.95131919 0.001090201 0.010710424 Low-group

DSG1 -1.695726421 0.001093249 0.01073213 Low-group

FAM43B -1.791395966 0.00110328 0.010809074 Low-group

PLBD1 1.307666793 0.001104532 0.010815962 High-group

HRASLS5 -2.62042981 0.001107171 0.010825671 Low-group

ANK1 1.79334688 0.001122064 0.010927932 High-group

ZNF506 1.044330711 0.001124967 0.010945387 High-group

PRLH -4.592388416 0.001126395 0.010952351 Low-group

STAB2 -1.669619125 0.001126794 0.010952351 Low-group

SEMA3C 1.737403425 0.001132194 0.010993992 High-group

TRPA1 2.31262361 0.001132816 0.010994616 High-group

ARHGAP20 -1.293722076 0.00113792 0.011038723 Low-group

DMKN 2.244766988 0.001148846 0.011130997 High-group

CACNA1B 2.872173272 0.001149126 0.011130997 High-group

CYP46A1 -1.205727026 0.001155858 0.011179719 Low-group

TRIM45 1.080695243 0.001158341 0.01119547 High-group

ZNF880 1.479695813 0.001158971 0.01119547 High-group

MT1M 2.723982761 0.001161389 0.011211217 High-group

DBN1 1.123164956 0.001170642 0.011295003 High-group

CLEC4M -3.179943553 0.001172621 0.011303036 Low-group

FOLH1 -1.173583785 0.001175668 0.011326862 Low-group

PRKAR2B -1.815694515 0.001177043 0.0113344 Low-group

PRKCDBP -1.185580064 0.0011776 0.0113344 Low-group

SLCO2B1 -1.033838303 0.001178516 0.011337683 Low-group

EBF1 -1.340216503 0.001180871 0.011348889 Low-group

SP140 1.312253312 0.001181348 0.011348889 High-group

NR1I2 -1.597998959 0.001181408 0.011348889 Low-group

TLR3 -1.044745334 0.001188484 0.011400188 Low-group

MERTK -1.05806926 0.001204987 0.011541641 Low-group

CHRNA3 -2.80115725 0.001209228 0.011575203 Low-group

ZNF735 -3.702184383 0.001212041 0.01158992 Low-group

TNFRSF13B 1.989520066 0.001212379 0.01158992 High-group

INS -3.044576611 0.001220918 0.011646509 Low-group

HUNK 1.655835882 0.001221438 0.011646509 High-group

DUOXA2 3.277553022 0.001221844 0.011646509 High-group

KIF5C -1.766581415 0.001223104 0.01165289 Low-group

KIAA1244 1.628532279 0.001224787 0.011658146 High-group

GMNC -1.592831388 0.001225157 0.011658146 Low-group

CACNA1E 1.813934683 0.001235108 0.011725094 High-group

CCDC177 -2.189039307 0.001248368 0.011836376 Low-group

TMC5 2.317632787 0.001252642 0.011865491 High-group

GSTM1 -2.657592235 0.001264175 0.011950674 Low-group

MTFR2 1.121977565 0.001273453 0.012022177 High-group

VAV3 1.181475125 0.001275502 0.012029995 High-group

ADAM28 1.405914317 0.001278954 0.012049999 High-group

SLCO1B1 -1.443849161 0.001282146 0.012058046 Low-group

C5orf30 1.388568918 0.001285032 0.012079422 High-group

SYT15 1.604965752 0.001294815 0.012138846 High-group

AUNIP 1.077994985 0.001296953 0.01215092 High-group

INPP5J 1.275514685 0.001312201 0.012264942 High-group

POU2F3 -1.739919904 0.001315925 0.012287781 Low-group

TMEM125 2.185568283 0.00132989 0.012406436 High-group

TPBGL -1.856539594 0.001331707 0.012411929 Low-group

MS4A10 -3.214066268 0.001331738 0.012411929 Low-group

CNTN4 -1.328829696 0.001336627 0.012439845 Low-group

BRS3 3.44493442 0.001340399 0.012457304 High-group

GCSAML -2.270179524 0.001345436 0.012498228 Low-group

EPHA7 3.511616138 0.001346716 0.012504222 High-group

GJB3 1.741178368 0.001348777 0.012513322 High-group

PDX1 2.681142915 0.001349988 0.012516914 High-group

DRP2 1.616406206 0.001350751 0.012518103 High-group

RAPH1 -1.085473088 0.001354993 0.012541068 Low-group

RDH16 -1.564133541 0.001355774 0.012541068 Low-group

ARHGEF26 -1.087942857 0.001358834 0.012563479 Low-group

A1BG -1.401210185 0.001364405 0.012592933 Low-group

PHGR1 -2.587202243 0.001365089 0.012592933 Low-group

PPARGC1A -1.318039558 0.001368171 0.012614309 Low-group

PON1 -1.331759005 0.001369363 0.012619392 Low-group

OAT -1.396866874 0.001382893 0.012719835 Low-group

MDFI 1.510269575 0.001388416 0.012759185 High-group

FGF2 -1.517947655 0.001391145 0.012766983 Low-group

ANGPTL2 -1.133497913 0.001391157 0.012766983 Low-group

ENTPD3 1.855323335 0.001398921 0.012813911 High-group

ATP6V0A4 1.725099594 0.001409322 0.012903181 High-group

FA2H 2.219723855 0.001411786 0.012913157 High-group

GPRC5D 1.191517314 0.001412377 0.012913157 High-group

C10orf10 -1.082748754 0.00141316 0.012914329 Low-group

FAM72D 1.226627244 0.00142269 0.012995395 High-group

FAM170A -1.628558989 0.001429934 0.013055513 Low-group

PON3 -1.045942963 0.001430804 0.013057416 Low-group

CSF3 -2.045627193 0.001436023 0.013092915 Low-group

INSL3 1.390571911 0.001439609 0.013119546 High-group

BCO2 -1.660045425 0.00144421 0.013145045 Low-group

POLN -1.256273355 0.001444581 0.013145045 Low-group

DACH2 2.514621377 0.00144635 0.013150574 High-group

PROL1 -2.377980746 0.001454438 0.013211927 Low-group

HTR2A -1.203601915 0.001456837 0.013226032 Low-group

P2RY10 1.853088406 0.001457333 0.013226032 High-group

SPARCL1 -1.358917103 0.001465071 0.013290146 Low-group

C1orf111 -2.022892502 0.001471201 0.013327346 Low-group

AC010642.1 1.063527514 0.001473804 0.013336748 High-group

SMPDL3B 1.547802771 0.001474268 0.013336748 High-group

10-Mar 2.038051112 0.00147844 0.013368353 High-group

TGFB1 1.012757295 0.001485817 0.013422741 High-group

NR4A1 -1.084535413 0.001505675 0.013571044 Low-group

CD36 -1.30594487 0.001509617 0.013600351 Low-group

CD79B 1.184421946 0.001512954 0.013623184 High-group

UBASH3B 1.094687933 0.001514808 0.013628436 High-group

KRTAP20-2 3.529749813 0.00151649 0.013637338 High-group

SLC44A3 1.108215122 0.001518467 0.013645994 High-group

C5orf38 2.583041814 0.001524649 0.013679507 High-group

KCNJ9 2.655264157 0.001545608 0.013820726 High-group

FAM132A 1.393452199 0.001547242 0.013820726 High-group

IL22RA2 2.513074487 0.001547438 0.013820726 High-group

PCNXL2 1.219404347 0.001547754 0.013820726 High-group

RHPN1 1.053198212 0.001548101 0.013820726 High-group

SFRP5 3.074139821 0.001550565 0.013830199 High-group

FTHL17 4.78551869 0.001553672 0.013851644 High-group

CDH26 1.692177761 0.001555071 0.01385785 High-group

ZNF354C 1.223054668 0.001560379 0.013890475 High-group

HEPACAM2 2.376182151 0.001561551 0.013890475 High-group

EPB42 -2.051374046 0.001567465 0.013936797 Low-group

GINS4 1.006270471 0.001570171 0.013954555 High-group

GOLGA8O -1.818160236 0.001575553 0.013996083 Low-group

MS4A8 2.916222547 0.001579592 0.01402373 High-group

PSMB11 -1.977048454 0.001584019 0.01404597 Low-group

CIB2 1.348917837 0.00158676 0.014063943 High-group

TEX33 -2.381086972 0.001588475 0.014066492 Low-group

UMODL1 2.482584118 0.001593821 0.014101163 High-group

TNFRSF11A 1.150702537 0.001600882 0.01415433 High-group

MAGED4 2.595648496 0.001601266 0.01415433 High-group

IL21R 1.400132425 0.001602695 0.014160609 High-group

CHADL -1.250326306 0.001604356 0.014168934 Low-group

DNAH8 1.674835188 0.001607399 0.014176753 High-group

XKR6 1.453098563 0.001620177 0.014263929 High-group

PAIP2B -1.433644424 0.001624693 0.014297294 Low-group

G6PC -1.406993293 0.001638625 0.014391941 Low-group

C15orf48 1.725557517 0.00163927 0.014391941 High-group

CHST1 1.033819705 0.001639968 0.014391941 High-group

NMUR1 -1.156605677 0.001640559 0.014391941 Low-group

CAPN9 1.954007326 0.001643784 0.014407408 High-group

FBLN5 -1.110555999 0.001650115 0.014456478 Low-group

CH507-9B2.3 -1.088937417 0.001653247 0.014471053 Low-group

TKT 1.048303436 0.001665363 0.014564171 High-group

MYO3A 3.543473959 0.001675782 0.014635803 High-group

ZNF812 1.929785602 0.001678914 0.014656668 High-group

TRPC1 1.140159444 0.001682998 0.014682481 High-group

BEST1 1.177197897 0.001695851 0.014758773 High-group

GLUD2 1.22187117 0.001701029 0.014790773 High-group

SFTPD -1.720021421 0.001703002 0.014795035 Low-group

HMCN2 -1.66595692 0.001703681 0.014795035 Low-group

NELL1 3.049422318 0.001704812 0.014797561 High-group

C14orf180 -2.964886558 0.00170989 0.0148351 Low-group

ARNT2 1.632892672 0.001718027 0.014899145 High-group

AGXT2 -1.523748442 0.00172287 0.01492801 Low-group

CES3 -1.248415229 0.001728261 0.014962156 Low-group

TANC2 1.272422491 0.001728329 0.014962156 High-group

CEACAM5 3.204931592 0.001735162 0.01500154 High-group

ALOX5AP 1.279110339 0.001737101 0.015005146 High-group

DSG2 1.207087674 0.00174315 0.015044532 High-group

SLC45A4 1.288679189 0.001745271 0.015050013 High-group

PSD3 -1.11205374 0.00174535 0.015050013 Low-group

MTNR1B 4.021727062 0.001746622 0.015054397 High-group

VWA5B1 -2.786894365 0.001773371 0.015264932 Low-group

SLX1A 1.433671441 0.001777397 0.015269413 High-group

GSTA3 -1.627610997 0.001779236 0.015269413 Low-group

CDR1 2.963930988 0.001779314 0.015269413 High-group

RAB38 1.466818982 0.001798171 0.01541111 High-group

FAM198A -1.707580422 0.001799379 0.015414758 Low-group

CD248 -1.165563304 0.001802673 0.015436264 Low-group

PTCHD3 2.912264837 0.001806574 0.015462959 High-group

WDR63 -1.930233965 0.001810274 0.015481181 Low-group

CTB-133G6.1 1.558334202 0.00181203 0.015489474 High-group

NKAPL -1.953877185 0.001813288 0.015489549 Low-group

ASCL1 -2.75989283 0.00181361 0.015489549 Low-group

PAX3 3.646312835 0.00181969 0.015528026 High-group

SOX14 3.961719733 0.001821167 0.0155339 High-group

CHRM2 -3.05393053 0.001829573 0.015592105 Low-group

TCL1B 2.665979598 0.001834354 0.015614569 High-group

SLCO1B7 -2.193522538 0.001840738 0.015653419 Low-group

CLDN6 2.028229316 0.001845574 0.015687778 High-group

TGFBR3 -1.033791745 0.001847491 0.015697307 Low-group

GAGE12J 3.095122947 0.001851247 0.015722435 High-group

RHOXF2 4.327697819 0.001865511 0.015829939 High-group

PYDC1 3.175884746 0.001869547 0.015853671 High-group

KCNJ12 -1.272984102 0.001871229 0.015857985 Low-group

ADAMDEC1 1.81519669 0.00187236 0.015860747 High-group

SPTBN2 -1.197246988 0.001888044 0.01598277 Low-group

COL4A4 -1.321766782 0.001888386 0.01598277 Low-group

SLC35F3 2.251492384 0.001897702 0.016040951 High-group

KRTAP5-1 1.605771693 0.001899192 0.016046664 High-group

RNF182 1.984411931 0.001902802 0.016064772 High-group

PRR19 -1.564440794 0.001905438 0.016071884 Low-group

ACSM3 -1.314286557 0.001908033 0.016080007 Low-group

CYP2A7 -2.662978369 0.001911976 0.016106359 Low-group

FAM153C 1.898995285 0.001918564 0.016148047 High-group

F8A3 -1.624841746 0.001920299 0.016155754 Low-group

C2orf83 2.706949115 0.001926915 0.016197594 High-group

SOX21 2.375554628 0.001941234 0.016290165 High-group

WNT9B -1.932579449 0.001945584 0.016319725 Low-group

MSI1 1.661518815 0.001954874 0.016390674 High-group

CMTM5 -1.995541434 0.001961035 0.016435342 Low-group

ADRA1D -2.188005133 0.001964616 0.016458356 Low-group

CCDC154 1.337606062 0.001968317 0.016482354 High-group

VIPR1 -1.448785362 0.001971031 0.016498073 Low-group

C6orf15 4.630051672 0.001974188 0.016508794 High-group

ZNF503 -1.095245069 0.001976499 0.016508794 Low-group

IER3 1.037184123 0.001979653 0.016528134 High-group

FLNC 1.955706102 0.001982097 0.016541529 High-group

C19orf80 1.583580358 0.001984067 0.016550967 High-group

PRDM7 2.193029773 0.001985523 0.016556102 High-group

CARD11 1.247416843 0.001998435 0.016656725 High-group

ZNF528 1.18724771 0.002002793 0.016678017 High-group

SRL -1.03724745 0.002003528 0.016678017 Low-group

HHIPL2 2.225948722 0.002017036 0.016769213 High-group

GNGT1 3.160116033 0.002024285 0.016808217 High-group

MOGAT2 -1.749708285 0.002044122 0.01695692 Low-group

IGFALS -1.594625793 0.002045635 0.01695692 Low-group

PRKX 1.0511944 0.002065093 0.017089472 High-group

ST14 1.311108952 0.002073617 0.017152805 High-group

SBK3 1.945501948 0.002076681 0.017170945 High-group

GOLGA6D -2.0159536 0.002080722 0.017189935 Low-group

ADAMTS3 1.369817102 0.002089028 0.017230144 High-group

NCAM2 -1.894585617 0.002089085 0.017230144 Low-group

ZNF331 1.015604098 0.002092477 0.017250902 High-group

FGA -1.0681029 0.002107507 0.017345797 Low-group

KCNB1 -1.823861587 0.002115342 0.017403017 Low-group

SYT7 -1.609365805 0.002133292 0.017521436 Low-group

MME -2.159481855 0.002154103 0.017657713 Low-group

TESC 1.491421186 0.002154982 0.017657713 High-group

PDGFRL 1.152613593 0.002163469 0.017710256 High-group

NDUFA4L2 1.040344488 0.002188652 0.017886698 High-group

SLC6A20 -2.699178917 0.002189578 0.017886847 Low-group

DYNLRB2 -1.448790872 0.002193661 0.017912781 Low-group

STK31 -1.911191572 0.002218723 0.018094942 Low-group

FAXC 2.071030141 0.002226042 0.018147118 High-group

ZNF682 1.158667984 0.002230115 0.018165303 High-group

XKR7 2.930464819 0.002248261 0.018275347 High-group

PCDH11X 3.165840886 0.002249483 0.018277749 High-group

SULT1C4 1.466463469 0.002263308 0.018367361 High-group

GALNT8 -1.72785251 0.002266613 0.018386612 Low-group

MRVI1 -1.167690344 0.002279121 0.018465061 Low-group

AHNAK2 2.035829704 0.002281353 0.018468185 High-group

TTC22 1.056537686 0.002287285 0.018501012 High-group

H2AFY2 1.150830838 0.00229054 0.018512148 High-group

KHDC1 1.35654428 0.002296211 0.018528669 High-group

INHBC -1.253443908 0.002296344 0.018528669 Low-group

SLC11A1 1.164113205 0.002297823 0.018533016 High-group

C1QL2 -2.208907286 0.002298905 0.018534161 Low-group

TNNT2 2.096987963 0.002308596 0.018604678 High-group

ULK4 -1.040707968 0.002316746 0.018639877 Low-group

SPAG17 1.950629616 0.002317872 0.018641323 High-group

LTF -1.795837535 0.002319803 0.018649249 Low-group

NKX2-8 3.120195222 0.002331449 0.0187276 High-group

CAMK2B -1.763670696 0.002333697 0.018736506 Low-group

TJP3 1.065832165 0.002353288 0.018864582 High-group

CH25H -1.642978657 0.002354776 0.018866543 Low-group

CD52 1.106070972 0.002356306 0.018866543 High-group

C9orf84 1.601456158 0.002356404 0.018866543 High-group

LMLN -1.043691206 0.002368618 0.018952166 Low-group

HIST1H1T 1.895169275 0.002369021 0.018952166 High-group

TEPP -1.997587431 0.002370596 0.018956171 Low-group

KBTBD11 -1.331366662 0.002371445 0.018956171 Low-group

RBMXL2 -1.717759591 0.002389135 0.019061949 Low-group

NDNF -2.012665175 0.002391363 0.019067467 Low-group

SLC47A1 -1.022787268 0.002392139 0.019067467 Low-group

UGT1A3 -1.906746199 0.002425571 0.019256084 Low-group

SLC18A3 -3.641895607 0.002433011 0.019307373 Low-group

TUBA3C 3.474597769 0.002434299 0.019309817 High-group

ZNF300 1.242617815 0.00243552 0.019311736 High-group

CENPA 1.129108838 0.002448275 0.019389476 High-group

TC2N 1.524751438 0.002450081 0.019395987 High-group

RAB34 1.240322871 0.00245875 0.019448992 High-group

NPIPB5 1.253909166 0.002464179 0.019484111 High-group

CHIT1 2.105112988 0.002475131 0.01956286 High-group

DAW1 2.086507479 0.002477809 0.019576182 High-group

VTCN1 2.318944629 0.002484303 0.019611944 High-group

RAMP1 -1.188772284 0.002484326 0.019611944 Low-group

CA10 -2.321511638 0.002493689 0.019670106 Low-group

FKSG62 -1.907585004 0.00249781 0.01969473 Low-group

UGT2B7 -1.510109182 0.002499539 0.019694962 Low-group

KNG1 -1.09024669 0.002499837 0.019694962 Low-group

RP11-574F21.3 -1.128534283 0.00251924 0.019816148 Low-group

LIF 1.320296457 0.002521465 0.019825733 High-group

REG3G -3.918482811 0.002531781 0.019883517 Low-group

C1orf100 1.611490979 0.00253184 0.019883517 High-group

DCD 2.550405879 0.002536238 0.019910129 High-group

RIPPLY2 3.14361492 0.002555383 0.020036481 High-group

POMC -1.024053193 0.002559184 0.020058302 Low-group

CHST11 1.086030008 0.002581952 0.020196604 High-group

KCNE4 -1.32972253 0.002585976 0.020220053 Low-group

SERPINB3 3.886211263 0.002589967 0.020243228 High-group

SYNPO2 -1.084741346 0.002596589 0.020276347 Low-group

GTF2A1L -3.146688629 0.002598318 0.020276347 Low-group

SVOP -1.320004054 0.002601119 0.020287986 Low-group

PLA2G2D 1.918300274 0.002629409 0.020478429 High-group

NR4A2 -1.068399998 0.002641191 0.020545837 Low-group

WNK2 1.922650378 0.002644231 0.020561375 High-group

SH2D4B -1.394426637 0.002650622 0.020602944 Low-group

GDF10 2.518722375 0.002660883 0.020658262 High-group

TMEM232 -1.194190481 0.002668296 0.020707658 Low-group

SPINK4 2.385265777 0.002674551 0.020748029 High-group

LRP12 1.146200077 0.002676053 0.020751517 High-group

CSGALNACT1 1.04232679 0.002682299 0.020791774 High-group

ZNF135 1.340933945 0.002685858 0.02081118 High-group

GLS2 -1.949519016 0.002693881 0.020834397 Low-group

NEUROD4 2.910079098 0.002694606 0.020834397 High-group

TEX101 -2.964357705 0.002694996 0.020834397 Low-group

HYDIN -2.1029657 0.002714994 0.02095234 Low-group

AC104532.2 -1.402408174 0.002719943 0.02097634 Low-group

PDZD4 -1.185135938 0.002728095 0.021015677 Low-group

SLCO4A1 1.582210544 0.002728243 0.021015677 High-group

CCDC27 1.904919046 0.002737612 0.02107961 High-group

UBE2QL1 -1.427226206 0.002751814 0.021172427 Low-group

CLCN1 -1.88524126 0.002755941 0.021187671 Low-group

CCL20 1.554860098 0.002764987 0.021217636 High-group

DLG2 -1.049124295 0.002765225 0.021217636 Low-group

CAPG 1.033398204 0.00278615 0.021358708 High-group

LRRC75A 1.010631256 0.002786861 0.021358708 High-group

GAST 2.980435421 0.002806428 0.021470177 High-group

POU3F3 2.669191573 0.002810758 0.021477548 High-group

CCDC64 1.023721931 0.002811918 0.021477548 High-group

C11orf88 -2.023947182 0.002812173 0.021477548 Low-group

DEFB126 3.16809069 0.002821194 0.021521432 High-group

SYT10 -3.042410088 0.002850814 0.021688635 Low-group

CXCL1 1.982050097 0.002868368 0.02178015 High-group

ANO1 -1.413098447 0.002889747 0.021925591 Low-group

DIO2 -2.0916419 0.002894282 0.021939908 Low-group

PKD1L3 -1.338139295 0.002894973 0.021939908 Low-group

PHLDA2 1.267773259 0.002907797 0.022004754 High-group

CDH3 1.952902774 0.002929394 0.022127753 High-group

FRMD5 1.685652175 0.002929797 0.022127753 High-group

ANKLE1 1.096120194 0.002929862 0.022127753 High-group

GPR114 -1.57671545 0.002951814 0.022274078 Low-group

MDGA1 -1.379514755 0.002953756 0.022274078 Low-group

SMIM23 2.494500661 0.002955183 0.022276316 High-group

FSTL1 -1.028572561 0.002966394 0.022343739 Low-group

AFAP1L2 1.124104052 0.002974181 0.022393831 High-group

SI -4.039508984 0.002985559 0.022462342 Low-group

QRFPR -1.79725669 0.002992696 0.022507451 Low-group

PAPPA2 -1.934975198 0.003002018 0.022560345 Low-group

RASGEF1A 1.644178326 0.003006488 0.022583846 High-group

SLC9A3 1.863461311 0.003017237 0.022640191 High-group

AIM1 -1.023238726 0.00304345 0.022782076 Low-group

C1QTNF9 -1.39101586 0.003045009 0.022782076 Low-group

CLCA2 -3.041174458 0.003054112 0.022821329 Low-group

GFI1B -1.990645622 0.003057841 0.022840528 Low-group

ZNF701 1.091528624 0.003071666 0.022935103 High-group

SHISA6 -1.586281573 0.003095282 0.023074074 Low-group

LYPD1 1.631987852 0.003095779 0.023074074 High-group

MYEOV 2.08221378 0.003118666 0.023189416 High-group

C10orf35 1.061903272 0.003140082 0.023339857 High-group

GPR37L1 1.363216644 0.003168798 0.02350013 High-group

RSPH1 1.353421311 0.003183615 0.023565687 High-group

RASSF10 2.58851203 0.00321422 0.023738746 High-group

NRG3 1.645990995 0.003217579 0.023754649 High-group

C9orf152 -1.755159506 0.003222096 0.023770192 Low-group

ST8SIA3 -2.709651301 0.003227863 0.023794924 Low-group

JAKMIP2 -1.205936122 0.003230867 0.023808168 Low-group

IL4 2.03957134 0.003233646 0.023812357 High-group

SLC22A15 1.376025551 0.003237397 0.023829567 High-group

RUFY4 1.301677879 0.003239627 0.023837073 High-group

DCLK2 -1.206467894 0.00324535 0.023869083 Low-group

CABYR 1.791244269 0.003246399 0.023869083 High-group

FCAMR -2.01741743 0.003248072 0.023872479 Low-group

PRR23D1 3.917565886 0.003254364 0.023890064 High-group

KLHL30 1.758486116 0.003263912 0.023926453 High-group

ASB4 -1.639841582 0.003297881 0.024121641 Low-group

MCIDAS 1.966468709 0.003299234 0.024122591 High-group

OR8G5 3.0578566 0.00330712 0.02416232 High-group

BARX2 2.087364347 0.003308557 0.024163865 High-group

KLK1 1.662618487 0.003333174 0.02430125 High-group

TMC6 1.010955777 0.003333532 0.02430125 High-group

PDILT -1.555532946 0.003349498 0.0243979 Low-group

DLGAP2 -2.068142484 0.003350503 0.0243979 Low-group

AC138969.4 1.223450947 0.003373563 0.024529572 High-group

ZNF439 1.07932084 0.003384457 0.024599712 High-group

CYP4A22 -1.323704477 0.003386501 0.024603587 Low-group

TSPAN19 3.12266373 0.003387487 0.024603587 High-group

TEK -1.031441477 0.003400323 0.024687719 Low-group

SOX9 1.28000936 0.003421208 0.024802818 High-group

TEKT1 -1.278295918 0.003433771 0.024866457 Low-group

RIMS3 1.031765548 0.003447471 0.024937662 High-group

C6orf25 -1.39934216 0.003448664 0.024937662 Low-group

OTX2 3.388273241 0.003456454 0.024957943 High-group

DIRC3 -1.646333581 0.003462229 0.024989915 Low-group

ZNF488 1.540656237 0.00347602 0.025069631 High-group

TCP10L -1.429155645 0.003499686 0.025214111 Low-group

KIF5A 1.504301303 0.003513333 0.025287551 High-group

SDCBP2 1.242877435 0.003513728 0.025287551 High-group

PRR5L -1.186823044 0.003518162 0.025307174 Low-group

CHST10 1.07161691 0.003522122 0.025307174 High-group

PNOC 1.971421457 0.003525613 0.025317629 High-group

SELL 1.308526651 0.003538657 0.025383557 High-group

PRAMEF25 2.487378827 0.00354417 0.025413851 High-group

TMEM132D 1.705740513 0.003567122 0.025550548 High-group

DPYS -1.283418467 0.003570555 0.025561685 Low-group

LETM2 1.295954756 0.003579741 0.025594437 High-group

DEFB132 -2.07303411 0.00360261 0.025739268 Low-group

PPP1R14A -1.155143626 0.003604836 0.02574584 Low-group

HTR5A -3.543966971 0.003624911 0.02587047 Low-group

CA5B -1.062294944 0.003642293 0.025972467 Low-group

PGLYRP4 1.802895736 0.003644473 0.025972467 High-group

IGFBP6 -1.51929977 0.00365671 0.02604085 Low-group

DAB1 -1.299920571 0.003659907 0.026054203 Low-group

GPRIN1 1.069700117 0.003664843 0.026070508 High-group

IQCJ-SCHIP1 -1.013436403 0.003677495 0.026132386 Low-group

ZNF878 1.228848089 0.003688234 0.026180216 High-group

FAM81B 1.725388071 0.003690943 0.02618415 High-group

ANKRD18A 1.732967853 0.003691559 0.02618415 High-group

DLG3 1.047494496 0.003697676 0.026209492 High-group

C1orf106 1.601687882 0.003700626 0.026217014 High-group

SLC22A25 -1.22470169 0.003701398 0.026217014 Low-group

SALL2 1.271386363 0.003736656 0.026400347 High-group

TSPY2 -4.788793841 0.003752223 0.026491339 Low-group

MAGED4B 2.028198874 0.003769587 0.026594882 High-group

RHOF 1.041066009 0.003777835 0.026629601 High-group

RALGPS1 -1.083555267 0.003778561 0.026629601 Low-group

FGF10 2.621240446 0.003816738 0.026850653 High-group

STRC 1.90100408 0.003832333 0.026941136 High-group

SST 4.254393745 0.003835899 0.026950959 High-group

KRTAP5-7 2.26506782 0.003836465 0.026950959 High-group

SLC10A1 -1.732747802 0.003838387 0.026954859 Low-group

ANKRD34C -2.243939637 0.003844075 0.026965981 Low-group

SAA2-SAA4 -2.350548657 0.003849107 0.026991676 Low-group

EXTL1 -1.380977873 0.003850511 0.026991915 Low-group

IL27 -1.088961602 0.003863527 0.02705221 Low-group

PENK -3.195412139 0.003880031 0.027131315 Low-group

TERT -2.148473882 0.003881896 0.027134727 Low-group

ENHO 1.412232649 0.003885869 0.027152111 High-group

BTN1A1 1.829006561 0.003887138 0.027152111 High-group

CD5L -1.697060559 0.003922992 0.027363774 Low-group

RXRG -2.112809061 0.003927722 0.027377386 Low-group

LGI2 1.178288984 0.003941946 0.02744742 High-group

FSD1 -1.375678088 0.00394639 0.027468657 Low-group

CATSPER4 2.215266497 0.003957331 0.027505965 High-group

OR3A3 -3.376254436 0.003966597 0.027560043 Low-group

BCAS1 1.63762563 0.003967908 0.027560043 High-group

VNN2 1.463928212 0.003992248 0.027719335 High-group

PNPLA1 1.467288522 0.003999473 0.02774995 High-group

PPM1K -1.023561943 0.004012829 0.027832645 Low-group

AMDHD1 -1.015651837 0.004017938 0.027838871 Low-group

KRTAP5-5 2.864539161 0.004031324 0.027877011 High-group

ZNF233 1.180791812 0.004031928 0.027877011 High-group

TMED6 -1.323123101 0.004047132 0.027952722 Low-group

ZNF468 1.01765703 0.004056052 0.02799471 High-group

KRT85 2.320122968 0.004070028 0.028071515 High-group

IAPP -1.496618889 0.004102032 0.028262585 Low-group

HFE2 -1.038133202 0.00410569 0.028277903 Low-group

NAV3 1.71183535 0.004109429 0.028283759 High-group

GPR82 1.094908921 0.004117258 0.028318001 High-group

S100B 1.257414966 0.004124761 0.02835971 High-group

CCSER1 1.483785141 0.00412757 0.028369126 High-group

CHRM1 -3.621242201 0.004130753 0.028369655 Low-group

TNFRSF10C 1.047257657 0.004131144 0.028369655 High-group

REG1A -3.898568789 0.004131964 0.028369655 Low-group

DCDC2 1.584356938 0.004166877 0.028536509 High-group

ABCB5 -2.668400835 0.004185017 0.028644113 Low-group

BASP1 -1.433180056 0.004198616 0.02869734 Low-group

PTGER4 1.02788775 0.004232602 0.028875183 High-group

CTD-2207O23.3 2.552459868 0.004233425 0.028875183 High-group

TDO2 -1.414537096 0.004246483 0.028944215 Low-group

EPS8L1 1.537910752 0.004260163 0.029000458 High-group

FAIM3 1.24624919 0.004260619 0.029000458 High-group

ACMSD -1.032743575 0.004278754 0.029113839 Low-group

ZNF28 1.152652813 0.004290978 0.029172436 High-group

COL9A3 1.484513235 0.004291805 0.029172436 High-group

AVPR2 -1.256144869 0.004299904 0.029209791 Low-group

KRT24 -2.588506588 0.004301316 0.029209791 Low-group

IL20RA 2.558233722 0.004301746 0.029209791 High-group

TMEM31 -1.943013417 0.004312329 0.029271568 Low-group

DCAF4L1 -1.515432173 0.004328971 0.02935857 Low-group

MTRNR2L5 -1.910853502 0.004337604 0.029382421 Low-group

VGLL1 -2.509990729 0.004341904 0.029391352 Low-group

PDE9A 1.143101265 0.004388981 0.029648934 High-group

PANX3 2.750034892 0.004396676 0.029680576 High-group

BOC -1.072594912 0.004422818 0.029819023 Low-group

COCH 1.681154758 0.00444604 0.029952266 High-group

RBPJL 2.216086638 0.004453101 0.029989588 High-group

ZNF541 -1.326193279 0.004463379 0.030048538 Low-group

MIXL1 1.50678896 0.004485791 0.030127399 High-group

B3GALT2 1.54022339 0.004500856 0.030169586 High-group

MTRNR2L3 -1.328361473 0.004512855 0.030226775 Low-group

ADORA1 1.501854266 0.004518736 0.030253652 High-group

CPEB1 -2.409008553 0.004519937 0.030253652 Low-group

HMSD 1.681199498 0.004537536 0.030340538 High-group

GATA1 -1.634487104 0.004567232 0.030487396 Low-group

QSOX1 1.260700498 0.004575723 0.030523403 High-group

ZNF681 1.159337592 0.004594965 0.030612296 High-group

RASGEF1C 1.725548754 0.004596192 0.030612296 High-group

SLC35F4 -1.623600879 0.004600884 0.030618711 Low-group

SLC6A5 2.900681589 0.004603165 0.030623555 High-group

CNTFR -1.761672581 0.004605152 0.030626437 Low-group

ADRB2 -1.121212911 0.004610872 0.030640976 Low-group

VSX1 1.412527567 0.004627879 0.030725747 High-group

RHOH 1.167681378 0.00463124 0.03073771 High-group

TTPA -1.152239831 0.00463366 0.030743424 Low-group

GABRB1 2.778539207 0.00464033 0.030777315 High-group

CDHR4 1.571845883 0.004646912 0.030810605 High-group

GPR88 -2.045022285 0.004657548 0.030869178 Low-group

RAX2 3.475087699 0.004671524 0.030921786 High-group

MT1X -1.663671979 0.004689441 0.031019553 Low-group

C1orf195 1.53909656 0.004692744 0.031030989 High-group

ACRV1 1.17975446 0.004695084 0.031036046 High-group

PRSS16 2.29991107 0.004700337 0.031039542 High-group

MAP3K5 -1.020504045 0.004711475 0.031092262 Low-group

SPATA9 -1.135793204 0.004722914 0.031126074 Low-group

PLTP -1.19554428 0.004734604 0.031192682 Low-group

GDF2 -3.062911374 0.004747313 0.031224239 Low-group

PRSS22 2.190874005 0.004764588 0.031316959 High-group

FBXO43 1.027444469 0.004779739 0.031395606 High-group

RHCG -2.097518528 0.004781942 0.031399613 Low-group

C12orf56 2.045424086 0.004800995 0.031482773 High-group

GRM1 1.855978183 0.004823557 0.031611695 High-group

GRAMD1B 1.377380865 0.004826094 0.031611695 High-group

VAX1 3.213720384 0.00483329 0.031641917 High-group

MYO1A 1.317789931 0.004847417 0.031713339 High-group

NQO1 1.868869117 0.004860675 0.031768442 High-group

OTOG -3.330224883 0.004864765 0.031784638 Low-group

PAGE1 3.991697128 0.004890077 0.031918275 High-group

ZNF43 1.085197542 0.004892689 0.031924758 High-group

AIM2 1.266824691 0.004897544 0.031945859 High-group

SVEP1 -1.247200003 0.004945737 0.032196291 Low-group

UCMA -2.42328294 0.004958583 0.032259091 Low-group

NAALADL1 1.258683592 0.004966652 0.032299343 High-group

HOGA1 -1.155151506 0.0049685 0.032299343 Low-group

CUX2 -1.398711225 0.004972215 0.032304649 Low-group

DYX1C1 1.321681263 0.004981865 0.032352094 High-group

C17orf61-PLSCR3 -1.234794258 0.005000542 0.032424569 Low-group

RUNDC3A 1.255554364 0.00502233 0.032555139 High-group

LRRC36 1.554954256 0.005028692 0.032585657 High-group

PTX4 -1.480841949 0.005030592 0.032587261 Low-group

CHN1 -1.108100745 0.005062592 0.032762253 Low-group

NXPH4 1.450206483 0.005077954 0.032850877 High-group

CNTNAP3B -1.518437853 0.005084132 0.032880056 Low-group

SLC15A2 1.117873695 0.005121641 0.03307983 High-group

GCSAM 1.320205555 0.005121735 0.03307983 High-group

CPA5 1.88556752 0.0051333 0.033111129 High-group

PGM5 -1.11971548 0.005136932 0.033123719 Low-group

LYPD6 1.493406294 0.005142228 0.03313619 High-group

CYP4A11 -1.16490609 0.00517918 0.033345648 Low-group

PTGER1 -1.393396411 0.005179947 0.033345648 Low-group

PCDH8 2.95049127 0.005201468 0.0334305 High-group

EPHX4 1.137519286 0.005215564 0.033499251 High-group

PCDH15 -2.226195387 0.005231478 0.03354681 Low-group

LAMA1 1.748245252 0.005241223 0.033589767 High-group

CKB -1.575321622 0.00524329 0.033589774 Low-group

LRRC74A 1.999850133 0.005247418 0.033605294 High-group

KRT23 2.171762801 0.005251903 0.033623095 High-group

CEL -1.608636451 0.005256311 0.033640391 Low-group

SUMO4 -1.361239518 0.005275879 0.033729665 Low-group

RIPPLY3 1.458914898 0.005277105 0.033729665 High-group

SLC25A47 -1.766768825 0.005317114 0.033952365 Low-group

PLA2G4F 1.946303412 0.005344169 0.03409199 High-group

PCDHGA3 -1.606969512 0.00535073 0.034122806 Low-group

TNFRSF9 -1.831105562 0.005423228 0.034495858 Low-group

BPIFB2 2.456924065 0.005428821 0.0345203 High-group

SPACA3 -3.151646426 0.005433549 0.034539217 Low-group

FGF14 -1.64673135 0.00543669 0.034548042 Low-group

MTTP -1.020336519 0.005451199 0.034617924 Low-group

NCKAP5 -1.078239571 0.00546472 0.034686252 Low-group

ZNF610 1.352743615 0.005465478 0.034686252 High-group

ETNK2 -1.193532798 0.005502528 0.034876475 Low-group

ETV4 1.497346496 0.005519889 0.034919142 High-group

PYROXD2 -1.194815046 0.005532259 0.03495874 Low-group

ITLN2 -1.60862921 0.005533242 0.03495874 Low-group

ERICH5 1.278928502 0.005564147 0.035120228 High-group

LRRC19 -1.678926617 0.005578325 0.035198447 Low-group

BIRC7 -1.728547974 0.005610058 0.035343147 Low-group

DBH -1.317217238 0.005610223 0.035343147 Low-group

TTBK1 -1.169683752 0.005619237 0.035388628 Low-group

CA5A -1.180538424 0.005633191 0.035465177 Low-group

C4BPA -1.115849343 0.005647052 0.035529743 Low-group

ITPKA 1.215474531 0.005649458 0.035533543 High-group

SERPINB5 -2.79042772 0.005661653 0.035598886 Low-group

TBC1D10C 1.100811728 0.005664459 0.035605168 High-group

CYP2S1 1.373975704 0.005666615 0.035607368 High-group

RIMBP3C 1.545072062 0.005669705 0.035615433 High-group

FGF9 2.11270732 0.005674141 0.035630784 High-group

FCGBP 1.416784386 0.005700906 0.035731721 High-group

C10orf71 -2.536683309 0.005704812 0.035744837 Low-group

CLDN4 1.542812738 0.005743284 0.035925443 High-group

TNNT1 2.004200708 0.005746394 0.035925443 High-group

KLK15 -2.307285768 0.00575172 0.035935831 Low-group

BEX2 1.581197839 0.005760385 0.035955894 High-group

SAA1 -2.146805051 0.005796935 0.036115417 Low-group

HIST1H2BG 1.272136858 0.005813497 0.036195722 High-group

DLGAP1 1.261404866 0.005817947 0.036211987 High-group

CFD -1.274723602 0.005835231 0.03629665 Low-group

CPNE7 1.451957116 0.005839873 0.03631406 High-group

ZNF813 1.114460046 0.005891863 0.036602711 High-group

PCDHA8 2.456697263 0.005902752 0.036658803 High-group

ACSL5 -1.051857161 0.005912346 0.036687842 Low-group

HS3ST5 2.905261963 0.005922237 0.036721971 High-group

MSLN -1.36495897 0.00592647 0.036736661 Low-group

ZMAT4 -3.23890411 0.006002045 0.037128458 Low-group

ZNF626 1.100810687 0.006011203 0.037168393 High-group

FGG -1.008981253 0.006031789 0.037260617 Low-group

FREM1 1.967665378 0.006060863 0.037381644 High-group

TMEM132B -1.205678358 0.006076445 0.037442603 Low-group

SNCA -1.170362791 0.006080889 0.037458274 Low-group

IL18 1.167953863 0.006129626 0.03768783 High-group

ATP1A4 -2.250406129 0.006131603 0.037688234 Low-group

UTS2B 1.233100663 0.006136291 0.037705287 High-group

LAMC2 1.749214642 0.006151395 0.03777455 High-group

ANKRD33B 1.276237135 0.006160008 0.037815655 High-group

ZNF177 1.86931691 0.006172496 0.037854661 High-group

TNF 1.337268577 0.006192813 0.037957939 High-group

CLDN2 -1.673725928 0.006209029 0.038045502 Low-group

SFTPA2 1.875254054 0.006257855 0.038285179 High-group

OTC -1.250589439 0.006289844 0.038457011 Low-group

BLK 1.554101044 0.006299961 0.038494989 High-group

GGT2 1.522716022 0.006320978 0.038587522 High-group

GADL1 -2.103763548 0.006337713 0.038665737 Low-group

SPRR3 -2.562447049 0.006363128 0.038808782 Low-group

ADAMTS15 -1.38315892 0.006368595 0.038830113 Low-group

STON1 -1.110497958 0.006390612 0.038937531 Low-group

RAB39B 1.077658795 0.006392139 0.038937531 High-group

GDF15 1.129462021 0.006396302 0.038938824 High-group

TTLL10 -1.073349892 0.006401824 0.038948381 Low-group

SCGB3A1 -1.232955849 0.006446095 0.03914523 Low-group

HMGCS2 -1.029828991 0.006455683 0.039191383 Low-group

SLC2A12 -1.255400353 0.006459297 0.039197038 Low-group

D4S234E -1.474870675 0.006460591 0.039197038 Low-group

POSTN 1.299787576 0.006465769 0.039198966 High-group

CLEC2L 2.822260164 0.00647187 0.039198966 High-group

CAMK1G -1.25698205 0.006477913 0.039217634 Low-group

KRT35 -3.465400444 0.006484145 0.039231265 Low-group

SYNPO2L -1.392940879 0.006500357 0.039281124 Low-group

ASIC2 -1.443527603 0.006523281 0.039407574 Low-group

SPATS1 -2.483032697 0.006536593 0.039475897 Low-group

PINLYP -1.115376964 0.006540737 0.039488825 Low-group

TMPRSS13 1.347271904 0.006575347 0.039635381 High-group

MYT1L -2.435418307 0.006577077 0.039635381 Low-group

BMP10 -2.606911145 0.006596816 0.039742187 Low-group

CDKL4 1.468951657 0.006655838 0.040036579 High-group

APCDD1L 1.627849483 0.006676622 0.040124869 High-group

FAM154A -1.113161663 0.006698519 0.040232482 Low-group

DNAAF1 -1.751422664 0.006705385 0.040256751 Low-group

VCAN 1.29741134 0.00672986 0.040353762 High-group

GALNT9 -1.956511437 0.00674766 0.040440826 Low-group

OR2C1 -1.222103301 0.006753319 0.040448222 Low-group

AF165138.7 1.801758385 0.00677462 0.040540772 High-group

FOXQ1 1.508994661 0.006814132 0.040690749 High-group

GCM1 -1.951030622 0.006839415 0.040829363 Low-group

TMEM200C 1.198345016 0.006856103 0.040904208 High-group

ADAM2 3.152958266 0.006882997 0.041052234 High-group

AGXT -1.108664052 0.00688849 0.041055105 Low-group

C17orf99 1.799547614 0.006889106 0.041055105 High-group

GJA3 1.431805921 0.006889726 0.041055105 High-group

PDIA2 -2.104680846 0.006899411 0.04107556 Low-group

METTL24 1.363835324 0.006906239 0.041101264 High-group

CCDC64B 2.068346338 0.006910227 0.041101264 High-group

MDS2 1.135670848 0.006911511 0.041101264 High-group

REG3A -3.770569528 0.006912068 0.041101264 Low-group

SERPINB11 2.200078894 0.006918244 0.041113182 High-group

CFAP99 -1.349540535 0.006941647 0.041222325 Low-group

DOK5 -1.125153988 0.006944975 0.041222325 Low-group

PTK6 1.248933126 0.007001969 0.041485665 High-group

ADRBK2 -1.123158987 0.007037728 0.041672481 Low-group

VWDE 1.867753125 0.007056446 0.041755052 High-group

CLVS1 -1.32053736 0.007059596 0.041755052 Low-group

FBN3 1.68532653 0.007074322 0.041813804 High-group

CYP11A1 -1.544299828 0.007117473 0.041993304 Low-group

HRG -1.452643349 0.00715547 0.042167002 Low-group

GPR18 1.379185479 0.007158987 0.04216833 High-group

PAX2 -2.316685843 0.007163403 0.04216833 Low-group

TEKT3 -1.584864684 0.007164252 0.04216833 Low-group

CTNND2 2.251145619 0.007207906 0.04237613 High-group

PLGLB2 -1.164890993 0.007221755 0.042443419 Low-group

NETO1 -1.912872086 0.007243019 0.042525552 Low-group

TSPAN5 -1.350910204 0.007244359 0.042525552 Low-group

TFAP2E 1.077523278 0.007270725 0.042651419 High-group

S100P 1.884110339 0.007275687 0.042651419 High-group

GRHL2 1.979565168 0.007282138 0.042671076 High-group

PTPN13 1.402341739 0.007296881 0.042719372 High-group

GGT5 -1.10492881 0.007357736 0.043008468 Low-group

NTF3 -1.172438166 0.007361698 0.043008468 Low-group

FAM24B 1.022425074 0.007362479 0.043008468 High-group

SYNE4 1.037262727 0.00736545 0.043008468 High-group

KRT34 -2.118906753 0.007365898 0.043008468 Low-group

MARCO -1.513679353 0.007369532 0.043016945 Low-group

TUBAL3 2.036171338 0.007373218 0.043025719 High-group

NYAP2 1.955556945 0.007423367 0.043267125 High-group

RASSF9 1.462083165 0.00744651 0.043377417 High-group

SOAT2 1.750716352 0.007452006 0.043395553 High-group

OVOL2 1.634426463 0.007494812 0.043619056 High-group

PCDHA2 1.888332815 0.007507514 0.04365431 High-group

ZNF560 3.050841379 0.007591579 0.044104101 High-group

LCE1E 2.077903777 0.007602133 0.044120513 High-group

TP63 -1.580424714 0.00760856 0.044137708 Low-group

CYP11B1 1.979241641 0.007616584 0.044171258 High-group

DES -1.734893057 0.007629609 0.044207776 Low-group

APBA2 1.331056507 0.007660402 0.044319695 High-group

IGFBPL1 2.00231724 0.007672756 0.04436649 High-group

VSX2 2.515129328 0.007681055 0.04438048 High-group

TAC3 1.471112429 0.00768193 0.04438048 High-group

GAREML 1.0255056 0.007684534 0.044382519 High-group

RGS21 1.981505944 0.007705295 0.044477938 High-group

ALPL -1.356525327 0.007734172 0.044603863 Low-group

GRAMD2 1.474727917 0.007762418 0.044727503 High-group

CLIC5 -1.119853177 0.007781727 0.044799477 Low-group

GRIN2A 1.946068887 0.007789605 0.044810875 High-group

RBMY1F -2.715139111 0.007790527 0.044810875 Low-group

PLA2G4E 1.520647761 0.007804441 0.044825773 High-group

KMO -1.121605486 0.007817778 0.044862894 Low-group

H3F3C -1.05206684 0.007838694 0.044943669 Low-group

ANKS1B 1.307316271 0.007865095 0.045035411 High-group

CLIP3 -1.287570098 0.007866118 0.045035411 Low-group

TMEM40 1.631157719 0.00787399 0.045060726 High-group

LRRC39 -1.081028929 0.007878238 0.045065534 Low-group

GGT1 1.018112753 0.007957288 0.045425442 High-group

FGB -1.046057811 0.00797055 0.045487978 Low-group

ASPG -1.433869223 0.007986865 0.045567899 Low-group

UCN2 1.613366192 0.008014354 0.045685063 High-group

STEAP1B 1.039742707 0.008033236 0.045766227 High-group

SPATA22 -2.290389455 0.008049956 0.045808525 Low-group

PALM2 -1.060037838 0.008069177 0.045891769 Low-group

ULBP1 1.465899675 0.008069241 0.045891769 High-group

OTOS 3.454049292 0.008127097 0.046127654 High-group

UGT1A8 1.765231568 0.00815085 0.04623302 High-group

LTA 1.141890951 0.008182345 0.046333903 High-group

C8orf34 2.120568707 0.008195796 0.046384019 High-group

CSF3R 1.042463485 0.008221878 0.046514079 High-group

CNTN1 -2.566171476 0.008260941 0.046655799 Low-group

HOXA7 -1.954829408 0.008290851 0.0467475 Low-group

BLID -2.193360996 0.008323248 0.046889928 Low-group

ADM2 1.069777935 0.008370899 0.047095908 High-group

HPD -1.457862757 0.008371756 0.047095908 Low-group

GSTA5 -1.40328926 0.008419011 0.047260338 Low-group

FXYD2 2.022108433 0.008440005 0.047321112 High-group

ZSWIM5 1.092154541 0.008441688 0.047321112 High-group

ONECUT3 -1.688659877 0.008446055 0.047321112 Low-group

PGF -1.145281623 0.008450902 0.047325052 Low-group

FGF3 2.801555246 0.008486927 0.047486318 High-group

PCDHGB2 -1.330580484 0.008496853 0.047514883 Low-group

C6orf141 1.80958381 0.008515086 0.047553237 High-group

TRIM16 1.151019846 0.008564592 0.047785237 High-group

BTLA 1.483577866 0.008571737 0.047811565 High-group

GPR128 -1.190342712 0.008574929 0.04781584 Low-group

TMEM249 1.077083016 0.008594585 0.047884813 High-group

PPBP -1.821913457 0.008610153 0.047944443 Low-group

TMEM54 1.027587206 0.008617007 0.047969058 High-group

TAF4B 1.099192089 0.008628149 0.047993178 High-group

FBXW12 1.226291404 0.008628644 0.047993178 High-group

C2CD4C 1.568160995 0.008663669 0.048160811 High-group

PRKCB 1.033321658 0.008690031 0.048252926 High-group

GPR84 1.187911489 0.008722711 0.048420749 High-group

HES2 1.33861958 0.008748269 0.048548954 High-group

C12orf40 2.102381674 0.008770811 0.048620778 High-group

NKAIN1 1.698600066 0.008773544 0.048620778 High-group

SOSTDC1 1.564590296 0.008780293 0.048644499 High-group

RPA4 1.21461589 0.00880646 0.048750964 High-group

GJA10 -2.874415103 0.008806929 0.048750964 Low-group

TNXB -1.043209066 0.008884221 0.049096095 Low-group

MLN 3.493716757 0.008898993 0.049135248 High-group

MIA-RAB4B 1.864787585 0.008901277 0.049135248 High-group

GPLD1 -1.510743282 0.008915454 0.049161997 Low-group

OR2H2 2.427693051 0.008931834 0.049218008 High-group

ACADL -1.458059012 0.008938425 0.049218008 Low-group

APOA4 2.531724413 0.008943735 0.049218008 High-group

SBK2 1.74992466 0.008953275 0.049243984 High-group

ATOH8 -1.137119987 0.008955763 0.049243984 Low-group

HOXB7 1.31077881 0.00895595 0.049243984 High-group

H2BFWT -2.539629493 0.008978426 0.049340044 Low-group

DPCR1 -2.953432547 0.009036426 0.049603461 Low-group

ANKRD7 2.237865718 0.009051385 0.049657919 High-group

UROC1 -1.626105476 0.009082099 0.049750477 Low-group

ZNF578 1.28713514 0.009108619 0.049860897 High-group

ASPDH -1.177117936 0.009118162 0.04989928 Low-group
