# Supplementary figures and images for: A Novel Metabolism-Related Gene Signature for Predicting the Prognosis of HBV-Infected Hepatocellular Carcinoma
Source: J Oncol. 2022 Aug 28;2022:2391265. doi: 10.1155/2022/2391265 (PMC9441393; doi:10.1155/2022/2391265)

Scale Free Topology Model Fit

Scale independence

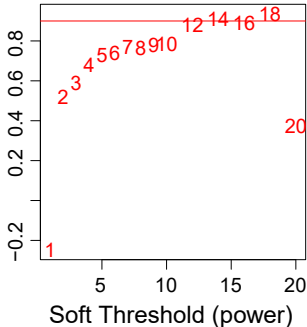

Mean connectivity

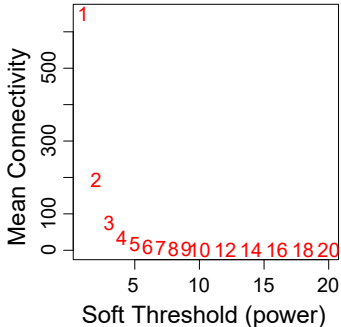

Supplement: Supplementary Materials — Supplementary Figure 1. Identification of coexpressed metabolic genes associated with HBV hepatitis using WGCNA. (a) Soft threshold selection in WGCNA network analysis. (b) Gene distribution in WGCNA network analysis. (c) Heatmap of topological overlap in WGCNA network analysis. Supplementary Figure 2. Validation of MRGPI models in other datasets. (a–c) Survival curves of ATIC, KIF2C, and POLR3C genes in the GSE14520 validation set. (d, e) Survival and ROC curves of the prognostic model in the GSE14520 validation set. Supplementary Table 1. Genes included in each module of WGCNA analysis. Supplementary Table 2. Differentially expressed genes in the high and low MRGPI risk groups. [file 2391265.f1.zip › SupFig1A.pdf]

# Cluster Dendrogram

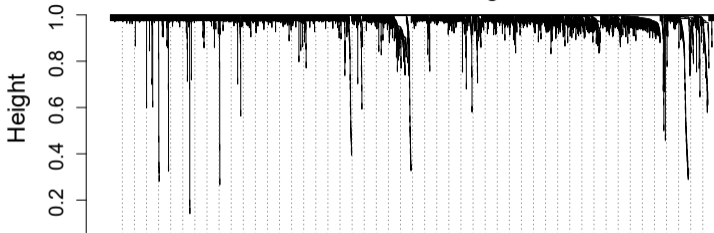

Dynamic Tree

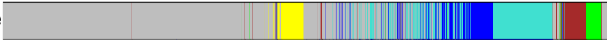

Merged dynamic

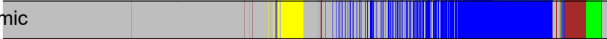

Supplement: Supplementary Materials — Supplementary Figure 1. Identification of coexpressed metabolic genes associated with HBV hepatitis using WGCNA. (a) Soft threshold selection in WGCNA network analysis. (b) Gene distribution in WGCNA network analysis. (c) Heatmap of topological overlap in WGCNA network analysis. Supplementary Figure 2. Validation of MRGPI models in other datasets. (a–c) Survival curves of ATIC, KIF2C, and POLR3C genes in the GSE14520 validation set. (d, e) Survival and ROC curves of the prognostic model in the GSE14520 validation set. Supplementary Table 1. Genes included in each module of WGCNA analysis. Supplementary Table 2. Differentially expressed genes in the high and low MRGPI risk groups. [file 2391265.f1.zip › SupFig1B.pdf]

Network heatmap plot, all genes

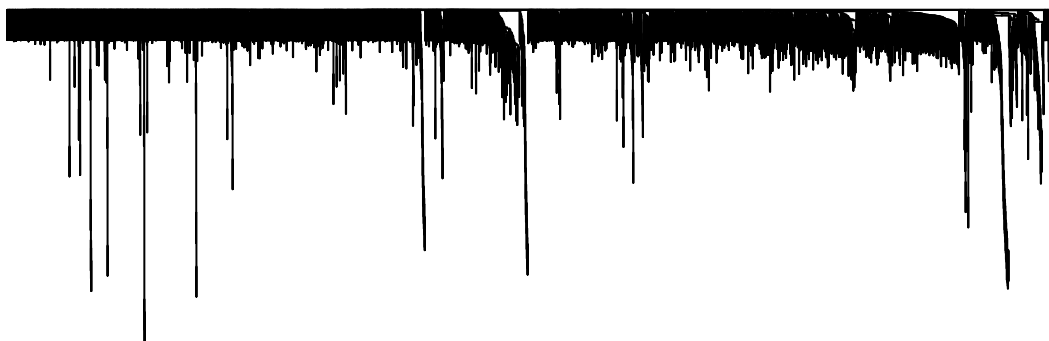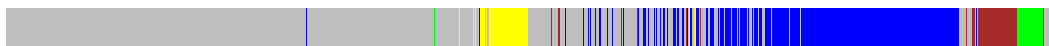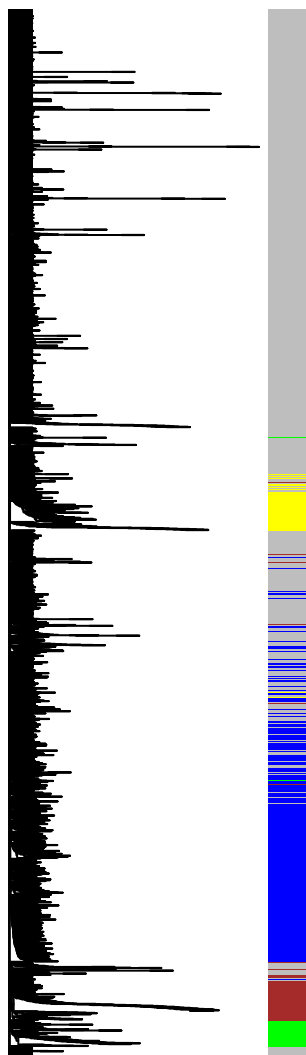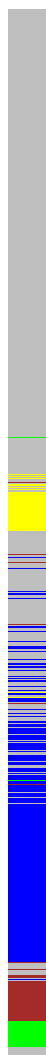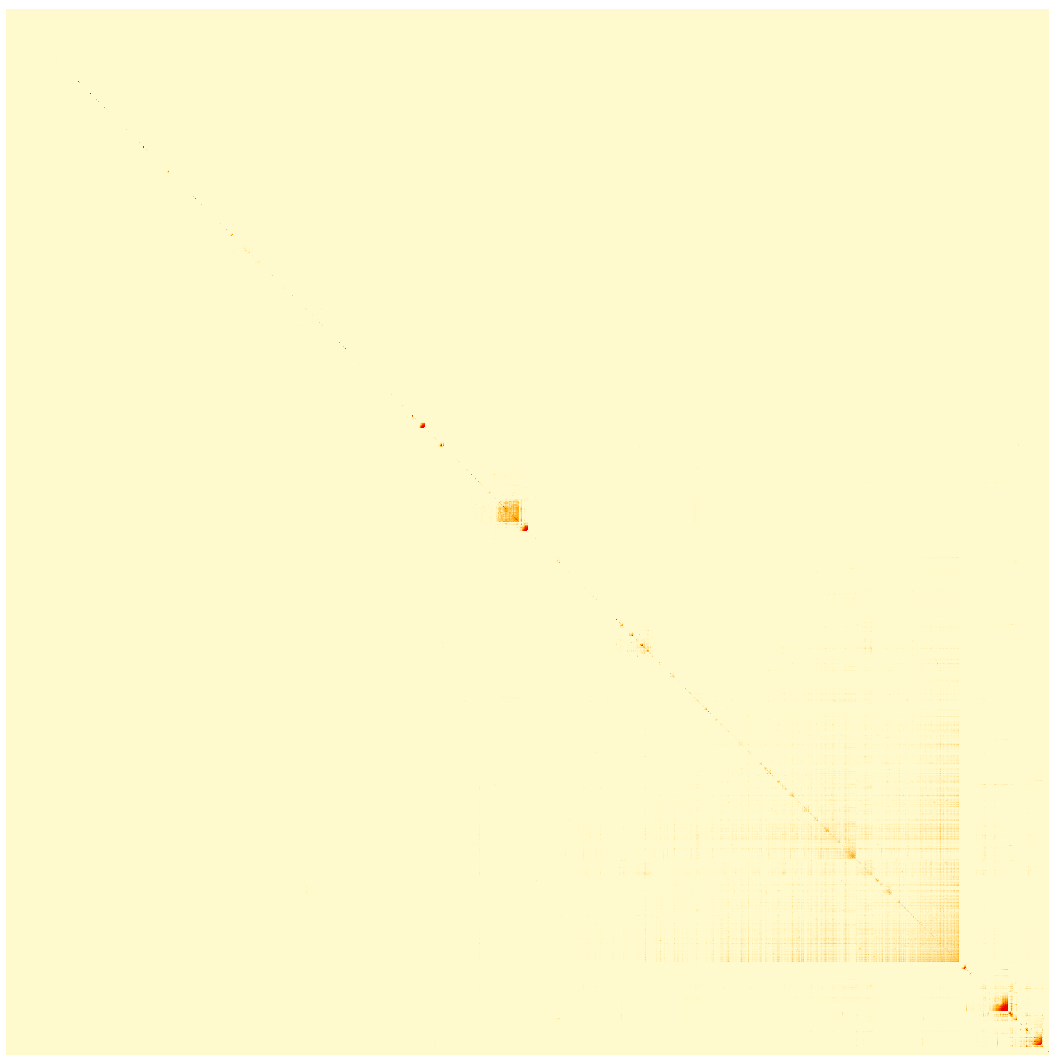

Supplement: Supplementary Materials — Supplementary Figure 1. Identification of coexpressed metabolic genes associated with HBV hepatitis using WGCNA. (a) Soft threshold selection in WGCNA network analysis. (b) Gene distribution in WGCNA network analysis. (c) Heatmap of topological overlap in WGCNA network analysis. Supplementary Figure 2. Validation of MRGPI models in other datasets. (a–c) Survival curves of ATIC, KIF2C, and POLR3C genes in the GSE14520 validation set. (d, e) Survival and ROC curves of the prognostic model in the GSE14520 validation set. Supplementary Table 1. Genes included in each module of WGCNA analysis. Supplementary Table 2. Differentially expressed genes in the high and low MRGPI risk groups. [file 2391265.f1.zip › SupFig1C.pdf]

Survival probability

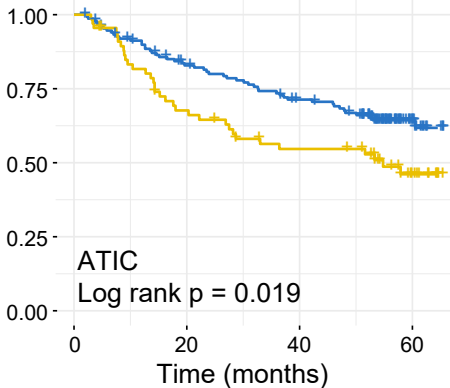

Low Expression (151) High Expression (67)

Supplement: Supplementary Materials — Supplementary Figure 1. Identification of coexpressed metabolic genes associated with HBV hepatitis using WGCNA. (a) Soft threshold selection in WGCNA network analysis. (b) Gene distribution in WGCNA network analysis. (c) Heatmap of topological overlap in WGCNA network analysis. Supplementary Figure 2. Validation of MRGPI models in other datasets. (a–c) Survival curves of ATIC, KIF2C, and POLR3C genes in the GSE14520 validation set. (d, e) Survival and ROC curves of the prognostic model in the GSE14520 validation set. Supplementary Table 1. Genes included in each module of WGCNA analysis. Supplementary Table 2. Differentially expressed genes in the high and low MRGPI risk groups. [file 2391265.f1.zip › SupFig2A.pdf]

Survival probability

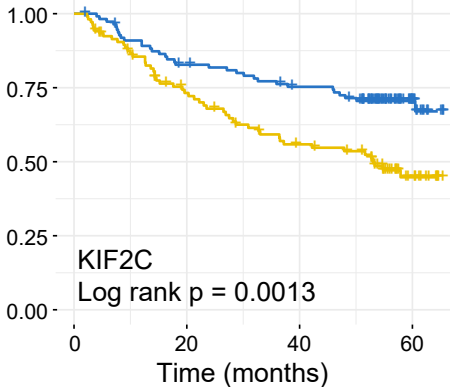

Low Expression (112) High Expression (106)

Supplement: Supplementary Materials — Supplementary Figure 1. Identification of coexpressed metabolic genes associated with HBV hepatitis using WGCNA. (a) Soft threshold selection in WGCNA network analysis. (b) Gene distribution in WGCNA network analysis. (c) Heatmap of topological overlap in WGCNA network analysis. Supplementary Figure 2. Validation of MRGPI models in other datasets. (a–c) Survival curves of ATIC, KIF2C, and POLR3C genes in the GSE14520 validation set. (d, e) Survival and ROC curves of the prognostic model in the GSE14520 validation set. Supplementary Table 1. Genes included in each module of WGCNA analysis. Supplementary Table 2. Differentially expressed genes in the high and low MRGPI risk groups. [file 2391265.f1.zip › SupFig2B.pdf]

Survival probability

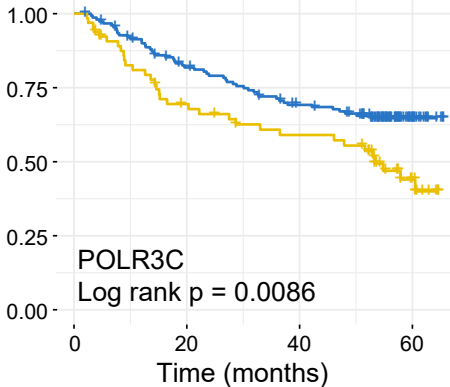

Low Expression (153) High Expression (65)

Supplement: Supplementary Materials — Supplementary Figure 1. Identification of coexpressed metabolic genes associated with HBV hepatitis using WGCNA. (a) Soft threshold selection in WGCNA network analysis. (b) Gene distribution in WGCNA network analysis. (c) Heatmap of topological overlap in WGCNA network analysis. Supplementary Figure 2. Validation of MRGPI models in other datasets. (a–c) Survival curves of ATIC, KIF2C, and POLR3C genes in the GSE14520 validation set. (d, e) Survival and ROC curves of the prognostic model in the GSE14520 validation set. Supplementary Table 1. Genes included in each module of WGCNA analysis. Supplementary Table 2. Differentially expressed genes in the high and low MRGPI risk groups. [file 2391265.f1.zip › SupFig2C.pdf]

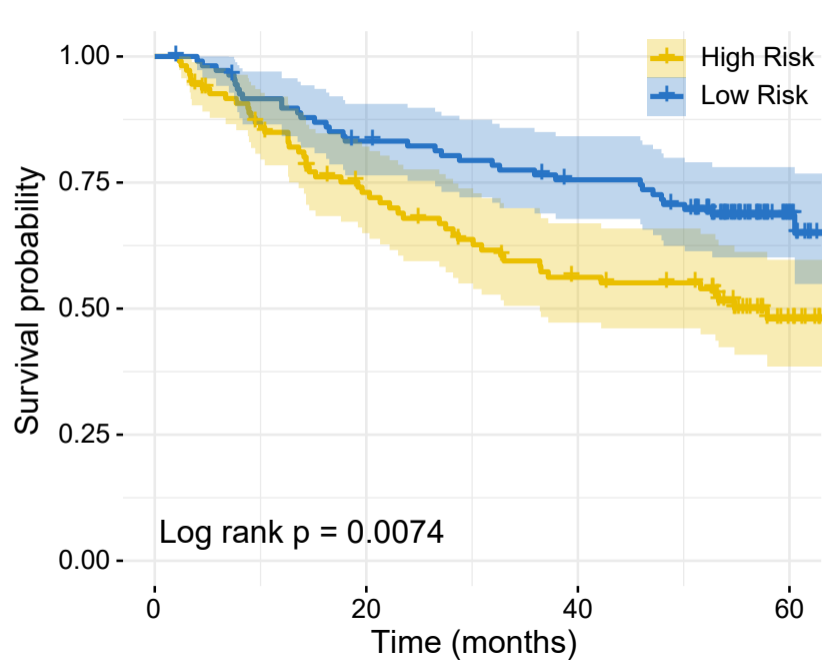

Supplement: Supplementary Materials — Supplementary Figure 1. Identification of coexpressed metabolic genes associated with HBV hepatitis using WGCNA. (a) Soft threshold selection in WGCNA network analysis. (b) Gene distribution in WGCNA network analysis. (c) Heatmap of topological overlap in WGCNA network analysis. Supplementary Figure 2. Validation of MRGPI models in other datasets. (a–c) Survival curves of ATIC, KIF2C, and POLR3C genes in the GSE14520 validation set. (d, e) Survival and ROC curves of the prognostic model in the GSE14520 validation set. Supplementary Table 1. Genes included in each module of WGCNA analysis. Supplementary Table 2. Differentially expressed genes in the high and low MRGPI risk groups. [file 2391265.f1.zip › SupFig2D.pdf]

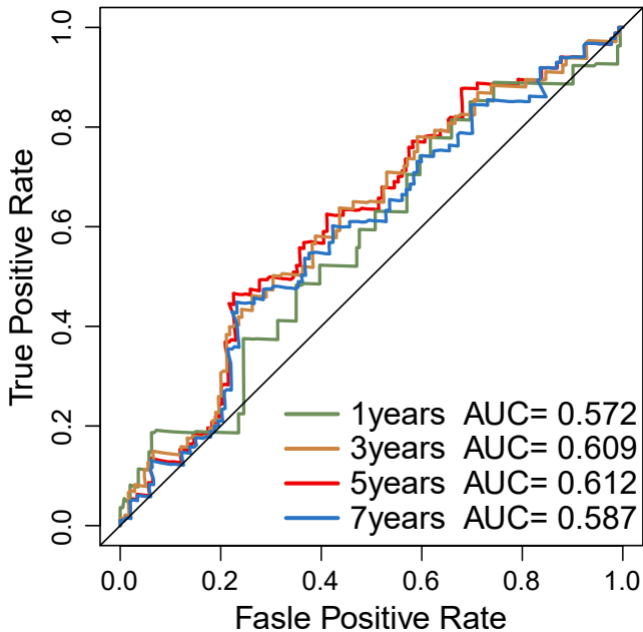

Supplement: Supplementary Materials — Supplementary Figure 1. Identification of coexpressed metabolic genes associated with HBV hepatitis using WGCNA. (a) Soft threshold selection in WGCNA network analysis. (b) Gene distribution in WGCNA network analysis. (c) Heatmap of topological overlap in WGCNA network analysis. Supplementary Figure 2. Validation of MRGPI models in other datasets. (a–c) Survival curves of ATIC, KIF2C, and POLR3C genes in the GSE14520 validation set. (d, e) Survival and ROC curves of the prognostic model in the GSE14520 validation set. Supplementary Table 1. Genes included in each module of WGCNA analysis. Supplementary Table 2. Differentially expressed genes in the high and low MRGPI risk groups. [file 2391265.f1.zip › SupFig2E.pdf]
